# Microscopic derivation of Ginzburg–Landau theory and the BCS critical temperature shift in general external fields

**DOI:** 10.1007/s00526-023-02539-x

**Published:** 2023-07-29

**Authors:** Andreas Deuchert, Christian Hainzl, Marcel Oliver Maier

**Affiliations:** 1 Institut für Mathematik, Universität Zürich, Winterthurerstrasse 190, 8057 Zurich, Switzerland; 2 Mathematisches Institut der Universität München, Theresienstr. 39, 80333 Munchen, Germany

## Abstract

We consider the Bardeen–Cooper–Schrieffer (BCS) free energy functional with weak and macroscopic external electric and magnetic fields and derive the Ginzburg–Landau functional. We also provide an asymptotic formula for the BCS critical temperature as a function of the external fields. This extends our previous results in Deuchert et al. (Microscopic derivation of Ginzburg-Landau theory and the BCS critical temperature shift in a weak homogeneous magnetic field, PMP **4**(1), 1–89 (2023)) for the constant magnetic field to general magnetic fields with a nonzero magnetic flux through the unit cell.

## Introduction and main results

### Introduction

Ginzburg–Landau (GL) theory has been introduced as the first macroscopic and phenomenlogical description of superconductivity in 1950 [29]. The theory comprises a system of partial differential equations for a complex-valued function, the order parameter, and an effective magnetic field. Ginzburg–Landau theory has been highly influential and investigated in numerous works, among which are [1, 7, 8, 10–13, 45, 46] and references therein.

Bardeen–Cooper–Schrieffer (BCS) theory of superconductivity is the first commonly accepted and Nobel prize awarded microscopic theory of superconductivity [2]. As a major breakthrough, the theory features a pairing mechanism between the electrons below a certain critical temperature, which causes the electrical resistance in the system to drop to zero in the superconducting phase. This effect is due to an effective attraction between the electrons, which arises as a consequence of the phonon vibrations of the lattice ions in the superconductor.

One way to formulate BCS theory mathematically is via the BCS free energy functional or BCS functional for short. As Leggett pointed out in [38], the BCS functional can be obtained from a full quantum mechanical description of the system by restricting attention to quasi-free states, see also [14]. Such states are determined by their one-particle density matrix and the Cooper pair wave function. The BCS functional has been studied intensively from a mathematical point of view in the absence of external fields in [4, 17, 20, 21, 27, 31, 32, 36] and in the presence of external fields in [5, 6, 16, 25, 33]. The BCS gap equation arises as the Euler–Lagrange equation of the BCS functional and its solution is used to compute the spectral gap of an effective Hamiltonian, which is open in the superconducting phase. BCS theory from the point of view of its gap equation is studied in [3, 41, 43, 50–52].

The present article continues a series of works, in which the *macroscopic* GL theory is derived from the *microscopic* BCS theory in a regime close to the critical temperature and for weak external fields. This endeavor has been initiated by Gor’kov in 1959 [30]. The first mathematically rigorous derivation of the GL functional from the BCS functional has been provided by Frank, Hainzl, Seiringer, and Solovej for periodic external electric and magnetic fields in 2012 in [23]. An important assumption of this work is that the flux of the external magnetic field through the unit cell of periodicity of the system vanishes. This excludes for example a homogeneous magnetic field. The techniques from this GL derivation have been further developed in [24] to compute the BCS critical temperature shift caused by the external fields. The first important step towards overcoming the zero magnetic flux restriction in [23, 24] has been made by Frank, Hainzl, and Langmann, who considered in [26] the problem of computing the BCS critical temperature shift for systems exposed to a homogeneous magnetic field within the framework of linearized BCS theory. Recently, the derivation of the GL functional and the computation of BCS critical temperature shift (for the full nonlinear model) could be extended to the case of a constant magnetic field by Deuchert, Hainzl, and Maier in [15]. The goal of the present work is to further extend the results in [15] to the case of general external magnetic fields with an arbitrary flux through the unit cell.

GL theory arises from BCS theory when the temperature is sufficiently close to the critical temperature and when the external fields are weak and slowly varying. More precisely, if $0 < h \ll 1$ denotes the ratio between the microscopic and the macroscopic length scale, then the external electric field *W* and the magnetic vector potential ${{\textbf {A}}}$ are given by $h^2 W(hx)$ and $h{{\textbf {A}}}(hx)$, respectively. Furthermore, the temperature regime is such that $T - {T_{\text {c}}}= -{T_{\text {c}}}Dh^2$ for some constant $D >0$, where ${T_{\text {c}}}$ is the critical temperature in absence of external fields. When this scaling is in effect, it is shown in [15, 23] that the Cooper pair wave function $\alpha (x,y)$ is given by

1.1 $$\begin{aligned} \alpha (x,y) = h\, \alpha _*(x - y) \, \psi \left( \frac{h(x+y)}{2}\right) \end{aligned}$$

to leading order in *h*. Here, $\alpha _*$ is the microscopic Cooper pair wave function in the absence of external fields and $\psi$ is the GL order parameter.

Moreover, the influence of the external fields causes a shift in the critical temperature of the BCS model, which is described by linearized GL theory in the same scaling regime. More precisely, it has been shown in [15, 24, 26] that the critical temperature shift in BCS theory is given by

1.2 $$\begin{aligned} {T_{\text {c}}}(h) = {T_{\text {c}}}(1 - {D_{\text {c}}}h^2) \end{aligned}$$

to leading order, where ${D_{\text {c}}}$ denotes a critical parameter that can be computed using linearized GL theory.

The present work is an extension of the paper [15], where the case of a constant magnetic field was considered. In this article, we incorporate periodic electric fields *W* and general vector potentials ${{\textbf {A}}}$ that give rise to periodic magnetic fields. This, in particular, generalizes the results in [23, 24] to the case of general external magnetic fields with non-zero flux through the unit cell. We show that within the scaling introduced above, the Ginzburg–Landau energy arises as leading order correction on the order $h^4$. Furthermore, we show that the Cooper pair wave function admits the leading order term (1.1) and that the critical temperature shift is given by (1.2) to leading order. The main technical novelty of this article is a further development of the phase approximation method, which has been pioneered in the framework of BCS theory for the case of the constant magnetic field in [15, 26]. It allows us to compute the BCS energy of a class of trial states (Gibbs states) in a controlled way. This trial state analysis is later used in the proofs of the upper and of the lower bound for the BCS free energy. The proof of our lower bound additionally uses a priori bounds for certain low-energy BCS states that include the magnetic field and have been established in [15].

### Gauge-periodic samples

Our objective is to study a system of three-dimensional fermionic particles that is subject to weak and slowly varying external electromagnetic fields within the framework of BCS theory. Let us define the magnetic field ${{\textbf {B}}}{:}{=}h^2 e_3$. It can be written in terms of the vector potential ${{\textbf {A}}}_{{{\textbf {B}}}}(x) {:}{=}\frac{1}{2} {{\textbf {B}}}\wedge x$, where $x \wedge y$ denotes the cross product of two vectors $x,y \in {\mathbb {R}}^3$, as ${{\textbf {B}}}= {{\,\textrm{curl}\,}}{{\textbf {A}}}_{{\textbf {B}}}$. To the vector potential ${{\textbf {A}}}_{{{\textbf {B}}}}$ we associate the magnetic translations

1.3 $$\begin{aligned} T(v)f(x)&{:}{=}\, \text {e}^{\text {i}\frac{{{\textbf {B}}}}{2}\cdot (v\wedge x)} f(x+v),&v&\in {\mathbb {R}}^3, \end{aligned}$$

which commute with the magnetic momentum operator $-\text {i}\nabla + {{\textbf {A}}}_{{\textbf {B}}}$. The family $\{ T(v) \}_{v\in {\mathbb {R}}^3}$ satisfies $T(v+w) = \text {e}^{\text {i}\frac{{{\textbf {B}}}}{2} \cdot (v \wedge w)} T(v) T(w)$ and is therefore a unitary representation of the Heisenberg group. We assume that our system is periodic with respect to the Bravais lattice $\Lambda _h {:}{=}\sqrt{2\pi } \, h^{-1} \, {\mathbb {Z}}^3$ with fundamental cell

1.4 $$\begin{aligned} Q_h&{:}{=}\, \bigl [0, \sqrt{2\pi } \, h^{-1}\bigr ]^3 \subseteq {\mathbb {R}}^3. \end{aligned}$$

We also introduce the notation $\Lambda {:}{=}\Lambda _1$ and $Q {:}{=}Q_1$. Let $b_i = \sqrt{2\pi } \, h^{-1} \, e_i$ denote the basis vectors that span $\Lambda _h$. The magnetic flux through the face of the unit cell spanned spanned by $b_1$ and $b_2$ equals $2 \pi$, and hence the abelian subgroup $\{ T(\lambda ) \}_{\lambda \in \Lambda _h}$ is a unitary representation of the lattice group.

Our system is subject to an external electric field $W_h(x) = h^2W(hx)$ with a fixed function $W :{\mathbb {R}}^3 \rightarrow {\mathbb {R}}$, as well as a magnetic field defined in terms of the vector potential ${{\textbf {A}}}_h(x) = h {{\textbf {A}}}(hx)$, which admits the form ${{\textbf {A}}}{:}{=}{{\textbf {A}}}_{e_3} + A$ with $A :{\mathbb {R}}^3\rightarrow {\mathbb {R}}^3$ and ${{\textbf {A}}}_{e_3}$ as defined above. We assume that *A* and *W* are periodic with respect to $\Lambda$. The flux of the magnetic field ${{\,\textrm{curl}\,}}A_h$ through all faces of the unit cell $Q_h$ vanishes because $A_h$ is a periodic function. Accordingly, the magnetic field ${{\,\textrm{curl}\,}}{{\textbf {A}}}_h$ has the same fluxes through the faces of the unit cell as ${{\textbf {B}}}$.

The above representation of ${{\textbf {A}}}_h$ is general in the sense that any periodic magnetic field field *B*(*x*) that satisfies the Maxwell equation ${{\,\textrm{div}\,}}B = 0$ can be written as the curl of a vector potential $A_B$ of the form $A_B(x)= \frac{1}{2} b \wedge x + A_{\text {per}}(x)$, where *b* denotes the vector with components given by the average magnetic flux of *B* through the faces of $Q_h$ and $A_{\text {per}}$ is a periodic vector potential. For more information concerning this decomposition we refer to [40, Chapter 4]. For a treatment of the two-dimensional case, see [49].

### The BCS functional

In BCS theory a state is conveniently described by its generalized one-particle density matrix, that is, by a self-adjoint operator $\Gamma$ on $L^2({\mathbb {R}}^3) \oplus L^2({\mathbb {R}}^3)$, which obeys $0 \leqslant \Gamma \leqslant 1$ and is of the form

1.5 $$\begin{aligned} \Gamma = \begin{pmatrix} \gamma &{} \alpha \\ \overline{\alpha }&{} 1 - \overline{\gamma }\end{pmatrix}. \end{aligned}$$

Here, $\overline{\alpha }$ denotes the operator $\alpha$ with the complex conjugate integral kernel in the position space representation. Since $\Gamma$ is self-adjoint we know that $\gamma$ is self-adjoint and that $\alpha$ is symmetric in the sense that its integral kernel satisfies $\alpha (x,y) = \alpha (y,x)$. This symmetry is related to the fact that we exclude spin degrees of freedom from our description and assume that all Cooper pairs are in a spin singlet state. The condition $0 \leqslant \Gamma \leqslant 1$ implies that the one-particle density matrix $\gamma$ satisfies $0 \leqslant \gamma \leqslant 1$ and that $\alpha$ and $\gamma$ are related through the inequality

1.6 $$\begin{aligned} \alpha \alpha ^* \leqslant \gamma ( 1- \gamma ). \end{aligned}$$

Let us define the magnetic translations ${{\textbf {T}}}(\lambda )$ on $L^2({\mathbb {R}}^3)\oplus L^2({\mathbb {R}}^3)$ by

$$\begin{aligned} {{\textbf {T}}}(v)&{:}{=}\begin{pmatrix} T(v) &{} 0 \\ 0 &{} \overline{T(v)}\end{pmatrix},&v&\in {\mathbb {R}}^3. \end{aligned}$$

We say that a BCS state $\Gamma$ is *gauge-periodic* provided ${{\textbf {T}}}(\lambda ) \, \Gamma \, {{\textbf {T}}}(\lambda )^* = \Gamma$ holds for any $\lambda \in \Lambda _h$. This implies the relations $T(\lambda ) \, \gamma \, T(\lambda )^* = \gamma$ and $T(\lambda )\,\alpha \,\overline{T(\lambda )}^* = \alpha$, or, in terms of integral kernels,

1.7 $$\begin{aligned} \gamma (x, y)&= \text {e}^{\text {i}\frac{{{\textbf {B}}}}{2} \cdot (\lambda \wedge (x-y))} \; \gamma (x+\lambda ,y+ \lambda ), \nonumber \\ \alpha (x, y)&= \text {e}^{\text {i}\frac{{{\textbf {B}}}}{2} \cdot (\lambda \wedge (x+y))} \; \alpha (x+\lambda ,y+ \lambda ),&\lambda \in \Lambda _h. \end{aligned}$$

We further say that a gauge-periodic BCS state $\Gamma$ is *admissible* if

1.8 $$\begin{aligned} {{\,\textrm{Tr}\,}}\bigl [\gamma + (-\text {i}\nabla + {{\textbf {A}}}_{{\textbf {B}}})^2\gamma \bigr ] < \infty \end{aligned}$$

holds. Here ${{\,\textrm{Tr}\,}}[{\mathcal {R}}]$ denotes the trace per unit volume of an operator ${\mathcal {R}}$ defined by

1.9 $$\begin{aligned} {{\,\textrm{Tr}\,}}[{\mathcal {R}}]&{:}{=}\frac{1}{|Q_h|} {{\,\textrm{Tr}\,}}_{L^2(Q_h)} [\chi {\mathcal {R}}\chi ], \end{aligned}$$

where $\chi$ denotes the characteristic function of the cube $Q_h$ in (1.4) and ${{\,\textrm{Tr}\,}}_{L^2(Q_h)}[\cdot ]$ is the usual trace over an operator on $L^2(Q_h)$. By the condition in (1.8), we mean that $\chi \gamma \chi$ and $\chi (-\text {i}\nabla + {{\textbf {A}}}_{{\textbf {B}}})^2 \gamma \chi$ are trace-class operators. Equations (1.6), (1.8), and the same inequality with $\gamma$ replaced by ${\overline{\gamma }}$ imply that $\alpha$, $(-\text {i}\nabla + {{\textbf {A}}}_{{\textbf {B}}})\alpha$, and $(-\text {i}\nabla + {{\textbf {A}}}_{{\textbf {B}}}) \overline{\alpha }$ are locally Hilbert–Schmidt. We will rephrase this property as a notion of $H^1$-regularity for the kernel of $\alpha$ in Sect. 2 below.

Let $\Gamma$ be an admissible BCS state. We define the Bardeen–Cooper–Schrieffer free energy functional, or BCS functional for short, at temperature $T\geqslant 0$ by the formula

1.10 $$\begin{aligned} {\mathcal {F}}^{\text {BCS}}_{h, T}(\Gamma )&{:}{=}{{\,\textrm{Tr}\,}}\bigl [ \bigl ( (-\text {i}\nabla + {{\textbf {A}}}_h)^2 - \mu + W_h \bigr )\gamma \bigr ] - T\, S(\Gamma ) \nonumber \\&\quad - \frac{1}{|Q_h|} \int _{Q_h} \text {d}X \int _{{\mathbb {R}}^3} \text {d}r\; V(r) \, |\alpha (X,r)|^2, \end{aligned}$$

where $S(\Gamma )= - {{\,\textrm{Tr}\,}}[\Gamma \ln (\Gamma )]$ denotes the von Neumann entropy per unit volume and $\mu \in {\mathbb {R}}$ is a chemical potential. The interaction energy is written in terms of the center-of-mass and relative coordinates $X = \frac{x+y}{2}$ and $r = x-y$. Throughout this paper, we write, by a slight abuse of notation, $\alpha (x,y) \equiv \alpha (X,r)$. That is, we use the same symbol for the function depending on the original coordinates and for the one depending on *X* and *r*.

The assumption  guarantees the existence of a constant $C>0$ such that

1.11 $$\begin{aligned} {\mathcal {F}}^{\text {BCS}}_{h, T}(\Gamma )&\geqslant \frac{1}{2} {{\,\textrm{Tr}\,}}\bigl [ \gamma + (-\text {i}\nabla + {{\textbf {A}}}_{{\textbf {B}}})^2 \gamma \bigr ] - C. \end{aligned}$$

In other words, the BCS functional is bounded from below and coercive on the set of admissible BCS states.

The normal state $\Gamma _0$ is the unique minimizer of the BCS functional when restricted to admissible states with $\alpha = 0$ and reads

1.12 $$\begin{aligned} \Gamma _0&{:}{=}\begin{pmatrix} \gamma _0 &{} 0 \\ 0 &{} 1-\overline{\gamma }_0 \end{pmatrix},&\gamma _0&{:}{=}\frac{1}{1 + \text {e}^{ ((-\text {i}\nabla + {{\textbf {A}}}_h)^2 + W_h-\mu )/T}}. \end{aligned}$$

Its name is motivated by the fact that it is also the unique minimizer of the BCS functional if the temperature *T* is chosen sufficiently large. We define the BCS free energy by

1.13 $$\begin{aligned} F^{\text {BCS}}(h, T) {:}{=}\inf \bigl \{ {\mathcal {F}}^{\text {BCS}}_{h, T}(\Gamma ) - {\mathcal {F}}^{\text {BCS}}_{h, T}(\Gamma _0) : \Gamma \text { admissible}\bigr \} \end{aligned}$$

and say that the system is superconducting at temperature *T* if $F^{\text {BCS}}(h, T) < 0$. Although it is not difficult to prove that the BCS functional has a minimizer, we refrain from giving a proof here. If we assume that the BCS functional has a minimizer $\Gamma$ then the condition $F^{\text {BCS}}(h, T) < 0$ implies $\alpha = \Gamma _{12} \ne 0$.

The goal of this paper is to derive an asymptotic formula for $F^{\text {BCS}}(h, T)$ for small $h > 0$. This will allow us to derive Ginzburg–Landau theory and to show how the critical temperature depends on the external electric and magnetic field and on *h*. For our main results to hold, we need the following assumptions.

#### Assumption 1.1

We assume that the interaction potential *V* is a radial function that satisfies $(1+|\cdot |^2) V\in L^2({\mathbb {R}}^3) \cap L^\infty ({\mathbb {R}}^3)$. Moreover, the electric and the magnetic potentials $W\in W^{1, \infty }({\mathbb {R}}^3)$ and $A\in W^{3, \infty }({\mathbb {R}}^3; {\mathbb {R}}^3)$ are $\Lambda$-periodic functions, i.e., $W(x + \lambda ) = W(x)$ and $A(x + \lambda ) = A(x)$ for $\lambda \in \Lambda$ and all $x\in {\mathbb {R}}^3$. We also assume that $A(0) = 0$.

#### Remark 1.2

The regularity assumption for the vector potential *A* is natural within our framework of gauge-invariant perturbation theory in Sects. 3 and 4. We believe, however, that it could be relaxed to a certain extent. We also expect that it is possible to allow for moderate local singularities of *V*. Our motivation to work with the above assumptions is to keep the presentation at a reasonable length.

### The translation-invariant BCS functional

In the absence of external fields we describe the system by translation-invariant states, that is, we assume that the integral kernels of $\gamma$ and $\alpha$ are of the form $\gamma (x-y)$ and $\alpha (x-y)$. In this case, the trace per unit volume is defined with respect to a cube with sidelength 1. We denote the resulting translation-invariant BCS functional by ${\mathcal {F}}^{\text {BCS}}_{\text {ti},T}$. The translation-invariant BCS functional is studied in detail in [36], see also the review article [34]. In [36] it has been shown that there is a unique critical temperature ${T_{\text {c}}}\geqslant 0$ such that ${\mathcal {F}}^{\text {BCS}}_{\text {ti},T}$ has a minimizer with $\alpha \ne 0$ for $T < {T_{\text {c}}}$. The normal state in (1.12) with $h=0$ is the unique minimizer if $T\geqslant {T_{\text {c}}}$. Moreover, the critical temperature ${T_{\text {c}}}$ can be characterized by a linear criterion: It equals the unique temperature *T* such that the linear operator

$$\begin{aligned} K_{T} - V \end{aligned}$$

has zero as its lowest eigenvalue. Here $K_T = K_T(-\text {i}\nabla )$ with the symbol

1.14 $$\begin{aligned} K_T(p) {:}{=}\frac{p^2 - \mu }{\tanh \frac{p^2-\mu }{2T}}. \end{aligned}$$

The operator $$K_{T} - V $$ is understood to act on the space $$L_{\text {sym}}^2({\mathbb {R}}^3)$$ of reflection-symmetric square-integrable functions on $${\mathbb {R}}^3$$. To be precise, the results in [36] have been proven without the assumption $$\alpha (-x) = \alpha (x)$$ for a.e. $$x\in {\mathbb {R}}^3$$. In this case, the operator $$K_{T_{\text {c}}}- V$$ acts on functions in the Hilbert space $$L^2({\mathbb {R}}^3)$$ instead of $$L_{\text {sym}}^2({\mathbb {R}}^3)$$. The results in [36], however, equally hold in the case of symmetric Cooper pair wave functions. That is, they hold in the same way if *V* is reflection symmetric and if the translation-invariant BCS functional is minimized over functions $$\gamma (x)$$ and $$\alpha (x)$$ that are both assumed to be reflection symmetric.

We note that the function $K_T(p)$ satisfies the inequalities $K_T(p) \geqslant 2T$ for $\mu \geqslant 0$, as well as $K_T(p)\geqslant |\mu |/\tanh (|\mu |/(2T))$ for $\mu < 0$. Our assumptions on *V* guarantee that the essential spectrum of $K_T-V$ equals that of $K_T$, and hence an eigenvalue below 2*T* for $\mu \geqslant 0$ or below $|\mu |/\tanh (|\mu |/(2T))$ for $\mu < 0$ is necessarily isolated and of finite multiplicity. This, in particular, applies to an eigenvalue of $K_T - V$ at 0.

We are interested in the situation, where ${T_{\text {c}}}> 0$ and where the translation-invariant BCS functional has a unique minimizer with a radial Cooper pair wave function (s-wave Cooper pairs) for *T* close to ${T_{\text {c}}}$. The following assumptions guarantee that we are in such a situation. Part (b) should be compared to [17, Theorem 2.8].

#### Assumption 1.3

We assume that the interaction potential *V* is such that the following holds:

- We have $${T_{\text {c}}}>0$$.
- The lowest eigenvalue of $K_{{T_{\text {c}}}} - V$ is simple.

As has been shown in [36, Theorem 3], our first assumption is satisfied if $V \geqslant 0$ does not vanish identically. Throughout this paper we denote by $\alpha _*$ the unique solution to the equation

1.15 $$\begin{aligned} K_{T_{\text {c}}}\alpha _* = V\alpha _*. \end{aligned}$$

Since *V* is radial we know that the same is true for $\alpha _*$. Without loss of generality we will assume that $\alpha _*$ is real-valued and satisfies $\Vert \alpha _*\Vert _{L^2({\mathbb {R}}^3)} = 1$. If we write the above equation as $\alpha _* = K_{{T_{\text {c}}}}^{-1} V\alpha _*$, we see that $V\in L^\infty ({\mathbb {R}}^3)$ implies $\alpha _*\in H^2({\mathbb {R}}^3)$. Moreover, we know from [23, Proposition 2] that

1.16 $$\begin{aligned} \int _{{\mathbb {R}}^3} \text {d}x \; \bigl [ |x^\nu \alpha _*(x)|^2 + |x^\nu \nabla \alpha _*(x)|^2 \bigr ] < \infty \end{aligned}$$

holds for $$\nu \in {\mathbb {N}}_0^3$$.

### The Ginzburg–Landau functional

We say that a function $\Psi$ on $Q_h$ is *gauge-periodic* if the magnetic translations of the form

1.17 $$\begin{aligned} T_h(\lambda )\Psi (X)&{:}{=}\, \text {e}^{\text {i}{{\textbf {B}}}\cdot (\lambda \wedge X)} \; \Psi (X + \lambda ),&\lambda&\in \Lambda _h, \end{aligned}$$

leave $\Psi$ invariant. We highlight that $T(\lambda )$ in (1.3) equals $T_h(\lambda )$ provided we replace ${{\textbf {B}}}$ by $2{{\textbf {B}}}$. Let $\Lambda _0, \Lambda _2, \Lambda _3 >0$, $\Lambda _1, D \in {\mathbb {R}}$, and let $\Psi$ be a gauge-periodic function in the case $h=1$. The Ginzburg–Landau (GL) functional is defined by

1.18 $$\begin{aligned} {\mathcal {E}}^{\text {GL}}_{D}(\Psi )&{:}{=}\frac{1}{|Q|} \int _{Q} \text {d}X \; \bigl \{ \Lambda _0 \; |(-\text {i}\nabla + 2{{\textbf {A}}}(X))\Psi (X)|^2 + \Lambda _1 \, W(X)\, |\Psi (X)|^2 \nonumber \\&\quad - D \, \Lambda _2\, |\Psi (X)|^2 + \Lambda _3\,|\Psi (X)|^4\bigr \}. \end{aligned}$$

We highlight the factor 2 in front of the magnetic vector potential in (1.18). Its appearance is due to the fact that $\Psi$ describes the center-of-mass motion of Cooper pairs carrying twice the charge of a single fermion.

The Ginzburg–Landau energy is defined by

$$\begin{aligned} E^{\text {GL}}(D) {:}{=}\inf \bigl \{ {\mathcal {E}}^{\text {GL}}_{D}(\Psi ) : \Psi \in H_{\text {mag}}^1(Q)\bigr \}. \end{aligned}$$

We also define the critical parameter

1.19 $$\begin{aligned} {D_{\text {c}}}&{:}{=}\frac{1}{\Lambda _2} \inf {{\,\textrm{spec}\,}}_{L_{\text {mag}}^2(Q)} \bigl (\Lambda _0 \, (-\text {i}\nabla + {{\textbf {A}}})^2 + \Lambda _1 \, W\bigr ). \end{aligned}$$

As has been shown in [24, Lemma 2.5], we have $E^{\text {GL}}(D)< 0$ if $D > {D_{\text {c}}}$ and $E^{\text {GL}}(D)=0$ if $D \leqslant {D_{\text {c}}}$.

In our analysis we encounter the Ginzburg–Landau functional in an *h*-dependent version, where $Q, {{\textbf {A}}}, W$, and *D* in (1.18) are replaced by $Q_h, {{\textbf {A}}}_h, W_h$, and $h^2 D$, respectively. If we denote this functional by ${\mathcal {E}}^{\text {GL}}_{D, h}(\Psi )$ we have

$$\begin{aligned} \inf \bigl \{ {\mathcal {E}}^{\text {GL}}_{D, h}(\Psi ): \Psi \in H_{\text {mag}}^1(Q_h)\bigr \} = h^4 E^{\text {GL}}(D), \end{aligned}$$

which follows by scaling. More precisely, for given $\psi$ the function

1.20 $$\begin{aligned} \Psi (X)&{:}{=}h \; \psi (h \, X),&X\in {\mathbb {R}}^3, \end{aligned}$$

obeys

1.21 $$\begin{aligned} {\mathcal {E}}^{\text {GL}}_{D, h}(\Psi ) = h^4 {\mathcal {E}}^{\text {GL}}_{D}(\psi ). \end{aligned}$$

### Main results

Our first main result concerns an asymptotic expansion of the BCS free energy in the small parameter $h>0$. The precise statement is captured in the following theorem.

#### Theorem 1

Let Assumptions 1.1 and 1.3 hold, let $D \in {\mathbb {R}}$, and let the coefficients $\Lambda _0, \Lambda _1, \Lambda _2$, and $\Lambda _3$ be given by (3.20)–(3.23) below. Then there are constants $C>0$ and $h_0 >0$ such that for all $0 < h \leqslant h_0$, we have

1.22 $$\begin{aligned} F^{\text {BCS}}(h,\, {T_{\text {c}}}(1 - Dh^2)) = h^4 \; \bigl ( E^{\text {GL}}(D)+ R \bigr ), \end{aligned}$$

with *R* satisfying the estimate1.23Moreover, for any approximate minimizer $\Gamma$ of ${\mathcal {F}}^{\text {BCS}}_{h, T}$ at $T = {T_{\text {c}}}(1 - Dh^2)$ in the sense that

1.24 $$\begin{aligned} {\mathcal {F}}^{\text {BCS}}_{h, T}(\Gamma ) - {\mathcal {F}}^{\text {BCS}}_{h, T}(\Gamma _0) \leqslant h^4 \bigl ( E^{\text {GL}}(D)+ \rho \bigr ) \end{aligned}$$

holds for some $\rho \geqslant 0$, we have the decomposition

1.25 $$\begin{aligned} \alpha (X, r ) = \alpha _*(r) \Psi (X) + \sigma (X,r) \end{aligned}$$

for the Cooper pair wave function $\alpha = \Gamma _{12}$. Here, $\sigma$ satisfies1.26$\alpha _*$ is the normalized zero energy eigenstate of $K_{{T_{\text {c}}}}-V$, and the function $\Psi$ obeys

1.27 $$\begin{aligned} {\mathcal {E}}^{\text {GL}}_{D, h}(\Psi ) \leqslant h^4 \left( E^{\text {GL}}(D)+ \rho + {\mathcal {R}}\right) . \end{aligned}$$

Our second main result is a statement about the dependence of the critical temperature of the BCS functional on $h>0$ and on the external fields.

#### Theorem 2

Let Assumptions 1.1 and 1.3 hold. Then there are constants $C>0$ and $h_0 >0$ such that for all $0 < h \leqslant h_0$ the following holds:

- Let $0< T_0 < {T_{\text {c}}}$. If the temperature *T* satisfies 1.28 with ${D_{\text {c}}}$ in (1.19), then we have
  $$\begin{aligned} F^{\text {BCS}}(h,T) < 0. \end{aligned}$$
- If the temperature *T* satisfies
  1.29 $$\begin{aligned} T \geqslant {T_{\text {c}}}\, ( 1 - h^2 \, ( {D_{\text {c}}}- {\mathcal {R}}) ) \end{aligned}$$
  with ${D_{\text {c}}}$ in (1.19) and ${\mathcal {R}}$ in (1.23), then we have
  $$\begin{aligned} {\mathcal {F}}^{\text {BCS}}_{h, T}(\Gamma ) - {\mathcal {F}}^{\text {BCS}}_{h, T}(\Gamma _0) > 0 \end{aligned}$$
  unless $$\Gamma = \Gamma _0$$.

#### Remarks 1.4

- Theorem 1 and Theorem 2 extend similar results in [23] and [24] to the case of general external electric and magnetic fields. In these references the main restriction is that the vector potential is assumed to be periodic, that is, the corresponding magnetic field has vanishing flux through the faces of the unit cell $Q_h$, compare with the discussion in Sect. 1.2. Removing this restriction causes major mathematical difficulties already for the constant magnetic field because its vector potential cannot be treated as a perturbation of the Laplacian. More precisely, it was possible in [23, 24] to work with a priori bounds for low-energy states that do not involve the external magnetic field. As noticed in the discussion below Remark 6 in [26], this is not possible if the magnetic field has nonzero flux through the faces of the unit cell. To prove a priori bounds that involve a constant magnetic field one has to deal with the fact that the components of the magnetic momentum operator do not commute, which leads to significant technical difficulties. These difficulties have been overcome in [15], which allowed us to extend the results [23, 24] to the case of a system in a constant magnetic field. Our proof of Theorem 1 and Theorem 2 uses these a priori bounds, and should therefore be interpreted as an extension of the methods in [15] to the case of general external electric and magnetic fields. The main technical novelty of this article is a further development of the phase approximation method, which has been pioneered in the framework of BCS theory for the case of the constant magnetic field in [26] and [15]. It allows us to compute the BCS free energy of a class of trial states (Gibbs states) in a controlled way, and is the key new ingredient for our proof of upper and lower bounds for the BCS free energy in the presence of general external fields.
- When we compare our result in Theorem 1 to the main Theorem in [23], we notice the following differences: (1) We use microscopic coordinates while macroscopic coordinates are used in [23, 24], see the discussion above [23, Eq. (1.4)]. (2) Our free energy is normalized by a large volume factor, see (1.9) and (1.10). This is not the case in [23, 24]. Accordingly, the GL energy appears on the order $h^4$ in our setting and on the order *h* in the setting in [23]. (3) The leading order of the Cooper pair wave function in [23, Theorem 1] is of the form
  1.30 $$\begin{aligned} \frac{1}{2} \alpha _*(x-y) (\Psi (x) + \Psi (y)). \end{aligned}$$
  This should be compared to (1.25), where relative and center-of-mass coordinates are used. When we use the a priori bound for $\Vert \nabla \Psi \Vert _2$ below Eq. (5.61) in [23], we see that this decomposition equals that in (1.25) to leading order in *h*.
- The Ginzburg–Landau energy appears at the order $h^4$. This needs to be compared to the energy of the normal state, which is of order 1 in *h*. To understand the order of the GL energy we need to realize that each factor of $\Psi$ in ${\mathcal {E}}^{\text {GL}}_{D, h}$ defined below (1.19) carries a factor *h*. This follows from the scaling in (1.20) and the fact that the GL energy is normalized by the volume factor $|Q_h|^{-1}$. Moreover, every magnetic momentum operator carries a factor *h* because $\Psi$ varies on the length scale $h^{-1}$ and the electric potential carries a factor $h^2$. In combination, these considerations explain the size of all terms in the GL functional. It is worth noting that the prefactor $-h^2 D$ in front of the quadratic term without external fields equals $(T-T_{\text {c}})/T_{\text {c}}$.
- The size of the remainder in (1.26) should be compared to the $L^2$-norm per unit volume of the leading order part of the Cooper pair wave function in (1.25), which satisfies
  $$\begin{aligned} \frac{1}{| Q_h |} \int _{Q_h} \text {d}X \int _{{\mathbb {R}}^3} \text {d}r \; | \alpha _*(r) \Psi (X) |^2 \sim h^2 \end{aligned}$$
  if $D>0$. We highlight that $\alpha _*$ varies on the microscopic length scale 1 and that $\Psi$ captures the effects of the external fields on the macroscopic length scale $h^{-1}$.
- Our bounds show that *D* in Theorem 1 can be chosen as a function of *h* as long as $|D| \leqslant D_0$ holds for some constant $D_0>0$.
- The upper bound for the error in (1.23) is worse than the corresponding bound in [15] by the factor $h^{-1}$. It is of the same size as the comparable error term in [23, Theorem 1].
- Theorem 2 gives bounds on the temperature regions where superconductivity is present or absent. The interpretation of the theorem is that the critical temperature of the full model satisfies
  $$\begin{aligned} {T_{\text {c}}}(h) = {T_{\text {c}}}\left( 1 - D_{\text {c}} h^2 \right) + o(h^2), \end{aligned}$$
  with the critical temperature ${T_{\text {c}}}$ of the translation-invariant problem. The coefficient $D_{\text {c}}$ is determined by linearized Ginzburg–Landau theory, see (1.17). The above equation allows us to compute the upper critical field $B_{\text {c}2}$, above which superconductivity is absent. It also allows to to compute the derivative of $B_{\text {c}2}$ with respect to *T* at ${T_{\text {c}}}$, see [26, Appendix A].
- We expect that the assumption $0< T_0 < {T_{\text {c}}}$ in part (a) of Theorem 2, which also appeared in [15, 26], is only of technical nature. We need it because our trial state analysis breaks down as *T* approaches zero. We note that there is no such restriction in part (b) of Theorem 2 or in Theorem 1.

### Organization of the paper and strategy of proof

For the convenience of the reader we give here a short summary of the organization of the paper and the proof of our two main results.

In Sect. 1.8 we provide a brief non-rigorous computation that shows from which terms in the BCS functional the different terms in the GL functional arise. Afterwards we complete in Sect. 2 the introduction of our mathematical setup. That is, we collect useful properties of the trace per unit volume and introduce the relevant spaces of gauge-periodic functions.

In Sect. 3 we collect the results of our trial state analysis. We introduce a class of Gibbs states with Cooper pair wave functions that admit a product structure of the form $\alpha _*(r) \Psi (X)$ to leading order in *h*. Here, $\alpha _*$ is the ground state wave function in (1.15) and $\Psi$ is a gauge-periodic function. We state and motivate several results concerning the Cooper pair wave function and the BCS energy of these states. Afterwards we use these statements to provide the proof of the upper bound for the BCS free energy in (1.22) as well as the proof of Theorem 2 (a). It is important to note that these results are needed again in Sect. 6, where we give the proof of the lower bound on the BCS free energy in (1.22) and the proof of Theorem 2 (b).

In Sect. 4 we provide the proofs of the results in Sect. 3 concerning the Cooper pair wave function and the BCS free energy of our trial states. It is the main part of our article and contains the main technical novelties. Our approach is based on an application of the phase approximation method for general magnetic fields to our nonlinear setting. The phase approximation is a well-known tool in the physics literature, see, e.g., [37], and has also been used in the mathematical literature to study spectral properties of Schrödinger operators involving a magnetic field, for instance in [9, 42]. In the case of a constant magnetic field, this method has been pioneered within the framework of linearized BCS theory in [26] and for the full nonlinear model in [15]. An application of the phase approximation method to the case of a magnetic field with zero flux through the unit cell is contained in unpublished notes by Frank, Geisinger, Hainzl, and Tzaneteas [18]. The main technical novelty in Sect. 4 is a further development of the phase approximation method for general external fields in our nonlinear setting. This allows us to compute the BCS free energy of a class of trial states (Gibbs states) in a controlled way, which is the key new ingredient for the proof of upper and lower bounds for the BCS free energy in the presence of general external fields. Our approach should also be compared to the trial state analysis in [23, 24], where a semi-classical expansion is used to treat magnetic fields with zero flux through the unit cell. We highlight that the trial state analysis for general external fields requires considerably more effort than the one for a constant magnetic field in [15]. This is also reflected in the length of the proofs.

In Sect. 5, we prove a priori estimates for BCS states, whose BCS free energy is smaller than or equal to that of the normal state $\Gamma _0$ in (1.12) (low-energy states). More precisely, we show that the Cooper pair wave function of any such state is, to leading order as $h \rightarrow 0$, given by $\alpha _*(r) \Psi (X)$ with $\alpha _*$ in (1.15) and with a gauge-periodic function $\Psi$. The proof of the same statement in the case of a constant magnetic field has been the main novelty in [15]. To treat the case of general external fields, we perturbatively remove the periodic vector potential *A* and the electric potential *W*. This allows us to reduce the problem to the case of a constant magnetic field treated in [15]. Similar a priori estimates for the case of magnetic fields with zero flux through the unit cell had been proved for the first time in [23].

The proofs of the lower bound on (1.22) and of Theorem 2 (b), which go along the same lines as those presented in [15, 23, 24], are given in Sect. 6. They complete the proofs of Theorem 1 and 2. The main idea is to use the a priori estimates in Sect. 5 to replace a general low-energy state in the BCS functional by a Gibbs state, whose Cooper pair wave function has the same leading order behavior for small *h*, in a controlled way. This, in particluar, allows us to estimate the BCS energy of a general low-energy state in terms of that of a Gibbs state, which has been computed in Sects. 3 and 4. Because of the considerable overlap in content with the related section in [15], we shortened the proofs in this section to a minimal length.

Throughout the paper, *c* and *C* denote generic positive constants that change from line to line. We allow them to depend on the various fixed quantities like $h_0$, $D_0$, $\mu$, ${T_{\text {c}}}$, *V*, *A*, *W*, $\alpha _*$, etc. Further dependencies are highlighted.

### Heuristic computation of the terms in the Ginzburg–Landau functional

In the following we present a brief and non-rigorous computation of the BCS energy of the trial state that we use in the proof of the upper bound for the BCS free energy in Sect. 3. The goal is to show from which terms in the BCS functional the different terms in the Ginzburg–Landau functional arise. A more detailed and more precise discussion of these issues can be found in Sect. 3.

Our trial state (a Gibbs state) is defined by

1.31 $$\begin{aligned} \begin{pmatrix} \gamma _{\Delta } &{} \alpha _{\Delta } \\ \overline{\alpha _{\Delta }} &{} 1 - \overline{\gamma _{\Delta }} \end{pmatrix} = \Gamma _{\Delta } = \frac{1}{1+\text {e}^{\beta H_{\Delta }}}. \end{aligned}$$

Here, $\beta ^{-1} = T = {T_{\text {c}}}(1 - Dh^2)$ with $D>0$ and the Hamiltonian is given by

$$\begin{aligned} H_{\Delta } = \begin{pmatrix} (-\text {i} \nabla + {{\textbf {A}}}_h )^2 - \mu &{} \Delta \\ {\overline{\Delta }} &{} -\overline{(-\text {i} \nabla + {{\textbf {A}}}_h )^2} + \mu \end{pmatrix}, \end{aligned}$$

where the operator $\Delta$ is defined via its integral kernel

$$\begin{aligned} \Delta (x,y) = -2\, V \alpha _* (x-y)\, \Psi _h \left( \frac{x+y}{2} \right) . \end{aligned}$$

The function $\Psi _h(X) = h \psi (hX)$ is chosen such that $\psi$ is a minimizer of the Ginzburg–Landau functional in (1.18). We therefore have

$$\begin{aligned} \frac{1}{| Q_h|} \int _{Q_h} \text {d}X \ | \Psi _h(X) |^2 \sim h^2 \end{aligned}$$

as well as

1.32 $$\begin{aligned} {{\,\textrm{Tr}\,}}[ \Delta ^* \Delta ] = \frac{4}{| Q_h|} \int _{Q_h} \text {d}X \; | \Psi _h(X) |^2 \int _{{\mathbb {R}}^3} \text {d}r \; | V(r) \alpha _*(r) |^2 \sim h^2. \end{aligned}$$

Here $r=x-y$ and $X=(x+y)/2$ denote relative and center-of-mass coordinates. The operator $\Delta$ in (1.31) is therefore a small perturbation if $0 < h \ll 1$.

The BCS free energy of the trial state $\Gamma _{\Delta }$ is given by

$$\begin{aligned} {\mathcal {F}}^{\text {BCS}}_{h, T}(\Gamma _{\Delta }) - {\mathcal {F}}^{\text {BCS}}_{h, T}(\Gamma _0)&= \frac{1}{2} {{\,\textrm{Tr}\,}}\left[ H_0 (\Gamma _{\Delta } - \Gamma _0) \right] - T S(\Gamma ) + T S(\Gamma _0) \\&\quad - \frac{1}{| Q_h |} \int _{Q_h} \text {d}X \int _{{\mathbb {R}}^3} \text {d}r \; V(r) | \alpha _{\Delta }(X,r) |^2, \end{aligned}$$

where $\Gamma _0$ denotes the normal state in (1.12). Applications of

$$\begin{aligned} {{\,\textrm{Tr}\,}}\left[ H_0 (\Gamma _{\Delta } - \Gamma _0) \right] = {{\,\textrm{Tr}\,}}\left[ H_{\Delta } \Gamma _{\Delta } - H_0 \Gamma _0 \right] - {{\,\textrm{Tr}\,}}[ (H_{\Delta } - H_0 ) \Gamma _{\Delta } ], \end{aligned}$$

and [23, Eqs. (4.3$-$4.5)], allow us to rewrite this formula as

1.33 $$\begin{aligned} {\mathcal {F}}^{\text {BCS}}_{h, T}(\Gamma _{\Delta }) - {\mathcal {F}}^{\text {BCS}}_{h, T}(\Gamma _0) =&-\frac{1}{2 \beta }{{\,\textrm{Tr}\,}}_0 \bigl [ \ln \bigl ( 1+\exp \bigr ( - \beta H_\Delta \bigr )\bigr ) - \ln \bigl ( 1+\exp \bigl ( -\beta H_0 \bigr )\bigr )\bigr ] \nonumber \\&+ \frac{\langle \alpha _*, V\alpha _*\rangle _{L^2({\mathbb {R}}^3)}}{|Q_h|} \int _{Q_h} \text {d}X \ |\Psi _h(X)|^2 \nonumber \\&- \frac{1}{|Q_h|} \int _{Q_h} \text {d}X\int _{{\mathbb {R}}^3} \text {d}r\; V(r) \, \bigl |\alpha (X,r) - \alpha _*(r) \Psi _h(X)\bigr |^2. \end{aligned}$$

Here $${{\,\textrm{Tr}\,}}_0[A] = {{\,\textrm{Tr}\,}}[PAP] + {{\,\textrm{Tr}\,}}[QAQ]$$ with

$$\begin{aligned} P = \begin{pmatrix} 1 &{} 0 \\ 0 &{} 0 \end{pmatrix} \end{aligned}$$

and $$Q = 1 - P$$.

To identify the terms in the GL functional, we need to expand the terms in (1.33) in powers of *h*. To that end, we first expand them up to fourth order in powers of $\Delta$ because the Ginzburg–Landau functional is a fourth order polynomial in $\Psi _h$. Afterwards, we expand the resulting terms in powers of *h*, that is, we use that the external fields $W_h(x) = h^2 W(hx)$ and ${{\textbf {A}}}_h(x) = h {{\textbf {A}}}(hx)$ as well as the temperature $T = {T_{\text {c}}}(1 - Dh^2)$ with $D>0$ depend on *h*. It turns out that the last term on the right side of (1.33) is of the order $o(h^4)$. Since the GL energy appears on the order $h^4$ it does not contribute to it. In our trial state analysis in Sects. 3 and 4 we show that there is a linear operator $L_{T,{{\textbf {A}}},W}$ and a cubic map $N_{T,{{\textbf {A}}},W}(\Delta )$ such that

1.34 $$\begin{aligned} -\frac{1}{2\beta }{{\,\textrm{Tr}\,}}_0 \bigl [ \ln \bigl ( 1+\exp \bigl ( -&\beta H_\Delta \bigr )\bigr ) - \ln \bigl ( 1+\exp \bigl ( -\beta H_0 \bigr )\bigr )\bigr ] \nonumber \\&\quad = -\frac{1}{4} \langle \Delta , L_{T,{{\textbf {A}}},W} \Delta \rangle + \frac{1}{8} \langle \Delta , N_{T,{{\textbf {A}}},W}(\Delta ) \rangle + o(h^4) \end{aligned}$$

holds. In combination with the first term in the second line of (1.33), the quadratic terms in (1.34) contain the quadratic terms in the Ginzburg–Landau functional:

$$\begin{aligned}&-\frac{1}{4} \langle \Delta , L_{T,{{\textbf {A}}},W} \Delta \rangle + \frac{\langle \alpha _*, V\alpha _*\rangle _{L^2({\mathbb {R}}^3)}}{|Q_h|} \int _{Q_h} \text {d}X \; |\Psi _h(X)|^2 \\&= \frac{1}{|Q_h|} \int _{Q_h} \text {d}X \; \bigl \{ \Lambda _0 \; |(-\text {i}\nabla + 2{{\textbf {A}}}_h)\Psi _h(X)|^2 + \Lambda _1 \, W_h(X)\, |\Psi _h(X)|^2 - Dh^2 \, \Lambda _2\, |\Psi _h(X)|^2 \bigr \} \\&\quad + o(h^4) \end{aligned}$$

with $\Lambda _0, \Lambda _1,$ and $\Lambda _2$ defined in (3.20)-(3.22). From the quartic term in (1.34) we will extract the quartic term in the GL functional:

$$\begin{aligned} \frac{1}{8} \langle \Delta , N_{T,{{\textbf {A}}},W}(\Delta ) \rangle = \frac{\Lambda _3}{|Q_h|} \int _{Q_h} \text {d}X \; |\Psi _h(X)|^4 + o(h^4). \end{aligned}$$

The coefficient $\Lambda _3$ is defined in (3.23). Accordingly,

$$\begin{aligned} {\mathcal {F}}^{\text {BCS}}_{h, T}(\Gamma _{\Delta }) - {\mathcal {F}}^{\text {BCS}}_{h, T}(\Gamma _0) = h^4 \left( E^{\text {GL}}(D)+ o(1) \right) , \end{aligned}$$

where we used ${\mathcal {E}}^{\text {GL}}_{D, h}(\Psi _h) = h^4 E^{\text {GL}}(D)$ in the last step.

## Preliminaries

### Schatten classes

The trace per unit volume in (1.9) gives rise to Schatten classes of periodic operators, whose norms play an important role in our proofs. In this section we recall several well-known facts about these norms.

For $1 \leqslant p < \infty$, the *p* $^{\text {th}}$ local von-Neumann–Schatten class ${\mathcal {S}}^p$ consists of all gauge-periodic operators *A* having finite *p*-norm, that is, $\Vert A\Vert _p^p {:}{=}{{\,\textrm{Tr}\,}}(|A|^p) <\infty$. The space of bounded gauge-periodic operators ${\mathcal {S}}^\infty$ is equipped with the usual operator norm. We note that the *p*-norm is not monotone decreasing in the index *p*. This should be compared to the usual Schatten norms, where such a property holds, see the discussion below [23, Eq. (3.9)].

We recall that the triangle inequality

$$\begin{aligned} \Vert A + B\Vert _p \leqslant \Vert A\Vert _p + \Vert B\Vert _p \end{aligned}$$

holds for operators in ${\mathcal {S}}^p$ for $1 \leqslant p \leqslant \infty$. We also have the generalized version of Hölder’s inequality

2.1 $$\begin{aligned} \Vert AB\Vert _r \leqslant \Vert A\Vert _p \Vert B\Vert _q, \end{aligned}$$

which holds for $1 \leqslant p,q,r \leqslant \infty$ with $\frac{1}{r} = \frac{1}{p} + \frac{1}{q}$. The familiar inequality

$$\begin{aligned} | {{\,\textrm{Tr}\,}}A | \leqslant \Vert A \Vert _1 \end{aligned}$$

also holds in the case of local Schatten norms.

The above inequalities can be deduced from their versions for the usual Schatten norms, see, e.g., [47], with the help of the magnetic Bloch–Floquet decomposition. We refer to [44, Section XIII.16] for an introduction to the Bloch–Floquet transformation and to [28] for a treatment of the magnetic case. To be more precise, a gauge-periodic operator *A* satisfies the unitary equivalence

$$\begin{aligned} A \cong \int ^{\oplus }_{[0,\sqrt{ 2 \pi }\, h]^3} \text {d} k \; A_{k}, \end{aligned}$$

which we use to write the trace per unit volume as

2.2 $$\begin{aligned} {{\,\textrm{Tr}\,}}A = \int _{[0,\sqrt{ 2 \pi }\, h]^3} \frac{\text {d}k}{(2\pi )^3} \; {{\,\textrm{Tr}\,}}_{L^2(Q_h)} A_{k}. \end{aligned}$$

Here, ${{\,\textrm{Tr}\,}}_{L^2(Q_h)}$ denotes the usual trace over $L^2(Q_h)$. When we use that $(AB)_k = A_k B_k$ holds for gauge-periodic operators *A* and *B*, the above mentioned inequalities for the trace per unit volume are implied by their usual versions.

### Gauge-periodic Sobolev spaces

In this section we introduce Banach spaces of gauge-periodic functions, which will be used to describe Cooper pair wave functions of BCS states.

When working with center-of-mass and relative coordinates (*X*, *r*) it is useful to define the magnetic momentum operators

2.3 $$\begin{aligned} \Pi _{{{\textbf {A}}}}&{:}{=}-\text {i}\nabla _X + 2 {{\textbf {A}}}(X),&\widetilde{\pi }_{{{\textbf {A}}}}&{:}{=}-\text {i}\nabla _r + \frac{1}{2} {{\textbf {A}}}(r). \end{aligned}$$

We will also use the notation

2.4 $$\begin{aligned} \Pi&{:}{=}-\text {i}\nabla _X + 2 {{\textbf {A}}}_{{{\textbf {B}}}}(X),&{\widetilde{\pi }}&{:}{=}-\text {i}\nabla _r + \frac{1}{2} {{\textbf {A}}}_{{\textbf {B}}}(r). \end{aligned}$$

If several coordinates appear in an equation we sometimes write $\Pi _X$ and ${\widetilde{\pi }}_r$ to highlight on which coordinate $\Pi$ and ${\widetilde{\pi }}$ are acting.

A function $\Psi \in L_\text {loc}^p({\mathbb {R}}^3)$ with $1 \leqslant p \leqslant \infty$ belongs to the space $L_{\text {mag}}^p(Q_h)$ provided $T_h(\lambda )\Psi = \Psi$ holds for all $\lambda \in \Lambda _h$ (with $T_h(\lambda )$ in (1.17)). We endow $L_{\text {mag}}^p(Q_h)$ with the usual *p*-norm per unit volume2.5if $1 \leqslant p < \infty$ and with the $L^{\infty }(Q_h)$-norm if $p=\infty$. When it does not lead to confusion we use the abbreviation $\Vert \Psi \Vert _p$.

Analogously, for $m\in {\mathbb {N}}_0$, we define the Sobolev spaces of gauge-periodic functions corresponding to the constant magnetic field as

2.6 $$\begin{aligned} H_{\text {mag}}^m(Q_h)&{:}{=}\bigl \{ \Psi \in L_{\text {mag}}^2(Q_h) : \Pi ^\nu \Psi \in L_{\text {mag}}^2(Q_h) \quad \forall \nu \in {\mathbb {N}}_0^3, |\nu |_1\leqslant m\bigr \}, \end{aligned}$$

where $|\nu |_1 {:}{=}\sum _{i=1}^3 \nu _i$ for $\nu \in {\mathbb {N}}_0^3$. It is a Hilbert space when endowed with the inner product

2.7 $$\begin{aligned} \langle \Phi , \Psi \rangle _{H_{\text {mag}}^m(Q_h)}&{:}{=}\sum _{|\nu |_1\leqslant m} h^{-2 - 2|\nu |_1} \; \langle \Pi ^\nu \Phi , \Pi ^\nu \Psi \rangle _{L_{\text {mag}}^2(Q_h)}. \end{aligned}$$

We note that if $\Psi$ is a gauge-periodic function then so is $\Pi ^\nu \Psi$, since the magnetic momentum operator $\Pi$ commutes with the magnetic translations $T_h(\lambda )$ in (1.17). Furthermore, $\Pi$ is a self-adjoint operator on $H_{\text {mag}}^1(Q_h)$.

The norms introduced in (2.5) and (2.7) display a scaling behavior with respect to *h*, which is motivated by the Ginzburg–Landau scaling in (1.20). More precisely, whenever $\psi \in L_{\text {mag}}^p(Q)$ and $\Psi (x) = h \psi (hx)$, then

2.8 $$\begin{aligned} \Vert \Psi \Vert _{L_{\text {mag}}^p(Q_h)} = h \, \Vert \psi \Vert _{L_{\text {mag}}^p(Q)} \end{aligned}$$

for every $1\leqslant p \leqslant \infty$. That is, $\Psi \sim h$ in any *p*-norm per unit volume.

The inner product in (2.7) is chosen such that

$$\begin{aligned} \Vert \Psi \Vert _{H_{\text {mag}}^m(Q_h)} = \Vert \psi \Vert _{H_{\text {mag}}^m(Q)} \end{aligned}$$

holds. This follows from (2.8) and the fact that $\Vert \Pi ^\nu \Psi \Vert _2^2$ scales as $h^{2 + 2|\nu |_1}$ for $\nu \in {\mathbb {N}}_0^3$. Such scaled norms have also been used in [15] but not in [23, 24].

For the sake of completeness, let us also mention the following magnetic Sobolev inequality. There is a constant $C>0$ such that for any $h>0$ and any $\Psi \in H_{\text {mag}}^1(Q_h)$, we have

2.9 $$\begin{aligned} \Vert \Psi \Vert _{L_{\text {mag}}^6(Q_h)}^2&\leqslant C \, h^{-2}\, \Vert \Pi \Psi \Vert _{L_{\text {mag}}^2(Q_h)}^2. \end{aligned}$$

The proof can be found in [15] below Eq. (2.7).

The Cooper pair wave function $\alpha$ of an admissible BCS state $\Gamma$ belongs to the Hilbert–Schmidt class ${\mathcal {S}}^2$ defined in Sect. 2.1, see the discussion below (1.9). The symmetry and the gauge-periodicity of the kernel of $\alpha$ in (1.7) can be reformlated as

2.10 $$\begin{aligned} \alpha (X,r)&= \text {e}^{\text {i}{{\textbf {B}}}\cdot (\lambda \wedge X)} \; \alpha (X+ \lambda , r), \quad \lambda \in \Lambda _h;&\alpha (X,r)&= \alpha (X, -r) \end{aligned}$$

in terms of center-of-mass and relative coordinates. In other words, $\alpha (X,r)$ is a gauge-periodic function of the center-of-mass coordinate $X \in {\mathbb {R}}^3$ and a reflection-symmetric function of the relative coordinate $r \in {\mathbb {R}}^3$. We make use of the unitary equivalence of ${\mathcal {S}}^2$ and the space

$$\begin{aligned} {L^2(Q_h \times {\mathbb {R}}_{{\text {s}}}^3)}{:}{=}L_{\text {mag}}^2(Q_h) \otimes L_{\text {sym}}^2({\mathbb {R}}^3), \end{aligned}$$

which consists of all square-integrable functions satisfying (2.10). We also define the normThe identity $\Vert \alpha \Vert _2 = \Vert \alpha \Vert _{{L^2(Q_h \times {\mathbb {R}}_{{\text {s}}}^3)}}$ follows from (2.10). In the following we therefore identify the scalar products $\langle \cdot , \cdot \rangle$ on ${L^2(Q_h \times {\mathbb {R}}_{{\text {s}}}^3)}$ and ${\mathcal {S}}^2$ with each other and we do not distinguish between operators in ${\mathcal {S}}^2$ and their kernels as this does not lead to confusion.

By $H^1(Q_h\times {\mathbb {R}}_{{\text {s}}}^3)$ we denote the Sobolev space of all functions $\alpha \in L^2(Q_h\times {\mathbb {R}}_{{\text {s}}}^3)$, which have finite $H^1$-norm defined by

2.11 $$\begin{aligned} \Vert \alpha \Vert _{H^1(Q_h\times {\mathbb {R}}_{{\text {s}}}^3)}^2&{:}{=}\Vert \alpha \Vert _2^2 + \Vert \Pi \alpha \Vert _2^2 + \Vert \widetilde{\pi }\alpha \Vert _2^2 \end{aligned}$$

with $\Pi$ and ${\widetilde{\pi }}$ in (2.4).

We highlight that the norm in (2.11) is equivalent to the two norms

2.12 $$\begin{aligned} {{\,\textrm{Tr}\,}}[\alpha \alpha ^*] + {{\,\textrm{Tr}\,}}[(-\text {i}\nabla + {{\textbf {A}}}_{{\textbf {B}}})\alpha \alpha ^* (-\text {i}\nabla + {{\textbf {A}}}_{{\textbf {B}}})] + {{\,\textrm{Tr}\,}}[(-\text {i}\nabla + {{\textbf {A}}}_{{\textbf {B}}}) \alpha ^* \alpha (-\text {i}\nabla + {{\textbf {A}}}_{{\textbf {B}}})] \end{aligned}$$

and

2.13 $$\begin{aligned} \Vert \alpha \Vert _2^2 + \Vert (-\text {i}\nabla + {{\textbf {A}}}_{{\textbf {B}}})\alpha \Vert _2^2 + \Vert \alpha (-\text {i}\nabla + {{\textbf {A}}}_{{\textbf {B}}})\Vert _2^2, \end{aligned}$$

compare also with the discussion below (1.9). We also note that the $H^m$-norm in (2.7) and the $H^1$-norm in (2.11) are equivalent to the norms that we obtain when ${{\textbf {A}}}_{\text {B}}$ is replaced by ${{\textbf {A}}}= {{\textbf {A}}}_{\text {B}} + A$ with a periodic vector potential $A \in L^{\infty }({\mathbb {R}}^3)$.

## Trial states and their BCS energy

In this section we introduce a class of trial states (Gibbs states), state several results concerning their Cooper pair wave function and their BCS free energy, and use these results to prove the upper bound on (1.22) as well as Theorem 2 (a). The trial states $\Gamma _{\Delta }$ are of the form stated in (1.31). In Proposition 3.2 we show that if $\Delta$ is given by $V \alpha _*(r) \Psi (X)$ with a gauge periodic function $\Psi$ that is small in an appropriate sense for small *h*, then $[\Gamma _{\Delta }]_{12} = \alpha _{\Delta } \approx \alpha _*(r) \Psi (X)$ to leading order in *h*. In Proposition 3.5 we prove a representation formula for the BCS functional that allows us to compute the BCS energy of the trial states $\Gamma _\Delta$. Finally, in Theorem 3.6 we extract the terms of the Ginzburg–Landau functional from the BCS free energy of $\Gamma _{\Delta }$. The proofs of these statements are given in Sect. 4. Our trial analysis should be viewed as further development of that in [15] for the constant magnetic field. The techniques we develop in Sects. 3 and 4 are based on gauge-invariant perturbation theory, which has been pioneered in the framework of linearized BCS theory for a constant external magnetic field in [26]. Our approach should also be compared to the trial state analysis in [23, 24], where a semi-classical expansion is used to treat magnetic fields with zero flux through the unit cell.

### The Gibbs states $\Gamma _\Delta$

For $\Psi \in L_{\text {mag}}^2(Q_h)$ we define the gap function $\Delta \in {L^2(Q_h \times {\mathbb {R}}_{{\text {s}}}^3)}$ by

3.1 $$\begin{aligned} \Delta (X,r) {:}{=}\Delta _\Psi (X, r)&{:}{=}-2 \; V\alpha _*(r) \Psi (X). \end{aligned}$$

We also introduce the one-particle Hamiltonian

3.2 $$\begin{aligned} {\mathfrak {h}}_{{{\textbf {A}}}, W}&{:}{=}\, {\mathfrak {h}}_{{\textbf {A}}}+ W {:}{=}(-\text {i}\nabla +{{\textbf {A}}}_h )^2 + W_h - \mu \end{aligned}$$

as well as

3.3 $$\begin{aligned} H_{\Delta }&{:}{=}H_0 + \delta {:}{=}\begin{pmatrix} {\mathfrak {h}}_{{{\textbf {A}}}, W} &{} 0 \\ 0 &{} -\overline{{\mathfrak {h}}_{{{\textbf {A}}}, W}} \end{pmatrix} + \begin{pmatrix} 0 &{} \Delta \\ \overline{\Delta }&{} 0 \end{pmatrix} = \begin{pmatrix} {\mathfrak {h}}_{{{\textbf {A}}}, W} &{} \Delta \\ \overline{\Delta }&{} -\overline{{\mathfrak {h}}_{{{\textbf {A}}}, W}} \end{pmatrix}. \end{aligned}$$

The Gibbs state at inverse temperature $\beta = T^{-1} >0$ is defined by

3.4 $$\begin{aligned} \begin{pmatrix} \gamma _\Delta &{} \alpha _\Delta \\ \overline{\alpha _\Delta } &{} 1 - \overline{\gamma _\Delta }\end{pmatrix} = \Gamma _\Delta {:}{=}\frac{1}{1 + \text {e}^{\beta H_\Delta }}. \end{aligned}$$

We highlight that the choice $\Delta =0$ yields the normal state $\Gamma _0$ in (1.12). In our proof of the upper bound for the free energy in (1.22) we will choose $\Psi$ as a minimizer of the Ginzburg–Landau functional in (1.18), which satisfies the scaling in (1.20). Since the $L^2({\mathbb {R}}^3)$-norm of $V\alpha _*$ is of the order 1, the local Hilbert–Schmidt norm of $\Delta$ is of the order *h* in this case. In the proof of the lower bound we have less information about the function $\Psi$. The related difficulties are discussed in Remark 3.3 below.

#### Lemma 3.1

(Admissibility of $\Gamma _\Delta$) Let Assumptions 1.1 and 1.3 hold. Then, for any $h>0$, any $T>0$, and any $\Psi \in H_{\text {mag}}^1(Q_h)$, the state $\Gamma _\Delta$ in (3.4) is admissible.

The choice of the states $\Gamma _\Delta$ is motivated by the following observation. Using standard variational arguments one can show that any minimizer $\Gamma$ of the BCS functional satisfies the nonlinear Bogolubov–de Gennes equation

3.5 $$\begin{aligned} \Gamma&= \frac{1}{1 + \text {e}^{\beta \, {\mathbb {H}}_{V\alpha }}},&{\mathbb {H}}_{V\alpha } = \begin{pmatrix} {\mathfrak {h}}_{{{\textbf {A}}}, W} &{} -2\, V\alpha \\ -2\, \overline{V\alpha } &{} -\overline{{\mathfrak {h}}_{{{\textbf {A}}}, W}}\end{pmatrix}. \end{aligned}$$

Here, $V\alpha$ is the operator given by the integral kernel $V(r)\alpha (X,r)$. Since we are interested in approximate minimizers of the BCS functional, we choose $\Gamma _{\Delta }$ as an approximate solution to the BdG-equation in (3.5). The next result shows that, as far as the leading order behavior of $\alpha _{\Delta }$ is concerned, this is indeed the case. It should be compared to (1.25).

#### Proposition 3.2

(Structure of $\alpha _\Delta$) Let Assumption 1.1 and 1.3(a) be satisfied and let $T_0>0$ be given. Then, there is a constant $h_0>0$ such that for any $0 < h \leqslant h_0$, any $T\geqslant T_0$, and any $\Psi \in H_{\text {mag}}^2(Q_h)$ the function $\alpha _\Delta$ in (3.4) with $\Delta \equiv \Delta _\Psi$ as in (3.1) has the decomposition

3.6 $$\begin{aligned} \alpha _\Delta (X,r)&= \Psi (X) \alpha _*(r) - \eta _0(\Delta )(X,r) - \eta _{\perp }(\Delta )(X,r). \end{aligned}$$

The remainder functions $\eta _0(\Delta )$ and $\eta _\perp (\Delta )$ have the following properties:

- The function $\eta _0$ satisfies the bound
  3.7 $$\begin{aligned} \Vert \eta _0\Vert _{H^1(Q_h \times {\mathbb {R}}_{{\text {s}}}^3)}^2&\leqslant C\; \bigl ( h^5 + h^2 \, |T - {T_{\text {c}}}|^2\bigr ) \; \bigl ( \Vert \Psi \Vert _{H_{\text {mag}}^1(Q_h)}^6 + \Vert \Psi \Vert _{H_{\text {mag}}^1(Q_h)}^2\bigr ). \end{aligned}$$
- The function $\eta _\perp$ satisfies the bound
  3.8 $$\begin{aligned} \Vert \eta _\perp \Vert _{{H^1(Q_h \times {\mathbb {R}}_{{\text {s}}}^3)}}^2 + \Vert |r|\eta _\perp \Vert _{{L^2(Q_h \times {\mathbb {R}}_{{\text {s}}}^3)}}^2&\leqslant C \; h^6 \; \Vert \Psi \Vert _{H_{\text {mag}}^2(Q_h)}^2. \end{aligned}$$
- The function $\eta _\perp$ has the explicit form
  $$\begin{aligned} \eta _\perp (X, r)&= \int _{{\mathbb {R}}^3} \text {d}Z \int _{{\mathbb {R}}^3} \text {d}s \; k_T(Z, r-s) \, V\alpha _*(s) \, \bigl [ \cos (Z\cdot \Pi ) - 1\bigr ] \Psi (X) \end{aligned}$$
  with $k_T(Z,r)$ defined in Sect. 4 below (4.108). Moreover, for any radial functions $f,g\in L^2({\mathbb {R}}^3)$ the operator
  $$\begin{aligned} \iiint _{{\mathbb {R}}^9} \text {d}Z \text {d}r \text {d}s \; f(r) \, k_T(Z, r-s) \, g(s) \, \bigl [ \cos (Z\cdot \Pi ) - 1\bigr ] \end{aligned}$$
  commutes with $\Pi ^2$. In particular, if *P* and *Q* are two spectral projections of $\Pi ^2$ with $P Q = 0$, then $\eta _\perp$ satisfies the orthogonality property
  3.9 $$\begin{aligned} \bigl \langle f(r) \, (P \Psi )(X), \, \eta _{\perp }(\Delta _{Q\Psi }) \bigr \rangle = 0. \end{aligned}$$

#### Remark 3.3

The statement of Proposition 3.2 should be read in two different ways, depending on whether we are interested in proving the upper or the lower bound for the BCS free energy. In the former case, the bound on $\Vert |r|\eta _\perp \Vert _{{L^2(Q_h \times {\mathbb {R}}_{{\text {s}}}^3)}}$ in part (b) and part (c) are irrelevant. The reason is that the gap function $\Delta \equiv \Delta _\Psi$ is defined with a minimizer $\Psi$ of the GL functional, whose $H_{\text {mag}}^2(Q_B)$-norm is uniformly bounded. In this case all remainder terms can be estimated using (3.7) and (3.8).

In contrast, in the proof of the lower bound for the BCS free energy in Sect. 6 we are forced to work with a trial state $\Gamma _{\Delta }$, whose gap function is defined in terms of a function $\Psi$ that is related to a low-energy state of the BCS functional. The properties of such a function are captured in Theorem 5.1 below. In this case we only have a bound on the $H_{\text {mag}}^1(Q_h)$-norm of $\Psi$ at our disposal. To obtain a function in $H_{\text {mag}}^2(Q_h)$, we introduce a regularized version of $\Psi$ as in [23, Section 6], [24, Section 6], [26, Section 7], and [15, Section 6] by $\Psi _\leqslant {:}{=}\mathbb {1}_{[0,\varepsilon ]}(\Pi ^2)\Psi$ for some $h^2 \ll \varepsilon \ll 1$, see Corollary 5.2. The $H_{\text {mag}}^2(Q_h)$-norm of $\Psi _\leqslant$ is not uniformly bounded in *h*, see (5.5) below. This causes a certain error term in the proof of the lower bound to be large, a priori. Part (b) and (c) of Proposition 3.2 are needed to overcome this problem. Since many details of the relevant proof in Sect. 6 have been omitted because they go along the same lines as those in [15, Section 6] we refer to [15, Remark 3.3] for more details.

### The BCS energy of the states $\Gamma _\Delta$

In this section we compute the BCS free energy of our trial states $\Gamma _\Delta$. The goal is to show that this energy minus the energy of the normal state $\Gamma _0$ is, to leading order as $h \rightarrow 0$, given by the Ginzburg–Landau energy of the function $\Psi$ appearing in the definition of $\Delta$. For a brief heuristic summary of these computations we refer to Sect. 1.8.

We start our discussion by introducing the operators $L_{T, {{\textbf {A}}}, W}$ and $N_{T, {{\textbf {A}}}, W}$ that naturally appear when the BCS energy of $\Gamma _{\Delta }$ is expanded in powers of the gap function $\Delta$, see (1.34). The Matsubara frequencies are given by

3.10 $$\begin{aligned} \omega _n&{:}{=}\,\pi (2n+1) T, \qquad n \in {\mathbb {Z}}. \end{aligned}$$

For a local Hilbert–Schmidt operator $\Delta$ with integral kernel $\Delta (x,y)$ satisfying (1.7) and $\Delta (x,y) = \Delta (y,x)$ we define the linear map $L_{T, {{\textbf {A}}}, W}$ by

3.11 $$\begin{aligned} L_{T, {{\textbf {A}}}, W}\Delta&{:}{=}-\frac{2}{\beta }\sum _{n\in {\mathbb {Z}}} \frac{1}{\text {i}\omega _n - {\mathfrak {h}}_{{{\textbf {A}}}, W}} \, \Delta \, \frac{1}{\text {i}\omega _n + \overline{{\mathfrak {h}}_{{{\textbf {A}}}, W}}}. \end{aligned}$$

The operator ${\mathfrak {h}}_{{{\textbf {A}}}, W}$ is defined in (3.2). In the parameter regime we are interested in, we obtain the quadratic terms in the Ginzburg–Landau functional from $\langle \Delta , L_{T, {{\textbf {A}}}, W}\Delta \rangle$. The spectral properties of the operator $L_{T, {{\textbf {A}}}, W}$ have been studied in great detail for $W=0$ and ${{\textbf {A}}}= {{\textbf {A}}}_{e_3}$ in [26]. This allows the authors to compute the BCS critical temperature shift caused by a small constant magnetic field within the framework of linearized BCS theory. That this prediction is accurate also if the nonlinear problem is considered has been shown in [15].

Moreover, the nonlinear (cubic) map $N_{T, {{\textbf {A}}}, W}$ is defined by

3.12 $$\begin{aligned} N_{T, {{\textbf {A}}}, W}(\Delta )&{:}{=}\frac{2}{\beta }\sum _{n\in {\mathbb {Z}}} \frac{1}{\text {i}\omega _n - {\mathfrak {h}}_{{{\textbf {A}}}, W}}\, \Delta \, \frac{1}{\text {i}\omega _n + \overline{{\mathfrak {h}}_{{{\textbf {A}}}, W}}} \, \overline{\Delta }\, \frac{1}{\text {i}\omega _n - {\mathfrak {h}}_{{{\textbf {A}}}, W}}\, \Delta \, \frac{1}{\text {i}\omega _n + \overline{{\mathfrak {h}}_{{{\textbf {A}}}, W}}}. \end{aligned}$$

The expression $\langle \Delta , N_{T, {{\textbf {A}}}, W}(\Delta )\rangle$ gives rise to the quartic term in the Ginzburg–Landau functional. The operator $N_{T, {{\textbf {A}}}, W}$ also appeared in [18] and in [15].

From the following lemma we know that $L_{T,{{\textbf {A}}},W} \Delta$ and $N_{T, {{\textbf {A}}}, W}(\Delta )$ are in ${L^2(Q_h \times {\mathbb {R}}_{{\text {s}}}^3)}$ provided $\Delta$ satisfies the symmetry relations in (2.10) and some mild regularity assumptions.

#### Lemma 3.4

The map $L_{T,{{\textbf {A}}},W}$ is a bounded linear operator on ${L^2(Q_h \times {\mathbb {R}}_{{\text {s}}}^3)}$. Assume that the integral kernel $\Delta \in L^{2}(Q_h \times {\mathbb {R}}_{\text {s}}^3)$ defines a bounded operator on $L^2({\mathbb {R}}^3)$. Then we have $N_{T, {{\textbf {A}}}, W}(\Delta ) \in {L^2(Q_h \times {\mathbb {R}}_{{\text {s}}}^3)}$.

The following representation formula for the BCS functional is the starting point of our proofs of Theorems 1 and 2. It will be used to prove upper and lower bounds, and is therefore formulated for general BCS states and not only for our trial states. The proof of Proposition 3.5 can be found in [15, Proposition 3.4].

#### Proposition 3.5

(Representation formula for the BCS functional) Let $\Gamma$ be an admissible state. For any $h>0$, let $\Psi \in H_{\text {mag}}^1(Q_h)$ and let $\Delta \equiv \Delta _\Psi$ be as in (3.1). For $T>0$ and if , there is an operator ${\mathcal {R}}_{T, {{\textbf {A}}}, W}^{(1)}(\Delta )\in {\mathcal {S}}^1$ such that3.13where

3.14 $$\begin{aligned} {\mathcal {H}}_0(\Gamma , \Gamma _\Delta ) {:}{=}{{\,\textrm{Tr}\,}}_0\bigl [ \Gamma (\ln \Gamma - \ln \Gamma _\Delta ) + (1 - \Gamma )(\ln (1-\Gamma ) - \ln (1 - \Gamma _\Delta ))\bigr ] \end{aligned}$$

denotes the relative entropy of $\Gamma$ with respect to $\Gamma _\Delta$. Moreover, ${\mathcal {R}}_{T, {{\textbf {A}}}, W}^{(1)}(\Delta )$ obeys the estimate

$$\begin{aligned} \Vert {\mathcal {R}}_{T, {{\textbf {A}}}, W}^{(1)}(\Delta ) \Vert _1 \leqslant C \; T^{-5} \; h^6 \; \Vert \Psi \Vert _{H_{\text {mag}}^1(Q_h)}^6. \end{aligned}$$

The definition (3.14) of the relative entropy uses a weaker form of trace called ${{\,\textrm{Tr}\,}}_0$, which is defined as follows. We call a gauge-periodic operator ${\mathcal {R}}$ acting on $L^2({\mathbb {R}}^3)\oplus L^2({\mathbb {R}}^3)$ weakly locally trace class if $P_0 {\mathcal {R}}P_0$ and $Q_0{\mathcal {R}}Q_0$ are locally trace class, where

3.15 $$\begin{aligned} P_0 = \begin{pmatrix} 1 &{} 0 \\ 0 &{} 0 \end{pmatrix} \end{aligned}$$

and $Q_0 = 1-P_0$. For such operators the weak trace per unit volume is defined by

3.16 $$\begin{aligned} {{\,\textrm{Tr}\,}}_0 ({\mathcal {R}}){:}{=}{{\,\textrm{Tr}\,}}\bigl ( P_0{\mathcal {R}}P_0 + Q_0 {\mathcal {R}}Q_0\bigr ). \end{aligned}$$

If an operator ${\mathcal {R}}$ is locally trace class then it is also weakly locally trace class but the converse statement does not hold in general. The converse is true, however, if ${\mathcal {R}}\geqslant 0$. We highlight that if ${\mathcal {R}}$ is locally trace class then the weak trace per unit volume and the trace per unit volume coincide. Before their appearance in the context of BCS theory in [15, 23, 24], weak traces of the above kind appeared in [22, 35].

Let us have a closer look at the right side of (3.13). From the terms in the first line we will extract the Ginzburg–Landau functional, see Theorem 3.6 below. The terms in the second and third line contribute to the remainder. The term in the second line is small in absolute value, but the techniques used to bound the third line differ for upper and lower bounds. This is responsible for the different qualities of the upper and lower bounds in Theorems 1 and 2, see (1.23). For an upper bound we choose $\Gamma {:}{=}\Gamma _\Delta$. Hence ${\mathcal {H}}_0(\Gamma _\Delta , \Gamma _\Delta )=0$ and the last term in (3.13) can be estimated with the help of Proposition 3.2. To obtain a lower bound, the third line needs to be bounded from below using the lower bound for the relative entropy in [15, Lemma 6.1], which appeared for the first time in [23, Lemma 5].

Before we state the next result, we introduce the function

3.17 $$\begin{aligned} \widehat{V\alpha _*}(p) {:}{=}\int _{{\mathbb {R}}^3} \text {d}x\; \text {e}^{-\text {i}p\cdot x} \, V(x)\alpha _*(x), \end{aligned}$$

which also fixes our convention of the Fourier transform.

#### Theorem 3.6

(Calculation of the GL energy) Let Assumptions 1.1 and 1.3(a) hold and let $D\in {\mathbb {R}}$ be given. Then, there is a constant $h_0>0$ such that for any $0 < h \leqslant h_0$, any $\Psi \in H_{\text {mag}}^2(Q_h)$, $\Delta \equiv \Delta _\Psi$ as in (3.1), and $T = {T_{\text {c}}}(1 - Dh^2)$, we have

3.18 $$\begin{aligned} - \frac{1}{4} \langle \Delta , L_{T, {{\textbf {A}}}, W} \Delta \rangle + \frac{1}{8} \langle \Delta , N_{T, {{\textbf {A}}}, W} (\Delta )\rangle + \Vert \Psi \Vert _{L_{\text {mag}}^2(Q_h)}^2 \;&\langle \alpha _*, V\alpha _*\rangle _{L^2({\mathbb {R}}^3)} \nonumber \\&\quad = {\mathcal {E}}^{\text {GL}}_{D, h}(\Psi ) + R(h). \end{aligned}$$

Here,

$$\begin{aligned} |R(h)|\leqslant C \, \bigl [ h^5 \, \Vert \Psi \Vert _{H_{\text {mag}}^1(Q_h)}^2 + h^6 \, \Vert \Psi \Vert _{H_{\text {mag}}^2(Q_h)}^2 \bigr ] \, \bigl [ 1 + \Vert \Psi \Vert _{H_{\text {mag}}^1(Q_h)}^2 \bigr ] \end{aligned}$$

and with the functions

3.19 $$\begin{aligned} g_1(x)&{:}{=}\frac{\tanh (x/2)}{x^2} - \frac{1}{2x}\frac{1}{\cosh ^2(x/2)},&g_2(x)&{:}{=}\frac{1}{2x} \frac{\tanh (x/2)}{\cosh ^2(x/2)}, \end{aligned}$$

the coefficients $\Lambda _0$, $\Lambda _1$, $\Lambda _2$, and $\Lambda _3$ in ${\mathcal {E}}^{\text {GL}}_{D, h}$ are given by

3.20 $$\begin{aligned} \Lambda _0&{:}{=}\frac{\beta _c^2}{16} \int _{{\mathbb {R}}^3} \frac{\text {d}p}{(2\pi )^3} \; |(-2)\widehat{V\alpha _*}(p)|^2 \; \bigl ( g_1 (\beta _c(p^2-\mu )) + \frac{2}{3} \beta _c \, p^2\, g_2(\beta _c(p^2-\mu ))\bigr ), \end{aligned}$$

3.21 $$\begin{aligned} \Lambda _1&{:}{=}\frac{{\beta _{\text {c}}}^2}{4} \int _{{\mathbb {R}}^3} \frac{\text {d}p}{(2\pi )^3} \; |(-2)\widehat{V\alpha _*}(p)|^2 \; g_1({\beta _{\text {c}}}(p^2-\mu )), \end{aligned}$$

3.22 $$\begin{aligned} \Lambda _2&{:}{=}\frac{\beta _c}{8} \int _{{\mathbb {R}}^3} \frac{\text {d}p}{(2\pi )^3} \; \frac{|(-2)\widehat{V\alpha _*}(p)|^2}{\cosh ^2(\frac{\beta _c}{2}(p^2 -\mu ))}, \end{aligned}$$

3.23 $$\begin{aligned} \Lambda _3&{:}{=}\frac{\beta _c^2}{16} \int _{{\mathbb {R}}^3} \frac{\text {d}p}{(2\pi )^3} \; |(-2) \widehat{V\alpha _*}(p)|^4 \; \frac{g_1(\beta _c(p^2-\mu ))}{p^2-\mu }. \end{aligned}$$

It has been argued in [23, 24] that the coefficients $\Lambda _0$, $\Lambda _2$, and $\Lambda _3$ are positive. The coefficient $\Lambda _1$ can, in principle, have either sign. Its sign is related to the derivative of $T_{\text {c}}$ with respect to $\mu$, see the remark below Eq. (1.21) in [23].

We highlight the small factor $h^5$ in front of the $H_{\text {mag}}^1$-norm of $\Psi$ in the bound for |*R*(*h*)|. It is worse than the comparable estimate in [15, Theorem 3.5], which is a consequence of the presence of the periodic vector potential *A*. The error is, however, of the same size as the related error terms in [23, 24].

Theorem 3.6 provides us with a result for the BCS energy for temperatures of the form $T = {T_{\text {c}}}(1 - Dh^2)$ with $D \in {\mathbb {R}}$ fixed. In the proof of Theorem 2 (a) we also need the information that our system is superconducting for smaller temperatures. The precise statement is captured in the following proposition.

#### Proposition 3.7

(A priori bound on Theorem 2 (a)) Let Assumptions 1.1 and 1.3 (a) hold and let $T_0>0$. Then, there are constants $h_0>0$ and $D_0>0$ such that for all $0 < h \leqslant h_0$ and all temperatures *T* obeying

$$\begin{aligned} T_0 \leqslant T < {T_{\text {c}}}(1 - D_0 h^2), \end{aligned}$$

there is a BCS state $\Gamma$ with

3.24 $$\begin{aligned} {\mathcal {F}}^{\text {BCS}}_{h, T}(\Gamma ) - {\mathcal {F}}^{\text {BCS}}_{h, T}(\Gamma _0) < 0. \end{aligned}$$

### The upper bound on (1.22) and proof of Theorem 2 (a)

The results in the previous section can be used to prove the upper bound on (1.22) and Theorem 2 (a). These proof are almost literally the same as in the case of a constant magnetic field, and we therefore refer to [15, Section 3.3] for a detailed presentation.

## Proofs of the results in Sect. 3

### Schatten norm estimates for operators given by product kernels

During our trial state analysis, we frequently need Schatten norm estimates for operators defined by integral kernels of the form $\tau (x-y) \Psi ((x+y)/2)$. The relevant estimates are provided in the following lemma, whose proof can be found in [15, Lemma 4.1].

#### Lemma 4.1

Let $h>0$, let $\Psi$ be a gauge-periodic function on $Q_h$ and let $\tau$ be an even and real-valued function on ${\mathbb {R}}^3$. Moreover, let the operator $\alpha$ be defined via its integral kernel $\alpha (X,r) {:}{=}\tau (r)\Psi (X)$, i.e., $\alpha$ acts as

$$\begin{aligned} \alpha f(x)&= \int _{{\mathbb {R}}^3} \text {d}y \; \tau (x - y) \Psi \biggl (\frac{x+y}{2}\biggr ) f(y),&f&\in L^2({\mathbb {R}}^3). \end{aligned}$$

- Let $p \in \{2,4,6\}$. If $\Psi \in L_{\text {mag}}^p(Q_h)$ and $\tau \in L^{\frac{p}{p-1}}({\mathbb {R}}^3)$, then $\alpha \in {\mathcal {S}}^p$ and
  $$\begin{aligned} \Vert \alpha \Vert _p \leqslant C \; \Vert \tau \Vert _{\frac{p}{p-1}} \; \Vert \Psi \Vert _p. \end{aligned}$$
- For any $\nu > 3$, there is a $C_\nu >0$, independent of *h*, such that if  and $\Psi \in L_{\text {mag}}^6(Q_h)$, then $\alpha \in {\mathcal {S}}^\infty$ and

### Magnetic resolvent estimates

In this section we provide bounds for the resolvent kernel

4.1 $$\begin{aligned} G^z_h(x,y)&{:}{=}\frac{1}{z - (-\text {i}\nabla + {{\textbf {A}}}_h)^2 + \mu }(x,y),&x,y&\in {\mathbb {R}}^3 \end{aligned}$$

of the magnetic Laplacian that will be applied extensively in the proofs of Proposition 3.2 and Theorem 3.6. Our analysis is based on gauge-invariant perturbation theory in the spirit of Nenciu, see [42, Section V], and generalizes the analysis for the constant magnetic field in [26, Section 2] and [15, Section 4.4.1]. In case of a bounded magnetic vector potential, versions of some of our results appeared in [18].

We introduce the non-integrable phase factor, also called the Wilson line, by

4.2 $$\begin{aligned} \Phi _{{\textbf {A}}}(x,y) {:}{=}-\int _y^x {{\textbf {A}}}(u) \cdot \text {d}u {:}{=}-\int _0^1 \text {d}t\; {{\textbf {A}}}(y + t(x-y))\cdot (x-y). \end{aligned}$$

In case of the constant magnetic field the right side of (4.2) reduces to $\frac{{\textbf {B}}}{2}\cdot (x \wedge y)$. We also define the gauge-invariant kernel $g_h^z(x,y)$ via the equation

4.3 $$\begin{aligned} G_h^z(x,y) =&\, \text {e}^{\text {i}\Phi _{{{\textbf {A}}}_h}(x,y)} g_h^z(x,y),&x,y&\in {\mathbb {R}}^3. \end{aligned}$$

It should be compared to the translation-invariant (and gauge-invariant) kernel $g_B^z(x-y)$ introduced in [26, Eq. (4.27)] for the constant magnetic field. Its gauge-invariance makes it the natural starting point for a perturbative analysis.

The integral kernel of the operator $(z+\Delta + \mu )^{-1}$ will be denoted by $g_0^z(x-y)$. The main result of this subsection is the following proposition.

#### Proposition 4.2

Assume that ${{\textbf {A}}}= {{\textbf {A}}}_{e_3} + A$ with $A \in W^{3,\infty }({\mathbb {R}}^3,{\mathbb {R}}^3)$. For $t, \omega \in {\mathbb {R}}$ let

4.4 $$\begin{aligned} f(t, \omega ) {:}{=}\frac{|\omega | + |t + \mu |}{(|\omega | + (t + \mu )_-)^2}, \end{aligned}$$

where $x_- {:}{=}-\min \{x,0\}$. For any $a\geqslant 0$, there are constants $\delta _a, C_a > 0$ such that for all $t, \omega \in {\mathbb {R}}$ and for all $h \geqslant 0$ with $f(t, \omega ) \, h^2 \leqslant \delta _a$ there are (*h*-dependent) even $L^1({\mathbb {R}}^3)$-functions $\rho ^{\text {i}\omega + t}$, $\rho _\nabla ^{\text {i}\omega + t}$, $\tau ^{\text {i}\omega + t}$, and $\tau _\nabla ^{\text {i}\omega + t}$ such that

4.5 $$\begin{aligned} |g_h^{\text {i}\omega + t}(x,y)|&\leqslant \rho ^{\text {i}\omega + t} (x-y), \nonumber \\ |\nabla _x g_h^{\text {i}\omega + t}(x,y)|&\leqslant \rho _\nabla ^{\text {i}\omega + t} (x-y), \nonumber \\ |\nabla _y g_h^{\text {i}\omega + t}(x,y)|&\leqslant \rho _\nabla ^{-\text {i}\omega + t} (x-y), \end{aligned}$$

as well as

4.6 $$\begin{aligned} |g_h^{\text {i}\omega + t}(x,y) - g_0^{\text {i}\omega + t}(x - y)|&\leqslant \tau ^{\text {i}\omega + t} (x-y), \nonumber \\ | \nabla _x g_h^{\text {i}\omega + t}(x,y) - \nabla _x g_0^{\text {i}\omega + t}(x - y)|&\leqslant \tau _\nabla ^{\text {i}\omega + t} (x-y), \nonumber \\ |\nabla _y g_h^{\text {i}\omega + t}(x,y) - \nabla _y g_0^{\text {i}\omega + t}(x - y)|&\leqslant \tau _\nabla ^{-\text {i}\omega + t} (x-y). \end{aligned}$$

Furthermore, we have the estimates

4.7 $$\begin{aligned} \Vert \, |\cdot |^a \rho ^{\text {i}\omega + t} \Vert _1&\leqslant C_a \, f(t, \omega )^{1 + \frac{a}{2}}, \nonumber \\ \Vert \, |\cdot |^a \rho _\nabla ^{\text {i}\omega + t} \Vert _1&\leqslant C_a \, f(t,\omega )^{\frac{1}{2} + \frac{a}{2}} \, \Bigl [ 1 + \frac{|\omega | + |t - \mu |}{|\omega | + (t - \mu )_-} \Bigr ], \end{aligned}$$

and

4.8 $$\begin{aligned} \Vert \, |\cdot |^a \tau ^{\text {i}\omega + t} \Vert _1&\leqslant C_a \, h^3 \, f(t, \omega )^{\frac{5}{2} + \frac{a}{2}}, \nonumber \\ \Vert \, |\cdot |^a \tau _\nabla ^{\text {i}\omega + t} \Vert _1&\leqslant C_a \, h^3 \, f(t, \omega )^{2 + \frac{a}{2}} \Bigl [ 1 + \frac{|\omega | + |t - \mu |}{|\omega | + (t - \mu )_-} \Bigr ]. \end{aligned}$$

#### Remark 4.3

The bounds in the above proposition should be compared to those for $g_B^z(x)$ in [26, Lemma 10] (estimates without gradient) and [15, Lemma 4.5] (estimates with gradient). Although the kernel $g_h^z(x,y)$ defined in (4.3) is not translation-invariant, $|g_h^z(x,y)|$, $|g_h^z(x,y) - g_0^z(x-y)|$, and the same terms with a gradient can be bounded by translation-invariant kernels. Moreover, these translation-invariant kernels satisfy $L^1$-norm bounds that are mostly of the same quality as those obtained for the kernels $|g_B^z(x)|$, $|g_B^z(x) - g_0^z(x)|$, and the same terms with a gradient in [15, 26]. We highlight that, in comparison to [15, Eq. (4.34)], we lose a power of the small parameter *h* in the estimate in (4.8). This is due to the second term in the bracket in (4.20) below and it is in accordance with comparable bounds in [23] and [24]. The fact that the above kernels can be bounded from above by translation-invariant kernels is an important ingredient for the proofs of Proposition 3.2 and Theorem 3.6.

Before we give the proof of Proposition 4.2 we provide two lemmas. The first lemma concerns $L^1$-norm bounds for the kernel $g_0^z$ and its gradient. Its proof can be found in [15, Lemma 4.4]. The bound for $g_0^z$ (but not the one for $\nabla g_0^z$) appeared previously in [24, Lemma 9].

#### Lemma 4.4

Let $a > -2$. There is a constant $C_a >0$ such that for $t,\omega \in {\mathbb {R}}$, we have

4.9 $$\begin{aligned} \left\| \, |\cdot |^a g_0^{\text {i}\omega + t}\right\| _1&\leqslant C_a \; f(t, \omega )^{1+ \frac{a}{2}} \end{aligned}$$

with $f(t, \omega )$ in (4.4). Furthermore, for any $a > -1$, there is a constant $C_a >0$ with

4.10 $$\begin{aligned} \left\| \, |\cdot |^a \nabla g_0^{\text {i}\omega + t} \right\| _1 \leqslant C_a \; f(t, \omega )^{\frac{1}{2} + \frac{a}{2}} \; \Bigl [ 1 + \frac{|\omega | + |t+ \mu |}{|\omega | + (t + \mu )_-}\Bigr ]. \end{aligned}$$

The second lemma provides us with formulas for the gradient of the function $\Phi _{{\textbf {A}}}(x,y)$ defined in (4.2) with respect to *x* and *y*.

#### Lemma 4.5

Assume that ${{\textbf {A}}}= {{\textbf {A}}}_{e_3} + A$ with $A \in W^{2,\infty }({\mathbb {R}}^3,{\mathbb {R}}^3)$. Then we have

4.11 $$\begin{aligned} \nabla _x \Phi _{{\textbf {A}}}(x,y)&= -{{\textbf {A}}}(x) + {\widetilde{{{\textbf {A}}}}}(x,y),&\nabla _y \Phi _{{\textbf {A}}}(x,y)&= {{\textbf {A}}}(y) - {\widetilde{{{\textbf {A}}}}}(y,x), \end{aligned}$$

where

4.12 $$\begin{aligned} {\widetilde{{{\textbf {A}}}}} (x,y) {:}{=}\int _0^1 \text {d}t\; t {{\,\textrm{curl}\,}}{{\textbf {A}}}(y + t(x - y)) \wedge (x-y) \end{aligned}$$

is the transversal Poincaré gauge relative to *y*.

#### Remark 4.6

The function $\Phi _{{\textbf {A}}}(x,y)$ is a gauge transformation that relates ${{\textbf {A}}}(x)$ and ${\widetilde{{{\textbf {A}}}}}(x, y)$.

#### Proof of Lemma 4.5

From Morrey’s inequality we know that ${{\,\textrm{curl}\,}}{{\textbf {A}}}$ is Lipschitz continuous, and hence the line integral in (4.12) is well defined. For two vector fields *v* and *w* we have

4.13 $$\begin{aligned} \nabla (v \cdot w) = (v\cdot \nabla )w + (w\cdot \nabla ) v + v\wedge {{\,\textrm{curl}\,}}w + w\wedge {{\,\textrm{curl}\,}}v. \end{aligned}$$

We apply this equality for fixed $y\in {\mathbb {R}}^3$ to

$$\begin{aligned} v(x)&= \int _0^1\, \text {d}t\; {{\textbf {A}}}(y + t(x-y)),&w(x)&= x-y. \end{aligned}$$

Our definition implies ${{\,\textrm{curl}\,}}w = 0$ and we find that

4.14 $$\begin{aligned} -\nabla _x \Phi _{{\textbf {A}}}(x,y)&= \Bigl ( \int _0^1 \text {d}t\; {{\textbf {A}}}(y + t(x-y)) \cdot \nabla _x\Bigr ) (x-y) \nonumber \\&\quad + \left( (x-y) \cdot \nabla _x\right) \int _0^1 \text {d}t\; {{\textbf {A}}}(y + t(x-y)) + (x-y) \wedge \nonumber \\&\quad \int _0^1 \text {d}t\; t \, {{\,\textrm{curl}\,}}{{\textbf {A}}}(y + t(x-y)). \end{aligned}$$

The first term on the right side equals

4.15 $$\begin{aligned} \sum _{i=1}^3 \int _0^1\text {d}t\; {{\textbf {A}}}_i(y + t(x-y)) \, \partial _i (x-y)&= \int _0^1 \text {d}t\; {{\textbf {A}}}(y + t(x-y)). \end{aligned}$$

To rewrite the second term on the right side of (4.14), we use integration by parts and find

4.16 $$\begin{aligned} \left( (x-y)\cdot \nabla _x\right) \int _0^1 \text {d}t\; {{\textbf {A}}}(y + t(x-y))&= \int _0^1 \text {d}t\; t \, \frac{\text {d}}{\text {d}t} {{\textbf {A}}}(y + t(x-y)) \nonumber \\&= t \, {{\textbf {A}}}(y + t(x-y))\Big |_0^1 - \int _0^1 \text {d}t\; {{\textbf {A}}}(y + t(x-y)). \end{aligned}$$

Therefore, the sum of the terms in (4.15) and (4.16) equals ${{\textbf {A}}}(x)$. Since the last term on the right side of (4.14) equals $-\widetilde{{\textbf {A}}}(x,y)$, this proves the first equation in (4.11). The second equation follows from $\Phi _{{\textbf {A}}}(x,y) = -\Phi _{{\textbf {A}}}(y,x)$. $\square$

#### Proof of Proposition 4.2

We use the abbreviation $z = \text {i}\omega + t$ throughout the proof. In the first step we express $G_h^z(x,y)$ in (4.1) in terms of the kernel

4.17 $$\begin{aligned} {\widetilde{G}}_h^z(x,y) {:}{=}\text {e}^{\text {i}\Phi _{{{\textbf {A}}}_h}(x,y)} \, g_0^z(x-y). \end{aligned}$$

From (4.11) we know that

4.18 $$\begin{aligned} (-\text {i}\nabla _x + {{\textbf {A}}}_h(x)) \; \text {e}^{\text {i}\Phi _{{{\textbf {A}}}_h}(x,y)} = \text {e}^{\text {i}\Phi _{{{\textbf {A}}}_h}(x,y)}\; (-\text {i}\nabla _x + {\widetilde{{{\textbf {A}}}}}_h(x,y)), \end{aligned}$$

where ${\widetilde{{{\textbf {A}}}}}(x,y)$ in (4.12) is our vector potential in Poincaré gauge. Furthermore, a short computation shows that (4.18) implies the operator equation

4.19 $$\begin{aligned} (z - (-\text {i}\nabla + {{\textbf {A}}}_h)^2 + \mu ) {\widetilde{G}}_h^z = \mathbb {1}- T_h^z, \end{aligned}$$

where $T_h^z$ is the operator defined by the integral kernel

4.20 $$\begin{aligned} T_h^z (x,y) {:}{=}\text {e}^{\text {i}\Phi _{{{\textbf {A}}}_h}(x,y)} \bigl ( 2 \, {\widetilde{{{\textbf {A}}}}}_h(x,y) \cdot (-\text {i}\nabla _x) -\text {i}{{\,\textrm{div}\,}}_x \widetilde{{\textbf {A}}}_h(x,y) + |{\widetilde{{{\textbf {A}}}}}_h(x,y)|^2 \bigr ) g_0^z(x-y). \end{aligned}$$

Since $g_0^z$ is a radial function and the vector $\widetilde{{\textbf {A}}}(x,y)$ is perpendicular to $x-y$ the first term on the right side vanishes. The operator $T_h^z$ also appears in [42].

We claim that

4.21 $$\begin{aligned} |T_h^z(x,y) |&\leqslant M_{{\textbf {A}}}\, h^3 \; \eta ^z(x-y) \end{aligned}$$

holds, where $\eta ^z$ is the (*h*-dependent) function4.22and4.23We note that the bound in (4.21) holds as an equality with a similar function on the right side in the case of the constant magnetic field, see the proof of Lemma 10 in [26].

To prove (4.21), we first derive a bound for $|{{\,\textrm{div}\,}}_x {\widetilde{{{\textbf {A}}}}}(x,y)|$. For two vector fields *v* and *w* we have

$$\begin{aligned} {{\,\textrm{div}\,}}(v \wedge w) = {{\,\textrm{curl}\,}}(v) \cdot w - {{\,\textrm{curl}\,}}(w) \cdot v. \end{aligned}$$

We apply the above equality with the choice $v(x) = {{\,\textrm{curl}\,}}{{\textbf {A}}}(y + t(x-y))$, $w(x) = x-y$ and use ${{\,\textrm{curl}\,}}w =0$ to write

$$\begin{aligned} {{\,\textrm{div}\,}}_x \bigl ({{\,\textrm{curl}\,}}{{\textbf {A}}}(y+t(x-y)) \wedge (x-y)\bigr )&= t \; {{\,\textrm{curl}\,}}({{\,\textrm{curl}\,}}{{\textbf {A}}}) (y + t (x-y)) \cdot (x-y). \end{aligned}$$

Since ${{\,\textrm{curl}\,}}({{\,\textrm{curl}\,}}{{\textbf {A}}})$ is Lipschitz continuous, which follows from $A \in W^{3,\infty }({\mathbb {R}}^3,{\mathbb {R}}^3)$, we conclude that

4.24 $$\begin{aligned} |{{\,\textrm{div}\,}}_x {\widetilde{{{\textbf {A}}}}}(x,y)|&\leqslant \int _0^1\text {d}t\; t^2\, | {{\,\textrm{curl}\,}}({{\,\textrm{curl}\,}}{{\textbf {A}}})(y + t(x-y))\cdot (x-y)| \nonumber \\&\leqslant \Vert {{\,\textrm{curl}\,}}({{\,\textrm{curl}\,}}{{\textbf {A}}}) \Vert _\infty \; |x-y|. \end{aligned}$$

We also have

4.25 $$\begin{aligned} |{\widetilde{{{\textbf {A}}}}}(x,y)|&\leqslant \int _0^1 \text {d}t\; t \; |{{\,\textrm{curl}\,}}{{\textbf {A}}}(y + t(x-y)) \wedge (x-y)| \nonumber \\&\leqslant \Vert {{\,\textrm{curl}\,}}{{\textbf {A}}}\Vert _\infty \, |x-y|. \end{aligned}$$

In combination with $\Vert {{\,\textrm{curl}\,}}({{\,\textrm{curl}\,}}{{\textbf {A}}}_h)\Vert _\infty \leqslant M_{{\textbf {A}}}h^3$ and , this proves (4.21).

Next, we have a closer look at the function $\eta ^z$. Lemma 4.4 and the assumption $f(t, \omega ) \, h^2 \leqslant \delta _a$ imply the bound4.26In particular,

4.27 $$\begin{aligned} M_{{\textbf {A}}}\, h^3 \, \Vert \eta ^z\Vert _1 \leqslant C \, M_{{\textbf {A}}}\, h^3 \, f(t,\omega )^{\frac{3}{2}} \leqslant \frac{1}{2} \end{aligned}$$

for all allowed $t, \omega$ and *h* provided $\delta _a$ is chosen small enough. The bound in (4.21) and an application of Young’s inequality therefore show that the operator norm of $T_h^z$ satisfies

4.28 $$\begin{aligned} \Vert T_h^z\Vert _\infty \leqslant M_{{\textbf {A}}}\, h^3 \; \Vert \eta ^z \Vert _1 \leqslant \frac{1}{2}. \end{aligned}$$

We use (4.19) and this bound to write the resolvent of the magnetic Laplacian as

4.29 $$\begin{aligned} \frac{1}{z - (-\text {i}\nabla + {{\textbf {A}}}_h)^2 + \mu } = {\widetilde{G}}_h^z\; \frac{1}{1-T_h^z} = {\widetilde{G}}_h^z\; \sum _{j=0}^\infty \bigl (T_h^z\bigr )^j. \end{aligned}$$

This finishes the first step of our proof. In the second step we use (4.29) to prove the claimed bounds for the integral kernel $g_h^z(x,y)$. Our first goal is to prove the bounds for $g_h^z(x,y)$ without a gradient.

In the following we use the notation ${\mathcal {S}}_h^z = \sum _{j=1}^\infty ( T_h^z )^j$. Equation (4.29) allows us to write the kernel $g_h^z(x,y)$ as

4.30 $$\begin{aligned} g_h^z(x,y) = g_0^z(x-y) + \text {e}^{-\text {i}\Phi _{{{\textbf {A}}}_h}(x,y)} \int _{{\mathbb {R}}^3} \text {d}u \; \text {e}^{\text {i}\Phi _{{{\textbf {A}}}_h}(x, u)} g_0^z(x-u) \; {\mathcal {S}}_h^z(u,y). \end{aligned}$$

We use (4.21) to bound the integral kernel of the operator ${\mathcal {S}}_h^z$ by

4.31 $$\begin{aligned} | {\mathcal {S}}_h^z (x,y) | \leqslant \sum _{j=1}^{\infty } (M_{{{\textbf {A}}}} h^3)^{j} (\eta ^z)^{*j}(x-y) {=}{:}s^z(x-y), \end{aligned}$$

where $(\eta ^z)^{*j}$ denotes the *j*-fold convolution of $\eta ^z$ with itself. An application of the inequality

4.32 $$\begin{aligned} |x_1 + \cdots + x_j|^a \leqslant j^{(a-1)_+} \bigl (|x_1|^a + \cdots + |x_j|^a \bigr ) \end{aligned}$$

with $a \geqslant 0$ and $x_+ = \max \{ 0,x \}$ allows us to see that the function $s^z$ satisfies the pointwise bound

$$\begin{aligned} |x|^a \, s^z(x) \leqslant \sum _{j=1}^\infty (M_{{\textbf {A}}}h^3)^j \sum _{m=1}^j j^{(a-1)_+} \, \eta ^z * \cdots * \bigl ( |\cdot |^a \eta ^z\bigr ) * \cdots * \eta ^z(x). \end{aligned}$$

Here, $|\cdot |^a \eta ^z$ appears in the *m* $^{\text {th}}$ slot. An application of (4.26), (4.27), and Young’s inequality therefore implies

4.33 $$\begin{aligned} \int _{{\mathbb {R}}^3} \text {d}x \ |x|^a s^z(x)&\leqslant M_{{{\textbf {A}}}} h^3 \, \Vert \, |\cdot |^a \eta ^z\Vert _1 \sum _{j=1}^\infty \frac{j^{1 + (a-1)_+}}{2^{j-1}} \leqslant C_a \, h^3 \, f(t, \omega )^{\frac{3}{2} + \frac{a}{2}}. \end{aligned}$$

Let us also define the functions

$$\begin{aligned} \rho ^z(x)&{:}{=}|g_0^z(x)| + |g_0^z| *s^z(x),&\tau ^z(x)&{:}{=}|g_0^z| *s^z(x). \end{aligned}$$

From (4.30) we know that

$$\begin{aligned} |g_h^z(x,y)|&\leqslant \rho ^z(x-y),&|g_h^z(x,y) - g_0^z(x-y)|&\leqslant \tau ^z(x-y). \end{aligned}$$

The claimed bounds for the $L^1({\mathbb {R}}^3)$-norms of $\rho ^z$ and $\tau ^z$ in (4.7) and (4.8) follow from Lemma 4.4, (4.31), and (4.33). It remains to prove the bounds involving a gradient.

An application of Lemma 4.5 and (4.25) shows

$$\begin{aligned} |\nabla _x \text {e}^{-\text {i}\Phi _{{\textbf {A}}}(x,y)} \text {e}^{\text {i}\Phi _{{\textbf {A}}}(x,u)}| \leqslant |{\widetilde{{{\textbf {A}}}}}(x,y)| + |{\widetilde{{{\textbf {A}}}}}(x,u)| \leqslant C h^2 \left( |x-y| + |x -u| \right) . \end{aligned}$$

In combination with (4.30) and (4.31), this implies

$$\begin{aligned} |\nabla _x g_h^z(x,y)| \leqslant |\nabla g_0^z(x-y)| + C h^2 \int _{{\mathbb {R}}^3} \text {d}u \left( |x-u| + |u-y| \right) |g_0^z(x-u)| s^z(u-y). \end{aligned}$$

We denote the right side of the above equation by $\rho _{\nabla }^z(x-y)$. The claimed bound for $\rho _{\nabla }^z$ follows immediately from those for $g_0^z$ and $s^z$, see Lemma 4.4 and (4.33), and the assumption $f(t, \omega ) \, h^2 \leqslant \delta _a$. A bound for $|\nabla _x g_h^z(x,y) - \nabla g_0^z(x-y)|$ can be obtained similarly. The bounds for $|\nabla _y g_h^z(x,y)|$ and $|\nabla _y g_h^z(x,y) - \nabla g_0^z(x-y)|$ can be obtained when we use the identity $G_h^z(x,y) = \overline{G_h^{\overline{z}}(y,x)}$. This proves Proposition 4.2. $\square$

### Proof of Lemma 3.1

Let us recall the definition of $\Gamma _\Delta$ in (3.4). From this we infer that $\Gamma _\Delta$ is a gauge-periodic generalized fermionic one-particle density matrix. Consequently, we only need to verify the trace class condition in (1.8).

We use the identity $(\exp (x)+1)^{-1} = (1-\tanh (x/2))/2$ to write $\Gamma _\Delta$ as

4.34 $$\begin{aligned} \Gamma _\Delta&= \frac{1}{2} - \frac{1}{2} \tanh \biggl ( \frac{\beta }{2} H_\Delta \biggr ). \end{aligned}$$

Let us also recall the Mittag–Leffler series expansion

4.35 $$\begin{aligned} \tanh \biggl ( \frac{\beta }{2} z\biggr )&= -\frac{2}{\beta } \sum _{n\in {\mathbb {Z}}} \frac{1}{\text {i}\omega _n - z}, \end{aligned}$$

see e.g. [15, Eq. (3.12)]. Its convergence becomes manifest by combining the $+n$ and $-n$ terms. When we use (4.34) and the resolvent identity

4.36 $$\begin{aligned} (z- H_\Delta )^{-1} = (z-H_0)^{-1} + (z-H_0)^{-1} \; (H_\Delta - H_0)\; (z- H_\Delta )^{-1} \end{aligned}$$

we find

4.37 $$\begin{aligned} \Gamma _\Delta = \frac{1}{2} - \frac{1}{2} \tanh \biggl ( \frac{\beta }{2} H_\Delta \biggr ) = \frac{1}{2} + \frac{1}{\beta } \sum _{n\in {\mathbb {Z}}} \frac{1}{\text {i}\omega _n - H_\Delta } = \Gamma _0 + {\mathcal {O}}+ {\mathcal {Q}}_{T,{{\textbf {A}}}, W}(\Delta ). \end{aligned}$$

Here $\Gamma _0$ denotes the normal state in (1.12) and

4.38 $$\begin{aligned} {\mathcal {O}}&{:}{=}\frac{1}{\beta }\sum _{n\in {\mathbb {Z}}} \frac{1}{ \text {i}\omega _n - H_0} \delta \frac{1}{ \text {i}\omega _n - H_0}, \nonumber \\ {\mathcal {Q}}_{T,{{\textbf {A}}}, W}(\Delta )&{:}{=}\frac{1}{\beta }\sum _{n\in {\mathbb {Z}}} \frac{1}{ \text {i}\omega _n - H_0} \delta \frac{1}{ \text {i}\omega _n - H_0} \delta \frac{1}{ \text {i}\omega _n - H_\Delta } \end{aligned}$$

with $$\delta $$ in (3.3).

Since the diagonal components of the operator ${\mathcal {O}}$ equal zero, this term does not contribute to the 11-component $\gamma _{\Delta }$ of $\Gamma _{\Delta }$. In the following, we use the notation $\pi = - \text {i}\nabla + {{\textbf {A}}}_{{{\textbf {B}}}}$. To see that $(1 + \pi ^2) [{\mathcal {Q}}_{T, {{\textbf {A}}}, W}(\Delta )]_{11}$ is locally trace class, we use

$$\begin{aligned} \frac{1}{\text {i}\omega _n \pm H_0}\, \delta \, \frac{1}{\text {i}\omega _n \pm H_0} \, \delta&= \begin{pmatrix} \frac{1}{\text {i}\omega _n \pm {\mathfrak {h}}_{{{\textbf {A}}}, W}} \, \Delta \frac{1}{\text {i}\omega _n {\mp } \overline{{\mathfrak {h}}_{{{\textbf {A}}}, W}}} \, \overline{\Delta }&{} 0 \\ 0 &{} \frac{1}{\text {i}\omega _n {\mp } \overline{{\mathfrak {h}}_{{{\textbf {A}}}, W}}}\, \overline{\Delta }\frac{1}{\text {i}\omega _n \pm {\mathfrak {h}}_{{{\textbf {A}}}, W}} \, \Delta \end{pmatrix} \end{aligned}$$

to write it as

$$\begin{aligned} \bigl [ {\mathcal {Q}}_{T, {{\textbf {A}}}, W}(\Delta )\bigr ]_{11} = \frac{1}{\beta }\sum _{n\in {\mathbb {Z}}} \frac{1}{\text {i}\omega _n - {\mathfrak {h}}_{{{\textbf {A}}}, W}} \, \Delta \, \frac{1}{\text {i}\omega _n + \overline{{\mathfrak {h}}_{{{\textbf {A}}}, W}}} \, \overline{\Delta }\, \Bigl [ \frac{1}{\text {i}\omega _n - H_\Delta }\Bigr ]_{11}. \end{aligned}$$

The operator ${\mathfrak {h}}_{{{\textbf {A}}}, W}$ is defined in (3.2). An application of Hölder’s inequality in (2.1) shows that the local trace norm of the term inside the sum is bounded by

$$\begin{aligned} \Bigl \Vert (1 + \pi ^2) \frac{1}{\text {i}\omega _n - {\mathfrak {h}}_{{{\textbf {A}}}, W}}\Bigr \Vert _\infty \; \frac{1}{|\omega _n|^2} \; \Vert \Delta \Vert _2^2. \end{aligned}$$

Using Cauchy-Schwarz, we see that ${\mathfrak {h}}_{{{\textbf {A}}}, W} \leqslant C (1+\pi ^2)$, which implies that the operator norm in the above equation is bounded uniformly for $n \in {\mathbb {Z}}$. Since $|\omega _n|^{-2}$ is summable in *n* and $\Delta$ is locally Hilbert–Schmidt these considerations show that $(1+\pi ^2)[{\mathcal {Q}}_{T,{{\textbf {A}}}, W}(\Delta )]_{11}$ is locally trace class. It remains to show that $(1 + \pi ^2) \gamma _0$ with $\gamma _0$ in (1.12) is locally trace class.

To that end, we first note that

$$\begin{aligned} \left\| (1+\pi ^2) \gamma _0 \right\| _1 \leqslant \left\| (1+\pi ^2) \frac{1}{1 + {\mathfrak {h}}_{{{\textbf {A}}}, W} + \mu } \right\| _{\infty } \ \left\| (1+{\mathfrak {h}}_{{{\textbf {A}}}, W} + \mu ) \gamma _0 \right\| _{1}. \end{aligned}$$

We argue as above to see that the first norm on the right side is finite. To obtain a bound for the second norm, we first note that there is a constant $C>0$ such that $(1+x)(\exp (\beta (x-\mu ))+1)^{-1} \leqslant C \exp (-\beta x/2)$ holds for $x> a > -\infty$. The constant *C* depends on $\beta ,\mu$, and *a*. Accordingly,

$$\begin{aligned} \left\| (1+{\mathfrak {h}}_{{{\textbf {A}}}, W} + \mu ) \gamma _0 \right\| _{1} \leqslant C {{\,\textrm{Tr}\,}}\exp (-\beta {\mathfrak {h}}_{{{\textbf {A}}}, W}/2). \end{aligned}$$

From Corollary A.1.2 and Corollary B.13.3 in [48] we know that for any $t>0$ the operator $\exp (-t {\mathfrak {h}}_{{{\textbf {A}}}, W})$ has an integral kernel $k_{t}(x,y)$ that satisfies

$$\begin{aligned} \left\| k_t \right\| _{2,\infty }^2 = \mathop {\mathrm {ess \, sup}}\limits _{x \in {\mathbb {R}}^3} \int _{{\mathbb {R}}^3} \text {d}y \ | k_{t}(x,y)|^2 < \infty . \end{aligned}$$

Accordingly,We conclude that $(1+\pi ^2) \gamma _0$ is locally trace class. This ends the proof of Lemma 3.1.

### Proof of Lemma 3.4

We start by proving that $L_{T,{{\textbf {A}}},W}$ is a bounded linear operator on ${L^2(Q_h \times {\mathbb {R}}_{{\text {s}}}^3)}$. To that end, we first check that for $\Delta \in {L^2(Q_h \times {\mathbb {R}}_{{\text {s}}}^3)}$, we have $L_{T,{{\textbf {A}}},W} \Delta \in {\mathcal {S}}^2$. Using Hölder’s inequality for the trace per unit volume in (2.1) and $\omega _n = \pi (2n+1) T$, we see that

4.39 $$\begin{aligned} \sum _{n \in {\mathbb {Z}}} \left\| \frac{1}{\text {i}\omega _n - {\mathfrak {h}}_{{{\textbf {A}}}, W}} \, \Delta \, \frac{1}{\text {i}\omega _n + \overline{{\mathfrak {h}}_{{{\textbf {A}}}, W}}} \right\| _2 \leqslant \Vert \Delta \Vert _2 \sum _{n \in {\mathbb {Z}}} \frac{1}{\omega _n^2} \leqslant C \Vert \Delta \Vert _2 \sum _{n \in {\mathbb {Z}}} \frac{1}{(2n+1)^2}, \end{aligned}$$

which proves the claim.

Next, we show that $L_{T, {{\textbf {A}}}, W}\Delta$ satisfies (2.10). To that end, we need the identity

4.40 $$\begin{aligned} \frac{1}{\text {i}\omega _n + \overline{{\mathfrak {h}}_{{{\textbf {A}}}, W}} - \mu } (x,y) = -\frac{1}{-\text {i}\omega _n - {\mathfrak {h}}_{{{\textbf {A}}}, W} + \mu }(y,x). \end{aligned}$$

It follows from $\overline{ {\mathcal {R}}^* (x,y) } = {\mathcal {R}}(y,x)$ for a general operator ${\mathcal {R}}$ with kernel ${\mathcal {R}}(x,y)$ and

$$\begin{aligned} \frac{1}{z - \overline{{\mathfrak {h}}_{{{\textbf {A}}}, W}} + \mu } = \overline{\Bigl (\frac{1}{z - {\mathfrak {h}}_{{{\textbf {A}}}, W} + \mu } \Bigr )^*}. \end{aligned}$$

Using the coordinate transformation $(w_1,w_2) \mapsto (w_1+v,w_2+v)$ and the above identities for the resolvent kernel, we see that

4.41 $$\begin{aligned}&L_{T,{{\textbf {A}}},W} \Delta (X+v,r) = \frac{2}{\beta } \sum _{n \in {\mathbb {Z}}} \iint _{{\mathbb {R}}^3\times {\mathbb {R}}^3} \text {d}w_1 \text {d}w_2 \; \Delta \left( \frac{w_1+w_2}{2} + v, w_1 - w_2\right) \nonumber \\&\quad \times \frac{1}{\text {i}\omega _n - {\mathfrak {h}}_{{{\textbf {A}}}, W}+ \mu }\left( X + v + \frac{r}{2}, w_1\right) \frac{1}{-\text {i}\omega _n - {\mathfrak {h}}_{{{\textbf {A}}}, W}+ \mu } \left( X + v - \frac{r}{2}, w_2\right) \end{aligned}$$

holds. We highlight that we wrote $\Delta$ in terms of relative and center-of-mass coordinates in (4.41). We have $T(v) {\mathfrak {h}}_{{{\textbf {A}}}, W} T(v)^* = {\mathfrak {h}}_{{{\textbf {A}}}, W}$, $v \in \Lambda _h$, where *T*(*v*) is the magnetic translation in (1.3), and hence the same identity with ${\mathfrak {h}}_{{{\textbf {A}}}, W}$ replaced by its resolvent. In combination with

$$\begin{aligned}&\iint _{{\mathbb {R}}^3\times {\mathbb {R}}^3} \text {d}x \text {d}y \; \overline{\varphi (x)} \, [T(v)(z - {\mathfrak {h}}_{{{\textbf {A}}}, W})^{-1}T(v)^*](x,y) \, \varphi (y) \\&\qquad \qquad = \int _{{\mathbb {R}}^6} \text {d}x \text {d}y \; \overline{\varphi (x)} \, \text {e}^{\text {i}\frac{{{\textbf {B}}}}{2} \cdot (v \wedge (x-y))} \, (z - {\mathfrak {h}}_{{{\textbf {A}}}, W})^{-1}(x+v,y+v) \, \varphi (y), \end{aligned}$$

which holds for $z \in \rho ({\mathfrak {h}}_{{{\textbf {A}}}, W})$, this proves that the resolvent kernel of ${\mathfrak {h}}_{{{\textbf {A}}}, W}$ obeys the first relation in (1.7). When we combine this and the fact that $\Delta$ satisfies the second relation in (1.7), we see that we pick up the total phase

$$\begin{aligned} \text {e}^{\text {i}\frac{{{\textbf {B}}}}{2} \cdot (v\wedge (X + \frac{r}{2} - w_1))} \, \text {e}^{\text {i}\frac{{{\textbf {B}}}}{2} \cdot (v\wedge (X - \frac{r}{2} - w_2))} \, \text {e}^{\text {i}\frac{{{\textbf {B}}}}{2} \cdot (v\wedge (w_1 + w_2))} = \text {e}^{\text {i}{{\textbf {B}}}\cdot (v\wedge X)} \end{aligned}$$

in (4.41). This proves the first equation in (2.10) for $L_{T, {{\textbf {A}}}, W} \Delta (X,r)$.

To see that the second equation in (2.10) is true, we further observe that the Matsubara frequencies obey $-\omega _n = \omega _{-(n+1)}$. Hence, the index shift $n \mapsto -n-1$ and (4.40) imply the desired symmetry. This proves that $L_{T,{{\textbf {A}}},W}$ is a bounded linear operator on ${L^2(Q_h \times {\mathbb {R}}_{{\text {s}}}^3)}$.

To show that $N_{T,{{\textbf {A}}},W}(\Delta ) \in {\mathcal {S}}^2$, we use the bound

$$\begin{aligned} \sum _{n \in {\mathbb {Z}}}&\left\| \frac{1}{\text {i}\omega _n - {\mathfrak {h}}_{{{\textbf {A}}}, W}}\, \Delta \, \frac{1}{\text {i}\omega _n + \overline{{\mathfrak {h}}_{{{\textbf {A}}}, W}}} \, \overline{\Delta }\, \frac{1}{\text {i}\omega _n - {\mathfrak {h}}_{{{\textbf {A}}}, W}}\, \Delta \, \frac{1}{\text {i}\omega _n + \overline{{\mathfrak {h}}_{{{\textbf {A}}}, W}}} \right\| _2 \\&\qquad \qquad \qquad \qquad \qquad \qquad \qquad \quad \leqslant C \, \Vert \Delta \Vert _2 \ \Vert \Delta \Vert _{\infty }^2 \ \sum _{n \in {\mathbb {Z}}} \frac{1}{(2n+1)^4}. \end{aligned}$$

The proof of (2.10) for $N_{T,{{\textbf {A}}},W}(\Delta )$ goes along the same lines as that for $L_{T,{{\textbf {A}}},W} \Delta$. This proves Lemma 3.4.

### Proof of Theorem 3.6

Before we start with the proof of Theorem 3.6, we briefly mention the two main steps. In the first step we compute $\langle \Delta , L_{T, {{\textbf {A}}}, W} \Delta \rangle$. To that end, we decompose the operator $L_{T, {{\textbf {A}}}, W}$ into several increasingly simpler parts, which allows us to extract the quadratic terms in the GL functional. The related analysis can be found in Sects. 4.5.1–4.5.8. The quartic term in the GL functional emerges from $\langle \Delta , N_{T, {{\textbf {A}}}, W}(\Delta ) \rangle$. In the second step we study the nonlinear operator $N_{T, {{\textbf {A}}}, W}$ and introduce steps of simplification comparable to the ones for $L_{T, {{\textbf {A}}}, W}$. The related analysis starts in Sect. 4.5.9. The relation to the existing literature will be discussed mostly in Sects. 4.5.2, 4.5.6, and 4.5.9 after the relevant mathematical objects have been introduced.

#### Decomposition of $L_{T, {{\textbf {A}}}, W}$—separation of *W*

We use the resolvent equation in (4.36) to decompose the operator $L_{T, {{\textbf {A}}}, W}$ in (3.11) as

4.42 $$\begin{aligned} L_{T, {{\textbf {A}}}, W} = L_{T, {{\textbf {A}}}} + {\mathcal {W}}_{T, {{\textbf {A}}}} + {\mathcal {R}}_{T, {{\textbf {A}}},W}^{(2)}, \end{aligned}$$

where $$L_{T, {{\textbf {A}}}} = L_{T, {{\textbf {A}}}, 0}$$,

4.43 $$\begin{aligned} {\mathcal {W}}_{T, {{\textbf {A}}}} \Delta&{:}{=}-\frac{2}{\beta }\sum _{n\in {\mathbb {Z}}} \Bigl [ \frac{1}{\text {i}\omega _n - {\mathfrak {h}}_{{\textbf {A}}}} \, W_h \, \frac{1}{\text {i}\omega _n - {\mathfrak {h}}_{{\textbf {A}}}} \, \Delta \, \frac{1}{\text {i}\omega _n + \overline{{\mathfrak {h}}_{{\textbf {A}}}}} \nonumber \\&\quad - \frac{1}{\text {i}\omega _n - {\mathfrak {h}}_{{\textbf {A}}}} \, \Delta \, \frac{1}{\text {i}\omega _n + \overline{{\mathfrak {h}}_{{\textbf {A}}}}} \, W_h \, \frac{1}{\text {i}\omega _n + \overline{{\mathfrak {h}}_{{\textbf {A}}}}}\Bigr ], \end{aligned}$$

and

4.44 $$\begin{aligned} {\mathcal {R}}_{T, {{\textbf {A}}}, W}^{(2)} \Delta&{:}{=}-\frac{2}{\beta }\sum _{n\in {\mathbb {Z}}} \Bigl [ \frac{1}{\text {i}\omega _n - {\mathfrak {h}}_{{\textbf {A}}}} \, W_h \, \frac{1}{\text {i}\omega _n - {\mathfrak {h}}_{{\textbf {A}}}} \, W_h \, \frac{1}{\text {i}\omega _n - {\mathfrak {h}}_{{{\textbf {A}}}, W}} \, \Delta \, \frac{1}{\text {i}\omega _n + \overline{{\mathfrak {h}}_{{\textbf {A}}}}} \nonumber \\&\qquad + \frac{1}{\text {i}\omega _n - {\mathfrak {h}}_{{\textbf {A}}}} \, \Delta \, \frac{1}{\text {i}\omega _n + \overline{{\mathfrak {h}}_{{\textbf {A}}}}} \, W_h \, \frac{1}{\text {i}\omega _n + \overline{{\mathfrak {h}}_{{{\textbf {A}}}, W}}} \, W_h \, \frac{1}{\text {i}\omega _n + \overline{{\mathfrak {h}}_{{\textbf {A}}}}} \nonumber \\&\qquad -\frac{1}{\text {i}\omega _n - {\mathfrak {h}}_{{\textbf {A}}}} \, W_h \, \frac{1}{\text {i}\omega _n - {\mathfrak {h}}_{{{\textbf {A}}}, W}} \, \Delta \, \frac{1}{\text {i}\omega _n + \overline{{\mathfrak {h}}_{{{\textbf {A}}}, W}}} \, W_h \, \frac{1}{\text {i}\omega _n + \overline{{\mathfrak {h}}_{{\textbf {A}}}}} \Bigr ]. \end{aligned}$$

In the special case of a constant magnetic field, the operator $L_{T, {{\textbf {A}}}}$ appeared for the first time in [26]. The operator ${\mathcal {W}}_{T, 0}$ (case of no external magnetic fields) was studied in [19]. In the sections 4.5.2-4.5.4 we analyze $\langle \Delta , L_{T, {{\textbf {A}}}} \Delta \rangle$ and extract the first and the third term in the GL functional from it. Afterwards, we study in Sects. 4.5.5 and 4.5.6 the quadratic form $\langle \Delta , {\mathcal {W}}_{T, {{\textbf {A}}}} \Delta \rangle$, which contributes the second term of the GL functional. Finally, in Sect. 4.5.8, we collect the results of the previous sections and provide a bound for $\langle \Delta , {\mathcal {R}}_{T, {{\textbf {A}}}, W}^{(2)}\Delta \rangle$ showing that this term does not contribute to the GL functional.

#### A representation formula for $L_{T,{{\textbf {A}}}}$ and an outlook on the quadratic terms

In the following subsections we compute the contribution from $\langle \Delta , L_{T, {{\textbf {A}}}} \Delta \rangle$ to the Ginzburg–Landau energy. Our analysis is based on the following representation formula for the operator $L_{T, {{\textbf {A}}}}$, which characterizes its action solely in terms of relative and center-of-mass coordinates.

##### Lemma 4.7

The operator $L_{T,{{\textbf {A}}}} :{L^2(Q_h \times {\mathbb {R}}_{{\text {s}}}^3)}\rightarrow {L^2(Q_h \times {\mathbb {R}}_{{\text {s}}}^3)}$ acts as

$$\begin{aligned} (L_{T,{{\textbf {A}}}}\alpha ) (X,r)&= \iint _{{\mathbb {R}}^3\times {\mathbb {R}}^3} \text {d}Z \text {d}s \; k_{T, {{\textbf {A}}}}(X, Z, r, s) \; (\text {e}^{\text {i}Z \cdot (-\text {i}\nabla _X)}\alpha ) (X,s) \end{aligned}$$

with

4.45 $$\begin{aligned} k_{T,{{\textbf {A}}}}(X, Z, r, s) {:}{=}\frac{2}{\beta }\sum _{n\in {\mathbb {Z}}} k_{T, {{\textbf {A}}}}^n(X, Z, r, s) \; \text {e}^{\text {i}{\widetilde{\Phi }}_{{{\textbf {A}}}_h}(X, Z, r, s)}, \end{aligned}$$

where

4.46 $$\begin{aligned} k_{T, {{\textbf {A}}}}^n(X, Z, r, s)&{:}{=}\, g_h^{\text {i}\omega _n} \bigl (X + \frac{r}{2}, X + Z + \frac{s}{2}\bigr ) \; g_h^{-\text {i}\omega _n}\bigl (X - \frac{r}{2}, X + Z - \frac{s}{2}\bigr ) \end{aligned}$$

with $g_h^z$ in (4.3) and

4.47 $$\begin{aligned} {\widetilde{\Phi }}_{{\textbf {A}}}(X, Z, r, s)&{:}{=}\, \Phi _{{\textbf {A}}}\bigl (X + \frac{r}{2}, X + Z + \frac{s}{2}\bigr ) + \Phi _{{\textbf {A}}}\bigl (X - \frac{r}{2}, X + Z - \frac{s}{2}\bigr ) \end{aligned}$$

with $$\Phi _{{\textbf {A}}}$$ in (4.2).

##### Remark 4.8

Lemma 4.7 should be compared to the representation formula in [26, Lemma 11] for the operator $L_{T, B}$ in [26, Eq. (8)]. The differences between the two representation formulas are related to the fact that our magnetic field is non-constant. Accordingly, the kernel $g_h^z(x,y)$ is not translation-invariant and $\Phi _{{{\textbf {A}}}_h}(x,y)$ does not simply equal $\frac{{\textbf {B}}}{2} \cdot ( x \wedge y)$. This results in the dependence of the function $k_{T, {{\textbf {A}}}}^n$ on the coordinate *X* and in the fact that the representation formula in Lemma 4.7 is not symmetric under the transformation $Z \mapsto -Z$. As a consequence, the operator $\cos (Z\cdot \Pi )$ in [26, Lemma 11] is replaced by $\exp (\text {i}Z\cdot (-\text {i}\nabla _X))$. Both, the cosine function and the full magnetic momentum operator in its argument, will be recovered at a later stage, see (4.76) below. The main guiding principle behind the definition of the above representation formula and its subsequent analysis is that we should think of $\Phi _{{{\textbf {A}}}_h}(x,y)$ as a generalization of $\frac{{\textbf {B}}}{2} \cdot ( x \wedge y)$. The latter has more convenient algebraic properties, which is responsible for much of the simplicity of the analysis in [15, 26] in comparison to the present work. In case of $\Phi _{{{\textbf {A}}}_h}(x,y)$ we do computations as if similar relations were satisfied and afterwards carefully bound the emergent remainder terms.

##### Proof of Lemma 4.7

With (4.40) applied to $W =0$ the integral kernel of $L_{T, {{\textbf {A}}}}$ can be written as

$$\begin{aligned} L_{T,{{\textbf {A}}}} \alpha (x, y)&= \frac{2}{\beta }\sum _{n\in {\mathbb {Z}}} \iint _{{\mathbb {R}}^3\times {\mathbb {R}}^3} \text {d}u\text {d}v\; G_h^{\text {i}\omega _n} (x, u) \, G_h^{-\text {i}\omega _n}(y, v) \, \alpha (u,v). \end{aligned}$$

We note that $\alpha$ and $L_{T,{{\textbf {A}}}} \alpha$ are not yet written in terms of relative and center-of-mass coordinates, which will be done in the next step. To that end, we define the coordinates $X =\frac{x+y}{2}$, $r=x-y$,

4.48 $$\begin{aligned} u&= X + Z + \frac{s}{2},&v = X + Z - \frac{s}{2}, \end{aligned}$$

and introduce the notation $\zeta _X^r {:}{=}X + \frac{r}{2}$. This allows us to write the above equation as

$$\begin{aligned} L_{T,{{\textbf {A}}}} \alpha (X, r)&= \frac{2}{\beta }\sum _{n\in {\mathbb {Z}}} \iint _{{\mathbb {R}}^3\times {\mathbb {R}}^3} \text {d}Z\text {d}s\; G_h^{\text {i}\omega _n} (\zeta _X^r, \zeta _{X+Z}^s) \, G_h^{-\text {i}\omega _n}(\zeta _X^{-r}, \zeta _{X+Z}^{-s}) \, \alpha (X + Z, s). \end{aligned}$$

We highlight that, by a slight abuse of notation, we denoted the kernels of $\alpha$ and $L_{T,{{\textbf {A}}}} \alpha$ when expressed in terms of relative and center-of-mass coordinates still by the same symbols. When we use the relation $G^z_h(x,y) = \exp ( \text {i}\Phi _{{{\textbf {A}}}}(x,y) ) g_h^z(x,y)$ and

$$\begin{aligned} \alpha (X+Z,s) = \text {e}^{\text {i}Z\cdot (-\text {i}\nabla _X)} \alpha (X, s), \end{aligned}$$

we see that the above identity implies the claimed formula. $\square$

We analyze the operator $L_{T,{{\textbf {A}}}}$ in four steps. In the first three steps, we introduce three operators of increasing simplicity in their dependence on ${{\textbf {A}}}_h$. More precisely, we write it as

4.49 $$\begin{aligned} L_{T,{{\textbf {A}}}} = (L_{T,{{\textbf {A}}}} - {\widetilde{L}}_{T,{{\textbf {A}}}}) + (\widetilde{L}_{T,{{\textbf {A}}}} - {\widetilde{M}}_{T,{{\textbf {A}}}}) + ({\widetilde{M}}_{T,{{\textbf {A}}}} - M_{T,{{\textbf {A}}}}) + M_{T, {{\textbf {A}}}} \end{aligned}$$

with the operators ${\widetilde{L}}_{T,{{\textbf {A}}}}$, ${\widetilde{M}}_{T, {{\textbf {A}}}}$, and $M_{T,{{\textbf {A}}}}$ defined below in (4.50), (4.76), and (4.108), respectively. As we will show, the operators in brackets in (4.49) do not contribute to the GL functional. The operator ${\widetilde{L}}_{T,{{\textbf {A}}}}$ is obtained from $L_{T,{{\textbf {A}}}}$ when we replace the kernels $g_h^{z}(x,y)$ in (4.46) by $g_0^z(x-y)$. To obtain the operator ${\widetilde{M}}_{T,{{\textbf {A}}}}$ from ${\widetilde{L}}_{T,{{\textbf {A}}}}$, we need to replace the phase factor in the definition of $k_{T,{{\textbf {A}}}}^n(X,Z,r,s)$ by $\exp ( -\text {i}(r-s) \cdot D {{\textbf {A}}}_h(X) (r+s)/4 )$, where $D {{\textbf {A}}}_h$ denotes the Jacobi matrix of ${{\textbf {A}}}_h$, and $\exp (\text {i}Z \cdot (-\text {i}\nabla _X))$ by $\cos ( Z \cdot \Pi _{{{\textbf {A}}}_h})$. Finally, $M_{T,{{\textbf {A}}}}$ emerges when we replace $\exp ( -\text {i}(r-s) \cdot D {{\textbf {A}}}_h(X) (r+s)/4 )$ in the definition of ${\widetilde{M}}_{T, {{\textbf {A}}}}$ by 1. In the fourth and final step we extract the quadratic terms in the GL functional (except the one proportional to *W*) as well as a term that cancels the last term on the left side of (3.18) from $\langle \Delta , M_{T,{{\textbf {A}}}} \Delta \rangle$. To that end, we expand the operator $\cos ( Z \cdot \Pi _{{{\textbf {A}}}_h})$ up to second order in powers $Z \cdot \Pi _{{{\textbf {A}}}_h}$ and use $T = T_{\text {c}}(1 - D h^2)$.

In the case of a constant magnetic field a similar decomposition of $L_{T,{{\textbf {A}}}}$ has been introduced for the first time in [26]. In this reference the operator $\widetilde{L}_{T,{{\textbf {A}}}}$ is called $M_{T,{{\textbf {A}}}}$ and our $M_{T,{{\textbf {A}}}}$ is called $N_{T,{{\textbf {B}}}}$. We did not follow the notation in [26] because the symbol $N_{T,{{\textbf {A}}},W}$ is reserved for the nonlinear term in our paper. The decomposition of $L_{T,{{\textbf {A}}}}$ in [26] has also been used in [15]. In comparison to these two references we have an additional term (the operator ${\widetilde{M}}_{T,{{\textbf {A}}}}$) in our decomposition of $L_{T,{{\textbf {A}}}}$, which is a consequence of the fact that we are dealing with a general magnetic field. Generally speaking, the magnetic vector potential ${{\textbf {A}}}_h$ is more difficult to treat than ${{\textbf {A}}}_{{{\textbf {B}}}}$ because several algebraic relations that hold for the latter do not hold for the former. Our main contribution in this section is that we overcome the related mathematical difficulties in the computation of the above terms. Another main difference between our work and [26] is that we additionally need $H_{\text {mag}}^1(Q_h)$-norm estimates. In the special case of a constant magnetic field such bounds have been proved in [15]. It should also be noted that $L_{T,{{\textbf {A}}}}$ acts on $L^2({\mathbb {R}}^6)$ in [26], while it acts on ${L^2(Q_h \times {\mathbb {R}}_{{\text {s}}}^3)}$ in our case and in [15].

#### Approximation of $L_{T, {{\textbf {A}}}}$

**4.5.3.1 The operator **$${\widetilde{L}}_{T, {{\textbf {A}}}}$$.

We define the operator ${\widetilde{L}}_{T, {{\textbf {A}}}}$ by

4.50 $$\begin{aligned} {\widetilde{L}}_{T, {{\textbf {A}}}}\alpha (X,r)&{:}{=}\iint _{{\mathbb {R}}^3 \times {\mathbb {R}}^3} \text {d}Z \text {d}s \; {\widetilde{k}}_{T, {{\textbf {A}}}}(X, Z, r,s) \; ( \text {e}^{\text {i}Z \cdot (-\text {i}\nabla _X)} \alpha )(X,s) \end{aligned}$$

with

4.51 $$\begin{aligned} {\widetilde{k}}_{T, {{\textbf {A}}}} (X, Z, r,s) {:}{=}\frac{2}{\beta }\sum _{n\in {\mathbb {Z}}} k_T^n(Z, r-s) \; \text {e}^{\text {i}{\widetilde{\Phi }}_{{{\textbf {A}}}_h}(X, Z, r, s)}, \end{aligned}$$

where ${\widetilde{\Phi }}_{{\textbf {A}}}$ is defined in (4.47) and

4.52 $$\begin{aligned} k_T^n(Z, r) {:}{=}\, k_{T, 0}^n(0, Z, r, 0) = g_0^{\text {i}\omega _n}\biggl (Z - \frac{r}{2} \biggr ) \, g_0^{-\text {i}\omega _n}\biggl ( Z + \frac{r}{2} \biggr ). \end{aligned}$$

The following proposition allows us to replace the operator $L_{T, {{\textbf {A}}}}$ by ${\widetilde{L}}_{T, {{\textbf {A}}}}$ in the computation of the BCS energy of our trial state.

##### Proposition 4.9

Let ${{\textbf {A}}}= {{\textbf {A}}}_{e_3} + A$ with $A \in W^{2,\infty }({\mathbb {R}}^3,{\mathbb {R}}^3)$ periodic, let $|\cdot |^k V\alpha _*\in L^2({\mathbb {R}}^3)$ for $k \in \{ 0,1 \}$, $\Psi \in H_{\text {mag}}^1(Q_h)$, and denote $\Delta \equiv \Delta _\Psi$ as in (3.1). For any $T_0 > 0$ there is $h_0>0$ such that for any $0 < h \leqslant h_0$ and any $T\geqslant T_0$ we have

$$\begin{aligned} \Vert L_{T, {{\textbf {A}}}} \Delta - {\widetilde{L}}_{T, {{\textbf {A}}}} \Delta \Vert _{H^1(Q_h \times {\mathbb {R}}_{{\text {s}}}^3)}^2 \leqslant C \; h^8 \; \left( \Vert V\alpha _*\Vert _2^2 + \Vert \ |\cdot | V\alpha _*\Vert _2^2 \right) \; \Vert \Psi \Vert _{H_{\text {mag}}^1(Q_h)}^2. \end{aligned}$$

##### Remark 4.10

In order to prove Theorem 3.6, we only need the bound

$$\begin{aligned} | \langle \Delta , (L_{T, {{\textbf {A}}}} - {\widetilde{L}}_{T, {{\textbf {A}}}})\Delta \rangle | \leqslant C \; h^5 \; \left( \Vert V\alpha _*\Vert _2^2 + \Vert \ |\cdot | V\alpha _*\Vert _2^2 \right) \; \Vert \Psi \Vert _{H_{\text {mag}}^1(Q_h)}^2, \end{aligned}$$

which is a direct consequence of Cauchy–Schwarz, Proposition 4.9, Lemma 4.1, and (2.7). We prove the more general statement in Proposition 4.9 here because we need it in the proof of Proposition 3.2 in Sect. 4.6 below.

We recall the definition of the Matsubara frequencies $\omega _n$ in (3.10), of $g_0^z$ in (4.3), and of the functions $\rho ^z$, $\tau ^z$, $\rho _{\nabla }^z$, and $\tau _{\nabla }^z$ in Proposition 4.2. With this, let us define the functions

4.53 $$\begin{aligned} F_{T,h}^{a}&{:}{=}\frac{2}{\beta }\sum _{m=0}^a \sum _{n\in {\mathbb {Z}}} \bigl (|\cdot |^m \, \tau ^{\text {i}\omega _n} \bigr ) * \rho ^{-\text {i}\omega _n} + \tau ^{\text {i}\omega _n} * \bigl (|\cdot |^m \, \rho ^{-\text {i}\omega _n} \bigr ) \nonumber \\&\quad + \bigl (|\cdot |^m \, |g_0^{\text {i}\omega _n}|\bigr ) * \tau ^{-\text {i}\omega _n} + |g_0^{\text {i}\omega _n}| * \bigl (|\cdot |^m \, \tau ^{-\text {i}\omega _n} \bigr ) \end{aligned}$$

with $$a \in {\mathbb {N}}_0$$ and

4.54 $$\begin{aligned} G_{T, h}&{:}{=}\frac{2}{\beta }\sum _{\# \in \{ \pm \}} \sum _{n\in {\mathbb {Z}}} \tau _\nabla ^{\# \text {i}\omega _n} * \rho ^{-\text {i}\omega _n} + \tau ^{\text {i}\omega _n} * \rho _\nabla ^{-\# \text {i}\omega _n} + |\nabla g_0^{\# \text {i}\omega _n}| * \tau ^{-\text {i}\omega _n} + |g_0^{\text {i}\omega _n}| * \tau _\nabla ^{-\# \text {i}\omega _n}, \end{aligned}$$

which play a prominent role in the proof of Proposition 4.9.

We claim that for any $T_0 > 0$ there is $h_0>0$ such that for any $0 < h \leqslant h_0$ and any $T\geqslant T_0$ we have

4.55 $$\begin{aligned} \Vert F_{T, h}^a \Vert _1 + \Vert G_{T, h} \Vert _1 \leqslant C_a \, h^3. \end{aligned}$$

To prove this claim, we apply Young’s inequality, Proposition 4.2, and Lemma 4.4, and note that the function $f(t, \omega )$ in (4.4) obeys the estimate

4.56 $$\begin{aligned} f(0, \omega _n)&\leqslant C \; |2n+1|^{-1}. \end{aligned}$$

Moreover,

4.57 $$\begin{aligned} 1+\frac{|\omega _n| + |\mu |}{|\omega _n| + \mu _-} \leqslant C. \end{aligned}$$

In combination, these considerations prove our claim. We are now prepared to give the proof of the above proposition.

##### Proof of Proposition 4.9

We have

4.58 $$\begin{aligned} \Vert L_{T, {{\textbf {A}}}}\Delta - {\widetilde{L}}_{T, {{\textbf {A}}}} \Delta \Vert _{H^1(Q_h \times {\mathbb {R}}_{{\text {s}}}^3)}^2&= \Vert L_{T, {{\textbf {A}}}}\Delta - {\widetilde{L}}_{T, {{\textbf {A}}}} \Delta \Vert _2^2 \nonumber \\&\quad + \Vert \Pi (L_{T, {{\textbf {A}}}}\Delta - {\widetilde{L}}_{T, {{\textbf {A}}}} \Delta )\Vert _2^2 + \Vert {\widetilde{\pi }}(L_{T, {{\textbf {A}}}}\Delta - {\widetilde{L}}_{T, {{\textbf {A}}}} \Delta )\Vert _2^2 \end{aligned}$$

and we claim that the first term on the right side satisfies

4.59 $$\begin{aligned} \Vert L_{T, {{\textbf {A}}}}\Delta - {\widetilde{L}}_{T, {{\textbf {A}}}} \Delta \Vert _2^2&\leqslant 4 \; \Vert \Psi \Vert _2^2 \; \Vert F_{T, h}^0 * |V\alpha _*| \, \Vert _2^2. \end{aligned}$$

Using Young’s inequality, (2.7), and (4.55), we see that the right side of (4.59) is bounded by a constant times $h^8 \Vert V\alpha _*\Vert _2^2 \Vert \Psi \Vert _{H_{\text {mag}}^1(Q_h)}^2$. If (4.59) holds, this therefore proves the claimed bound for this term.

To prove (4.59), we start by noting that4.60Since the norm of the operator $\text {e}^{\text {i}Z\cdot (-\text {i}\nabla _X)}$ equals 1, we have4.61Consequently, (4.60) yields

4.62 $$\begin{aligned}&\Vert L_{T, {{\textbf {A}}}}\Delta - {\widetilde{L}}_{T, {{\textbf {A}}}}\Delta \Vert _2^2 \leqslant 4\; \Vert \Psi \Vert _2^2 \nonumber \\&\quad \times \int _{{\mathbb {R}}^3 }\text {d}r\, \Bigl | \iint _{{\mathbb {R}}^3 \times {\mathbb {R}}^3} \text {d}Z\text {d}s \; \mathop {\mathrm {ess \, sup}}\limits _{X\in {\mathbb {R}}^3} |(k_{T, {{\textbf {A}}}} - {\widetilde{k}}_{T, {{\textbf {A}}}}) (X, Z, r, s)|\; |V\alpha _*(s)|\Bigr |^2. \end{aligned}$$

We note that

4.63 $$\begin{aligned}&k_{T, {{\textbf {A}}}}^n(X, Z, r, s) - k_T^n(Z, r - s) \nonumber \\&\quad = \bigl ( g_h^{\text {i}\omega _n} - g_0^{\text {i}\omega _n}\bigr ) \bigl ( X+ \frac{r}{2}, X + Z+ \frac{s}{2}\bigr ) \, g_h^{-\text {i}\omega _n} \bigl ( X - \frac{r}{2}, X + Z - \frac{s}{2}\bigr ) \nonumber \\&\qquad + g_0^{\text {i}\omega _n} \bigl (Z - \frac{r-s}{2}\bigr ) \, \bigl ( g_h^{-\text {i}\omega _n} - g_0^{-\text {i}\omega _n} \bigr ) \bigl ( X - \frac{r}{2} , X + Z - \frac{s}{2}\bigr ) \end{aligned}$$

and hence, by Proposition 4.2, the integrand in (4.62) is bounded by

4.64 $$\begin{aligned} \bigl |(k_{T, {{\textbf {A}}}} - {\widetilde{k}}_{T, {{\textbf {A}}}}) (X, Z, r, s)\bigr |&\leqslant \frac{2}{\beta }\, \smash {\sum _{n\in {\mathbb {Z}}}} \, \bigl [ \tau ^{\text {i}\omega _n} \bigl ( Z - \frac{r-s}{2}\bigr ) \; \rho ^{-\text {i}\omega _n} \bigl ( Z + \frac{r-s}{2}\bigr ) \nonumber \\&\quad + |g_0^{\text {i}\omega _n}|\bigl ( Z -\frac{r-s}{2}\bigr )\; \tau ^{-\text {i}\omega _n} \bigl ( Z +\frac{r-s}{2}\bigr )\bigr ]. \end{aligned}$$

We combine (4.64) and the fact that the functions in (4.64) are even (see Proposition 4.2), to see that

4.65 $$\begin{aligned} \int _{{\mathbb {R}}^3} \text {d}Z \; \mathop {\mathrm {ess \, sup}}\limits _{X\in {\mathbb {R}}^3} |(k_{T, {{\textbf {A}}}} - {\widetilde{k}}_{T, {{\textbf {A}}}})(X, Z, r, s)| \leqslant F_{T, h}^0(r-s), \end{aligned}$$

where $F_{T, {{\textbf {A}}}}^0$ is the function in (4.53). When we apply (4.65) to (4.62), we obtain (4.59).

Let us pause for a moment and highlight the main idea behind the above bound because it will reappear frequently in the subsequent analysis. The kernels in (4.63) are not translation-invariant. From Proposition 4.2 we know, however, that they can by bounded by translation-invariant kernels. When we do this, we see that we obtain convolutions of translation-invariant kernels after the integration over *Z* has been carried out. These convolutions can now be estimated with the $L^1$-norm bounds in Proposition 4.2. We note that this emergent simplicity is difficult to find when working in the operator picture. Our analysis is inspired by the analysis for the constant magnetic field in [26], where much of the above structure is more apparent.

For the second term on the right side of (4.58), we claim the bound

4.66 $$\begin{aligned} \Vert \Pi (L_{T, {{\textbf {A}}}}\Delta - {\widetilde{L}}_{T, {{\textbf {A}}}}\Delta )\Vert _2^2&\leqslant C \, h^2 \; \Vert (F_{T, h}^1 + G_{T, h} ) * |V\alpha _*| \,\Vert _2^2 \; \Vert \Psi \Vert _{H_{\text {mag}}^1(Q_h)}^2 \nonumber \\&\leqslant C \, h^8 \; \Vert V\alpha _*\Vert _2^2 \; \Vert \Psi \Vert _{H_{\text {mag}}^1(Q_h)}^2. \end{aligned}$$

The second inequality follows from Young’s inequality and (4.55). To prove the first inequality in (4.66), we note that the gradient can act either on $\Psi$ or on $k_{T,{{\textbf {A}}}}-{\widetilde{k}}_{T,{{\textbf {A}}}}$ and we start by considering the term, where it acts on $\Psi$. We therefore apply (4.60) with $\exp (\text {i}Z \cdot (-\text {i}\nabla _X))$ replaced by $\Pi _X \, \exp (\text {i}Z \cdot (-\text {i}\nabla _X))$ (we recall that $\Pi _X = -\text {i}\nabla _X + 2 {{\textbf {A}}}_{{{\textbf {B}}}}(X)$) and replace (4.61) byFrom a direct computation, we know that

4.67 $$\begin{aligned} \Pi _X\, \text {e}^{\text {i}Z\cdot (-\text {i}\nabla _X)} = \text {e}^{\text {i}Z \cdot (-\text {i}\nabla _X)} \bigl [\Pi _X - {{\textbf {B}}}\wedge Z\bigr ]. \end{aligned}$$

Using this and (2.7), we find

4.68 $$\begin{aligned} \Vert \Pi _X \, \text {e}^{\text {i}Z\cdot (-\text {i}\nabla _X)} \Psi \Vert _2&\leqslant \Vert \Pi \Psi \Vert _2 + |{{\textbf {B}}}| \, |Z| \; \Vert \Psi \Vert _2 \leqslant C\, h^2 \; \Vert \Psi \Vert _{H_{\text {mag}}^1(Q_h)} \; (1 + |Z|), \end{aligned}$$

which subsequently proves

4.69 $$\begin{aligned}&\Vert \Pi (L_{T, {{\textbf {A}}}}\Delta - {\widetilde{L}}_{T, {{\textbf {A}}}}\Delta )\Vert _2^2 \leqslant C \, h^2 \; \Vert \Psi \Vert _{H_{\text {mag}}^1(Q_h)}^2 \nonumber \\&\quad \times \int _{{\mathbb {R}}^3} \text {d}r \Bigl ( \; \Bigl | \iint _{{\mathbb {R}}^3 \times {\mathbb {R}}^3} \text {d}Z\text {d}s \; h \, (1+ |Z|) \; \mathop {\mathrm {ess \, sup}}\limits _{X\in {\mathbb {R}}^3} |(k_{T, {{\textbf {A}}}} - {\widetilde{k}}_{T, {{\textbf {A}}}})(X, Z, r,s)| |V\alpha _*(s)|\Bigr |^2 \nonumber \\&\quad + \Bigl | \iint _{{\mathbb {R}}^3 \times {\mathbb {R}}^3} \text {d}Z\text {d}s \; \mathop {\mathrm {ess \, sup}}\limits _{X\in {\mathbb {R}}^3} |(\nabla _X k_{T, {{\textbf {A}}}} - \nabla _X \widetilde{k}_{T, {{\textbf {A}}}})(X, Z, r, s)| \, |V\alpha _*(s)|\Bigr |^2 \; \Bigr ). \end{aligned}$$

We claim that

4.70 $$\begin{aligned} |\nabla _X {\widetilde{\Phi }}_{{\textbf {A}}}(X, Z, r, s) | \leqslant C \, \Vert D{{\textbf {A}}}\Vert _\infty \Bigl ( \bigl | Z + \frac{r-s}{2}\bigr | + \bigl | Z - \frac{r-s}{2}\bigr |\Bigr ) \end{aligned}$$

holds, where $D{{\textbf {A}}}$ denotes the Jacobi matrix of ${{\textbf {A}}}$. To see this, we use Lemma 4.5 and compute

$$\begin{aligned} \nabla _X {\widetilde{\Phi }}_{{\textbf {A}}}(X, Z, r, s)&= {{\textbf {A}}}\bigl ( X + Z + \frac{s}{2}\bigr ) - {{\textbf {A}}}\bigl ( X + \frac{r}{2}\bigr ) + {{\textbf {A}}}\bigl (X + Z - \frac{s}{2}\bigr ) - {{\textbf {A}}}\bigl ( X - \frac{r}{2}\bigr ) \\&\quad + {\widetilde{{{\textbf {A}}}}}\bigl ( X + \frac{r}{2}, X + Z + \frac{s}{2}\bigr ) - {\widetilde{{{\textbf {A}}}}} \bigl ( X + Z + \frac{s}{2}, X +\frac{r}{2}\bigr ) \\&\quad + {\widetilde{{{\textbf {A}}}}}\bigl ( X - \frac{r}{2}, X + Z - \frac{s}{2}\bigr ) - {\widetilde{{{\textbf {A}}}}} \bigl ( X + Z - \frac{s}{2}, X - \frac{r}{2}\bigr ). \end{aligned}$$

The claim is a direct computation of this equality and a first order Taylor approximation. In combination, Proposition 4.2, (4.63), and (4.70) imply that, for *h* small enough,

$$\begin{aligned} \int _{{\mathbb {R}}^3} \text {d}Z \; \mathop {\mathrm {ess \, sup}}\limits _{X\in {\mathbb {R}}^3} |(\nabla _X k_{T, {{\textbf {A}}}} - \nabla _X {\widetilde{k}}_{T, {{\textbf {A}}}})(X, Z, r, s)|&\leqslant \bigl ( G_{T, h} + F_{T, h}^1\bigr ) (r-s). \end{aligned}$$

The functions $F_{T, h}^1$ and $G_{T, h}$ are defined in (4.53) and (4.54), respectively. A similar argument that uses $|Z| \leqslant | Z + \frac{r}{2}| + | Z - \frac{r}{2}|$ shows

$$\begin{aligned} \int _{{\mathbb {R}}^3 } \text {d}Z \; (1+ |Z|) \; \mathop {\mathrm {ess \, sup}}\limits _{X\in {\mathbb {R}}^3} |(k_{T, {{\textbf {A}}}} - {\widetilde{k}}_{T, {{\textbf {A}}}})(X, Z, r,s)| \leqslant F_{T, h}^1(r-s). \end{aligned}$$

When we insert these two bounds into (4.69), this proves (4.66). It remains to consider the third term on the right side of (4.58).

We claim that it satisfies

4.71 $$\begin{aligned} \Vert {\widetilde{\pi }} (L_{T, {{\textbf {A}}}}\Delta - {\widetilde{L}}_{T, {{\textbf {A}}}} \Delta )\Vert _2^2&\leqslant C \; \Vert \Psi \Vert _2^2 \; \Vert (F_{T, h}^1 + G_{T, h}) * | \ |\cdot | V\alpha _*| \, \Vert _2^2 \nonumber \\&\leqslant C \, h^8\; \Vert \ |\cdot | \ V\alpha _*\Vert _2^2 \; \Vert \Psi \Vert _{H_{\text {mag}}^1(Q_h)}^2. \end{aligned}$$

The second inequality follows from Young’s inequality, (2.7), and (4.55). To see that the first inequality holds, we first estimate

4.72 $$\begin{aligned} \Vert {\widetilde{\pi }} (L_{T, {{\textbf {A}}}}\Delta&- {\widetilde{L}}_{T, {{\textbf {A}}}}\Delta )\Vert _2^2 \leqslant 4 \;\Vert \Psi \Vert _2^2 \nonumber \\&\times \int _{{\mathbb {R}}^3} \text {d}r \, \Bigl | \iint _{{\mathbb {R}}^3 \times {\mathbb {R}}^3} \text {d}Z\text {d}s \; \mathop {\mathrm {ess \, sup}}\limits _{X\in {\mathbb {R}}^3} |({\widetilde{\pi }} k_{T, {{\textbf {A}}}} - {\widetilde{\pi }}{\widetilde{k}}_{T, {{\textbf {A}}}} )(X, Z, r, s)| \;|V\alpha _*(s)| \Bigr |^2. \end{aligned}$$

We start by noting that

$$\begin{aligned}&{\widetilde{\pi }} k_{T, {{\textbf {A}}}}(X, Z, r, s) - {\widetilde{\pi }} {\widetilde{k}}_{T, {{\textbf {A}}}}(X, Z, r, s) \\&= \text {e}^{\text {i} {\widetilde{\Phi }}_{{{\textbf {A}}}}(X,Z,r,s)} \bigl (-\text {i}\nabla _r + \frac{1}{4} {{\textbf {B}}}\wedge r + \nabla _r {\widetilde{\Phi }}_{{{\textbf {A}}}}(X,Z,r,s) \bigr ) \frac{2}{\beta }\sum _{n\in {\mathbb {Z}}} ( k_{T, {{\textbf {A}}}}^n(X, Z, r, s) - {\widetilde{k}}_T^n(Z, r-s) ) \end{aligned}$$

and

$$\begin{aligned} \nabla _r {\widetilde{\Phi }}_{{{\textbf {A}}}}(X, Z, r, s)&= -\frac{1}{2} \bigl [ {{\textbf {A}}}\bigl ( X + \frac{r}{2}\bigr ) - {{\textbf {A}}}\bigl ( X - \frac{r}{2}\bigr ) \bigr ] \\&\quad + \frac{1}{2} \bigl [ {\widetilde{{{\textbf {A}}}}}\bigl ( X + \frac{r}{2}, X + Z + \frac{s}{2}\bigr ) - {\widetilde{{{\textbf {A}}}}} \bigl ( X - \frac{r}{2}, X + Z - \frac{s}{2}\bigr )\bigr ], \end{aligned}$$

which follows from Lemma 4.5. We combine these two identities and estimate

4.73 $$\begin{aligned} |{\widetilde{\pi }} k_{T, {{\textbf {A}}}} - {\widetilde{\pi }} {\widetilde{k}}_{T, {{\textbf {A}}}}| \leqslant&\frac{2}{\beta }\sum _{n\in {\mathbb {Z}}} |\nabla _r k_{T, {{\textbf {A}}}}^n - \nabla _r k_T^n| + |k_{T, {{\textbf {A}}}} - {\widetilde{k}}_{T, {{\textbf {A}}}}| \left( \frac{| {{\textbf {B}}}|}{4} + h^2 \Vert D {{\textbf {A}}}\Vert _{\infty } \right) \nonumber \\&\quad \times \left( | r-s| + |s| + \left| Z + \frac{r-s}{2} \right| + \left| Z - \frac{r-s}{2} \right| \right) . \end{aligned}$$

We apply

4.74 $$\begin{aligned} |r-s|^a&= \bigl | \frac{r-s}{2} + Z + \frac{r-s}{2} - Z\bigr |^a \leqslant 2^{(a-1)_+} \Bigl ( \bigl | Z - \frac{r-s}{2}\bigr |^a + \bigl | Z + \frac{r-s}{2}\bigr |^a\Bigr ), \end{aligned}$$

which holds for $a \geqslant 0$, with $a=1$ to bound $|r-s|$ in (4.73). When we put (4.73) and (4.74) together, and additionally use Lemma 4.2, (4.63) and (4.73), we find

4.75 $$\begin{aligned} \int _{{\mathbb {R}}^3} \text {d}Z \; \mathop {\mathrm {ess \, sup}}\limits _{X\in {\mathbb {R}}^3} |({\widetilde{\pi }} k_{T, {{\textbf {A}}}} - {\widetilde{\pi }}{\widetilde{k}}_{T, {{\textbf {A}}}}) (X, Z, r, s)| \leqslant C \bigl (F_{T, h}^1 + G_{T, h}\bigr )(r-s) (1+|s|). \end{aligned}$$

Finally, (4.75) and (4.72) imply (4.71), which finishes the proof of Proposition 4.9. $\square$

**4.5.3.2 The operator **$${\widetilde{M}}_{T,{{\textbf {A}}}}$$.

We define ${\widetilde{M}}_{T,{{\textbf {A}}}}$ by

4.76 $$\begin{aligned} {\widetilde{M}}_{T,{{\textbf {A}}}}\alpha (X,r) {:}{=}\iint _{{\mathbb {R}}^3 \times {\mathbb {R}}^3} \text {d}Z\text {d}s \; k_T(Z , r-s) \, \text {e}^{-\text {i}\frac{r-s}{4} \cdot D{{\textbf {A}}}_h (X)(r+s)} \, (\cos (Z \cdot \Pi _{{{\textbf {A}}}_h}) \alpha ) (X,s), \end{aligned}$$

where

4.77 $$\begin{aligned} k_T(Z, r) {:}{=}\frac{2}{\beta } \sum _{n\in {\mathbb {Z}}} g_0^{\text {i}\omega _n}\bigl (Z - \frac{r}{2} \bigr ) \, g_0^{-\text {i}\omega _n}\bigl ( Z + \frac{r}{2} \bigr ). \end{aligned}$$

By $(D{{\textbf {A}}})_{ij} {:}{=}\partial _j {{\textbf {A}}}_i$ we denote the Jacobi matrix of ${{\textbf {A}}}$. Here and in the following we use the notation that $D{{\textbf {A}}}_h (X)(r+s)$ denotes the matrix $D{{\textbf {A}}}_h (X)$ applied to $r+s$. Accordingly, $\frac{r-s}{4} \cdot D{{\textbf {A}}}_h (X)(r+s)$ is the inner product of this vector with $\frac{r-s}{4}$.

The following proposition allows us to replace ${\widetilde{L}}_{T, {{\textbf {A}}}}$ by ${\widetilde{M}}_{T, {{\textbf {A}}}}$ in our computations.

##### Proposition 4.11

Assume that ${{\textbf {A}}}= {{\textbf {A}}}_{e_3} + A$ with $A \in W^{2,\infty }({\mathbb {R}}^3,{\mathbb {R}}^3)$ periodic, let $|\cdot |^k V\alpha _*\in L^2({\mathbb {R}}^3)$ for $k \in \{ 0,1,2 \}$, $\Psi \in H_{\text {mag}}^1(Q_h)$, and denote $\Delta \equiv \Delta _\Psi$ as in (3.1). For any $T_0 > 0$ there is $h_0>0$ such that for any $0 < h \leqslant h_0$ and any $T\geqslant T_0$ we have

4.78 $$\begin{aligned} \Vert {\widetilde{L}}_{T, {{\textbf {A}}}}\Delta - {\widetilde{M}}_{T,{{\textbf {A}}}}\Delta \Vert _{H^1(Q_h \times {\mathbb {R}}_{{\text {s}}}^3)}^2&\leqslant C\; h^8 \; \max _{k =0,1,2} \Vert \, |\cdot |^k V\alpha _*\Vert _2^2 \;\Vert \Psi \Vert _{H_{\text {mag}}^1(Q_h)}^2. \end{aligned}$$

Before we give the proof of Proposition 4.11, we state and prove two preparatory lemmas. The first lemma allows us to extract the term $\Phi _{2A_h}(X, X + Z)$ and to replace $\text {e}^{\text {i}Z \cdot (-\text {i}\nabla )}$ by $\text {e}^{\text {i}Z \cdot \Pi _{{{\textbf {A}}}_h}}$ in the definition of ${\widetilde{L}}_{T, {{\textbf {A}}}}$. In [15, 26], where only the constant magnetic field is present, this approximation holds as an algebraic identity. A version of the first bound in (4.79) has, in case of a periodic vector potential, been proved in [18]. In this reference *Z* does not appear on the right side, which is why this bound appears to be wrong.

##### Lemma 4.12

Assume that $A\in W^{2, \infty }({\mathbb {R}}^3; {\mathbb {R}}^3)$. We then have

4.79 $$\begin{aligned} \sup _{X\in {\mathbb {R}}^3} \bigl | {\widetilde{\Phi }}_A(X, Z, r, s) - \Phi _{2A}&(X,X+Z) + \frac{1}{4} (r-s)\cdot D A(X)(r+s)\bigr | \nonumber \\&\quad \leqslant C \; \Vert D^2A\Vert _\infty \; \bigl ( |Z| + |r-s|\bigr ) \bigl ( |s|^2 + |r-s|^2\bigr ), \end{aligned}$$

where $\Phi _A$ is defined in (4.2) and ${\widetilde{\Phi }}_A$ is defined in (4.47). We also have

4.80 $$\begin{aligned} \sup _{X\in {\mathbb {R}}^3} \bigl | \nabla _X {\widetilde{\Phi }}_A(X, Z, r, s)&- \nabla _X \Phi _{2A} (X, X + Z) \bigr | \nonumber \\&\leqslant C \; \Vert D^2A\Vert _\infty \; \bigl ( |Z| + |r-s|\bigr ) \bigl (|s| + |r-s|\bigr ) \end{aligned}$$

as well as

4.81 $$\begin{aligned} \sup _{X\in {\mathbb {R}}^3} \bigl | \nabla _X (r-s) \cdot DA(X) \cdot (r+s) \bigr | \leqslant C \, \Vert D^2A\Vert _\infty \, |r-s| \, \bigl ( |s| + |r-s|\bigr ) \end{aligned}$$

and

4.82 $$\begin{aligned} \sup _{X\in {\mathbb {R}}^3} \bigl | \nabla _r {\widetilde{\Phi }}_A(X, Z, r, s)&+ \frac{1}{4} \nabla _r (r-s)\cdot D A(X)(r+s) \bigr | \nonumber \\&\quad \leqslant C\, \Vert D^2A\Vert _\infty \, \bigl ( |s|^2 + |r-s|^2 + |Z|^2\bigr ). \end{aligned}$$

The same bounds hold if $A$ is replaced by ${{\textbf {A}}}_{e_3} + A$.

##### Proof of Lemma 4.12

We use the notation $\zeta _X^r {:}{=}X + \frac{r}{2}$ and start by writing

4.83 $$\begin{aligned} {\widetilde{\Phi }}_A(X, Z, r, s)&= \int _0^1 \text {d}t\; \bigl [A\bigl ( \zeta _{X + Z - tZ}^{s + t(r-s)} \bigr ) + A\bigl (\zeta _{X + Z - tZ}^{-s - t(r-s)}\bigr ) \bigr ] \cdot Z \nonumber \\&\quad - \int _0^1 \text {d}t\; \bigl [A\bigl (\zeta _{X + Z - tZ}^{s + t(r-s)}\bigr ) - A\bigl ( \zeta _{X + Z - tZ}^{-s - t(r-s)} \bigr ) \bigr ] \cdot \frac{r-s}{2}. \end{aligned}$$

A second order Taylor expansion in the variable $\pm \frac{1}{2} (s + t(r-s))$ allows us to see that

4.84 $$\begin{aligned} \Bigl | A\bigl (X + Z- tZ \pm \frac{s + t(r-s)}{2}\bigr ) - A(X + Z-tZ)&\mp \frac{1}{2} DA(X + Z-tZ) (s + t(r-s)) \Bigr | \nonumber \\&\leqslant C\; \Vert D^2A\Vert _\infty \; \bigl (|s|^2 +|r-s|^2\bigr ). \end{aligned}$$

For the first term on the right side of (4.83), this implies

4.85 $$\begin{aligned} \Bigl | \int _0^1 \text {d}t\; \bigl [A\bigl ( \zeta _{X + Z - tZ}^{s + t(r-s)} \bigr ) + A\bigl ( \zeta _{X + Z - tZ}^{-s - t(r-s)} \bigr ) \bigr ] \cdot&Z -\Phi _{2A}(X,X+Z) \Bigr | \nonumber \\&\quad \leqslant C \, \Vert D^2A\Vert _\infty \, |Z| \, \bigl (|s|^2 + |r-s|^2\bigr ), \end{aligned}$$

and we find

4.86 $$\begin{aligned}&\Bigl | - \int _0^1 \text {d}t\; \bigl [A\bigl ( \zeta _{X + Z - tZ}^{s + t(r-s)} \bigr ) - A\bigl (\zeta _{X + Z - tZ}^{-s - t(r-s)}\bigr ) \bigr ] \cdot \frac{r-s}{2}+ \frac{1}{4} (r-s) \cdot DA(X) (r+s) \Bigr | \nonumber \\&\quad \leqslant \Bigl | -\frac{r-s}{2} \cdot \int _0^1 \text {d}t \; \bigl [ DA(X + Z - tZ) - DA(X) \bigr ](s + t(r-s)) \Bigr | \nonumber \\&\quad \qquad \qquad \qquad + C\, \Vert D^2A\Vert _\infty \, \bigl ( |s|^2 + |r-s|^2\bigr ) \, |r-s| \nonumber \\&\quad \leqslant C\, \Vert D^2A\Vert _\infty \, |r-s| \, \bigl [|s|^2 + |r-s|^2 + \bigl (|s| + |r-s|\bigr ) |Z| \bigr ] \end{aligned}$$

for the second term. Adding up (4.85) and (4.86) proves (4.79). The proof of (4.80) results from a first order Taylor expansion and that of (4.81) is a straightforward computation. It remains to prove (4.82).

When we differentiate (4.83) with respect to *r* this yields

4.87 $$\begin{aligned} \nabla _r {\widetilde{\Phi }}_A(X, Z, r, s)&= \int _0^1 \text {d}t\; \frac{t}{2} \ Z \cdot \bigl [DA\bigl ( \zeta _{X + Z - tZ}^{s + t(r-s)} \bigr ) - DA\bigl (\zeta _{X + Z - tZ}^{-s - t(r-s)}\bigr ) \bigr ] \nonumber \\&\quad - \int _0^1 \text {d}t\; \frac{t}{2} \ \frac{r-s}{2} \cdot \bigl [DA\bigl (\zeta _{X + Z - tZ}^{s + t(r-s)} \bigr ) + DA\bigl (\zeta _{X + Z - tZ}^{-s - t(r-s)} \bigr ) \bigr ] \nonumber \\&\quad - \frac{1}{2} \int _0^1 \text {d}t\; \bigl [A\bigl (\zeta _{X + Z - tZ}^{s + t(r-s)} \bigr ) - A\bigl (\zeta _{X + Z - tZ}^{-s - t(r-s)} \bigr ) \bigr ]. \end{aligned}$$

A first order Taylor expansion shows that the absolute value of the first term on the right side of (4.87) is bounded by $C\Vert D^2A\Vert _\infty (|s| + |r-s|) |Z|$. We also note that

4.88 $$\begin{aligned} \frac{1}{4} \nabla _r (r - s) \cdot DA(X) (r + s) = \frac{1}{4} (r-s) \cdot DA(X) + \frac{1}{4} DA(X) (r+s). \end{aligned}$$

The second term on the right side of (4.87) obeys

4.89 $$\begin{aligned}&\Bigl | \int _0^1 \text {d}t\; t \; \frac{r-s}{4} \cdot \bigl [DA\bigl (\zeta _{X + Z - tZ}^{s + t(r-s)} \bigr ) + DA\bigl (\zeta _{X + Z - tZ}^{-s - t(r-s)} \bigr ) \bigr ] - \frac{1}{4} (r-s) \cdot DA(X) \bigr | \nonumber \\&\quad \leqslant \Bigl | \int _0^1 \text {d}t \; \frac{t}{2} \; (r-s) \cdot \bigl [ DA(X + Z - tZ) - DA(X) \bigr ] \Bigr | \nonumber \\&\qquad + C \, \Vert D^2A\Vert _\infty \bigl ( |s| + |r-s|\bigr ) |r-s| \nonumber \\&\quad \leqslant C \, \Vert D^2A\Vert _\infty \, \bigl [ |r-s| \, |Z| + \bigl ( |s| + |r-s|\bigr ) |r-s|\bigr ] . \end{aligned}$$

For the third term on the right side of (4.87), we use the bound

$$\begin{aligned} \Bigl | \frac{1}{2} \int _0^1 \text {d}t\; \bigl [A\bigl (\zeta _{X + Z - tZ}^{s + t(r-s)} \bigr ) - A\bigl (\zeta _{X + Z - tZ}^{-s - t(r-s)} \bigr ) \bigr ] - \frac{1}{4} DA(X) (r+ s)\Bigr | \leqslant {\mathcal {T}}_+ + {\mathcal {T}}_- + {\mathcal {T}}, \end{aligned}$$

where

$$\begin{aligned} {\mathcal {T}}_\pm&{:}{=}\Bigl | \frac{1}{2} \int _0^1 \text {d}t \; \bigl [ A\bigl ( \zeta _{X + Z - tZ}^{\pm s \pm t(r-s)}\bigr ) {\mp } A(X + Z - tZ) - \frac{1}{2} DA(X + Z- tZ) (s+ t(r-s)) \bigr ]\Bigr | \end{aligned}$$

and

$$\begin{aligned} {\mathcal {T}}&{:}{=}\Bigl | \frac{1}{2} \int _0^1 \text {d}t \; DA(X + Z- tZ) (s + t(r-s)) - \frac{1}{4} DA(X) (r+s)\Bigr |. \end{aligned}$$

By (4.84), we have

$$\begin{aligned} {\mathcal {T}}_\pm&\leqslant C \, \Vert D^2 A\Vert _\infty \, \bigl ( |s|^2 + |r-s|^2\bigr ),&{\mathcal {T}}&\leqslant C\, \Vert D^2A\Vert _\infty \, \bigl ( |s| + |r-s|\bigr ) \, |Z|. \end{aligned}$$

In combination with (4.89), these considerations imply (4.82). The proof for ${{\textbf {A}}}_{e_3} + A$ is literally the same. $\square$

The next lemma is a substitute for the identity

4.90 $$\begin{aligned} \text {e}^{\text {i}{{\textbf {B}}}\cdot (X \wedge Z)} \text {e}^{\text {i}Z \cdot P_X} = \text {e}^{\text {i}Z \cdot \Pi _X} \end{aligned}$$

in the case of a general magnetic field. It holds because ${{\textbf {B}}}\cdot (X\wedge Z) = Z \cdot ({{\textbf {B}}}\wedge X)$ and the latter commutes with $Z \cdot \Pi _X$. Here, we used the notations $P = -\text {i}\nabla _X$ for the momentum operator and $\Pi _X = -\text {i}\nabla _X + 2 {{\textbf {A}}}_{{{\textbf {B}}}}(X)$ for its magnetic counterpart.

##### Lemma 4.13

Assume that $A\in L^{\infty }({\mathbb {R}}^3,{\mathbb {R}}^3)$ is a periodic function. Then

4.91 $$\begin{aligned} \text {e}^{\text {i}\Phi _{2A_h}(X , X +Z)} \, \text {e}^{ \text {i}Z\cdot \Pi _X } = \text {e}^{ \text {i}Z\cdot \Pi _{{{\textbf {A}}}_h} }, \end{aligned}$$

where $\Pi _{{{\textbf {A}}}_h} = P_X + 2 {{\textbf {A}}}_h(X)$ is understood to act on the *X* coordinate.

The above lemma is a consequence of the following more abstract proposition, whose proof can be found in [37, p. 290]. For the sake of completeness we repeat it here.

##### Proposition 4.14

Let ${\mathcal {H}}$ be a separable Hilbert space, let $P:{\mathcal {D}}(P)\rightarrow {\mathcal {H}}$ be a densely defined self-adjoint operator, and assume that *Q* is bounded and self-adjoint. Assume further that $[\text {e}^{\text {i}tP} \, Q \, \text {e}^{-\text {i}tP}, \text {e}^{\text {i}sP} \, Q \, \text {e}^{-\text {i}sP} ] =0$ for every $t,s\in [0,1]$. Then, we have

$$\begin{aligned} \exp \Bigl (\text {i}\int _0^1\text {d}t\; \text {e}^{\text {i}tP} \, Q\, \text {e}^{-\text {i}tP}\Bigr ) \, \text {e}^{\text {i}P} = \text {e}^{\text {i}(P + Q)}. \end{aligned}$$

##### Proof

For $s\in {\mathbb {R}}$ we define $Q(s) {:}{=}\text {e}^{\text {i}sP} \, Q \, \text {e}^{-\text {i}sP}$ and $W(s) {:}{=}\text {e}^{\text {i}s(P+Q)} \, \text {e}^{-\text {i}sP}$. On ${\mathcal {D}}(P)$, we may differentiate *W* to get

$$\begin{aligned} -\text {i}W'(s) = \text {e}^{\text {i}s(P+Q)} (P+Q) \, \text {e}^{-\text {i}sP} - \text {e}^{\text {i}s(P+Q)} \, P \, \text {e}^{-\text {i}sP} = \text {e}^{\text {i}s(P+Q)} \, Q \, \text {e}^{-\text {i}sP} = W(s) \, Q(s). \end{aligned}$$

Using that *Q* is bounded we conclude that this identity also holds on ${\mathcal {H}}$. Hence, *W* satisfies the linear differential equation $W'(s) = \text {i}W(s) \, Q(s)$. Since by assumption $[Q(s),Q(t)] = 0$ holds for all $s,t \in [0,1]$, we conclude that the unique solution to this equation can be written as

$$\begin{aligned} {\widetilde{W}}(s) {:}{=}\exp \Bigl (\text {i}\int _0^s \text {d}t\; Q(t)\Bigr ), \end{aligned}$$

and hence

$$\begin{aligned} \exp \Bigl (\text {i}\int _0^s \text {d}t\; Q(t)\Bigr ) = \text {e}^{\text {i}s(P+Q)}\text {e}^{-\text {i}sP}. \end{aligned}$$

With the choice $s =1$ this equation proves the claim. $\square$

##### Proof of Lemma 4.13

We first show that

4.92 $$\begin{aligned} \text {e}^{ \text {i}\Phi _{2A_h}(X, X+Z) } \, \text {e}^{ \text {i}Z \cdot P_X } = \text {e}^{ \text {i}Z \cdot (P_X + 2A_h(X)) } \end{aligned}$$

holds. To that end, we apply Proposition 4.14 with the choices $P = Z\cdot P_X$, where $P_X = -\text {i}\nabla _X$, and $Q = 2 Z\cdot A_h(X)$ and find

$$\begin{aligned} \exp \Bigl (\text {i}\int _0^1\text {d}t\; \text {e}^{\text {i}t Z\cdot P_X} \, 2 Z\cdot A_h(X) \, \text {e}^{-\text {i}t Z \cdot P_X}\Bigr ) \, \text {e}^{\text {i}Z\cdot P_X} = \text {e}^{\text {i}(Z \cdot (P_X + 2 A(X)))}. \end{aligned}$$

It remains to compute the integral in the exponential, which reads

$$\begin{aligned} \int _0^1\text {d}t\; \text {e}^{\text {i}t Z\cdot P_X} \, 2 Z\cdot A_h(X) \, \text {e}^{-\text {i}t Z \cdot P_X} = 2 \int _0^1\text {d}t\; Z\cdot A_h(X+tZ) = \Phi _{2A_h}(X,X+Z). \end{aligned}$$

To obtain the last equality we applied the coordinate transformation $t\mapsto 1 -t$. In combination, these considerations prove (4.92).

Next, we use (4.92) and (4.90) to see that

$$\begin{aligned} \text {e}^{\text {i}\Phi _{2A_h}(X , X +Z)} \, \text {e}^{ \text {i}Z\cdot \Pi _X }&= \text {e}^{ \text {i}\Phi _{2A_h}(X, X+Z) } \, \text {e}^{ \text {i}Z \cdot P_X } \; \text {e}^{\text {i}{{\textbf {B}}}\cdot (X \wedge Z)} = \text {e}^{ \text {i}Z \cdot (P_X + 2A_h(X)) } \; \text {e}^{\text {i}{{\textbf {B}}}\cdot (X \wedge Z)} \\&= \text {e}^{ \text {i}Z\cdot \Pi _{{{\textbf {A}}}_h} } \end{aligned}$$

holds. This proves Lemma 4.13. $\square$

For $a\in {\mathbb {N}}_0$ we define the functions

4.93 $$\begin{aligned} F_T^{a} {:}{=}\frac{2}{\beta }\sum _{n\in {\mathbb {Z}}} \sum _{m=0}^a \sum _{b = 0}^m \left( {\begin{array}{c}m\\ b\end{array}}\right) \; \bigl (|\cdot |^{b}\, |g_0^{\text {i}\omega _n}|\bigr ) * \bigl (|\cdot |^{m-b} \, |g_0^{-\text {i}\omega _n}| \bigr ) \end{aligned}$$

and

4.94 $$\begin{aligned} G_T^a&{:}{=}&\smash {\frac{2}{\beta }\sum _{n\in {\mathbb {Z}}} \sum _{m=0}^a \sum _{b=0}^m \left( {\begin{array}{c}m\\ b\end{array}}\right) } \; \bigl ( |\cdot |^b \, |\nabla g_0^{\text {i}\omega _n}| \bigr ) * \bigl ( |\cdot |^{m-b} \, |g_0^{-\text {i}\omega _n}|\bigr ) \nonumber \\{} & {} \quad \qquad \qquad \qquad + \bigl ( |\cdot |^b \, |g_0^{\text {i}\omega _n}| \bigr ) * \bigl ( |\cdot |^{m-b} \, |\nabla g_0^{-\text {i}\omega _n}| \bigr ), \end{aligned}$$

which play a prominent role in the proof of Proposition 4.11. An application of Lemma 4.4, (4.56), and (4.57) shows that for $T \geqslant T_0 > 0$ and $a\in {\mathbb {N}}_0$, we have

4.95 $$\begin{aligned} \Vert F_{T}^a\Vert _1 + \Vert G_T^a \Vert _1&\leqslant C_{a}. \end{aligned}$$

We are now prepared to give the proof of Proposition 4.11.

##### Proof of Proposition 4.11

We use (4.90) and $\Phi _{{{\textbf {A}}}_{{{\textbf {B}}}}}(x,y)= \frac{{\textbf {B}}}{2} \cdot ( x \wedge y)$ to write the operator ${\widetilde{L}}_{T, {{\textbf {A}}}}$ as

$$\begin{aligned} {\widetilde{L}}_{T, {{\textbf {A}}}} \alpha (X, r)&= \iint _{{\mathbb {R}}^3 \times {\mathbb {R}}^3} \text {d}Z \text {d}s \; \text {e}^{\text {i}\frac{{{\textbf {B}}}}{4} \cdot (r\wedge s)} k_T(Z, r - s) \, \text {e}^{\text {i}{\widetilde{\Phi }}_{A_h}(X, Z, r, s)} \, (\text {e}^{\text {i}Z \cdot \Pi } \alpha )(X, s), \end{aligned}$$

where $\Pi = -\text {i}\nabla + 2 {{\textbf {A}}}_{{{\textbf {B}}}}$. With the identities

$$\begin{aligned} - \frac{r-s}{4} \cdot D {{\textbf {A}}}_{{{\textbf {B}}}} (r + s)&= \frac{{{\textbf {B}}}}{4} \cdot (r\wedge s),&\Phi _{2{{\textbf {A}}}_{{{\textbf {B}}}}}(X, X + Z)&= Z \cdot ({{\textbf {B}}}\wedge X), \end{aligned}$$

and (4.47) we check that

$$\begin{aligned} {\widetilde{\Phi }}_{{{\textbf {A}}}_{{{\textbf {B}}}}} (X, Z, r, s)&= - \frac{r-s}{4} \cdot D{{\textbf {A}}}_{{{\textbf {B}}}}(X) (r+s) + \Phi _{2{{\textbf {A}}}_{{{\textbf {B}}}}}(X, X + Z). \end{aligned}$$

In combination with Lemma 4.13 and the fact that the integrand in the definition of ${\widetilde{M}}_{T, {{\textbf {A}}}}$ is an even function of *Z*, this allows us to write $\widetilde{M}_{T, {{\textbf {A}}}}$ as

$$\begin{aligned} {\widetilde{M}}_{T, {{\textbf {A}}}} \alpha (X, r)= & {} \smash {\iint _{{\mathbb {R}}^3 \times {\mathbb {R}}^3}} \text {d}Z \text {d}s \; \text {e}^{\text {i}\frac{{{\textbf {B}}}}{4} \cdot (r\wedge s)} k_T(Z, r-s) \, \text {e}^{\text {i}\Phi _{2A_h} (X, X+Z)} \, \text {e}^{-\text {i}\frac{r-s}{4} \cdot DA_h(X) (r + s)} \\\\{} & {} \quad \times (\text {e}^{\text {i}Z \cdot \Pi } \alpha )(X, s), \end{aligned}$$

and, consequently,

4.96 $$\begin{aligned} \bigl ({\widetilde{L}}_{T, {{\textbf {A}}}} \Delta - {\widetilde{M}}_{T, {{\textbf {A}}}} \Delta \bigr ) (X, r)&= -2 \iint _{{\mathbb {R}}^3 \times {\mathbb {R}}^3} \text {d}Z \text {d}s \; \text {e}^{\text {i}\frac{{{\textbf {B}}}}{4} \cdot (r\wedge s)} k_T(Z , r-s) \, V\alpha _*(s) \, (\text {e}^{\text {i}Z \cdot \Pi } \Psi )(X)\nonumber \\&\quad \times \bigl [ \text {e}^{\text {i}{\widetilde{\Phi }}_{A_h}(X, Z, r, s)} - \text {e}^{\text {i}\Phi _{2A_h}(X, X + Z)}\text {e}^{-\text {i}\frac{r-s}{4} \cdot DA_h(X)(r + s)} \bigr ]. \end{aligned}$$

We claim that

4.97 $$\begin{aligned} \Vert {\widetilde{L}}_{T, {{\textbf {A}}}} \Delta - {\widetilde{M}}_{T, {{\textbf {A}}}} \Delta \Vert _2^2&\leqslant C \, \Vert \Psi \Vert _2^2 \; \Vert D^2A_h\Vert _\infty ^2 \; \Vert F_T^3 * |V\alpha _*| + F_T^1 * |\cdot |^2\, |V\alpha _*| \, \Vert _2^2 \nonumber \\&\leqslant C\; h^8 \; \max _{k =0,1,2} \Vert \, |\cdot |^k V\alpha _*\Vert _2^2 \;\Vert \Psi \Vert _{H_{\text {mag}}^1(Q_h)}^2. \end{aligned}$$

The second bound in the above equation follows from Young’s inequality, (2.7), and (4.95). To prove the first bound in (4.97), we use (4.61) and find

4.98 $$\begin{aligned} \Vert {\widetilde{L}}_{T, {{\textbf {A}}}} \Delta - {\widetilde{M}}_{T, {{\textbf {A}}}} \Delta \Vert _2^2&\leqslant 4 \, \Vert \Psi \Vert _2^2 \int _{{\mathbb {R}}^3} \text {d}r \, \Bigl | \iint _{{\mathbb {R}}^3 \times {\mathbb {R}}^3} \text {d}Z \text {d}s \; |k_T(Z, r-s)| \, |V\alpha _*(s)| \nonumber \\&\quad \times \sup _{X\in {\mathbb {R}}^3} \bigl | \text {e}^{\text {i}\widetilde{\Phi }_{A_h}(X, Z, r, s) - \text {i}\Phi _{2A_h}(X, X+ Z) + \text {i}\frac{r-s}{4} \cdot DA_h(X) (r + s)} -1\bigr | \Bigr |^2. \end{aligned}$$

Furthermore, an application of Lemma 4.12 implies

4.99 $$\begin{aligned} \int _{{\mathbb {R}}^3} \text {d}Z \; |Z|^a \; |k_T(Z, r-s)| \,&\sup _{X\in {\mathbb {R}}^3} \bigl | \text {e}^{\text {i}{\widetilde{\Phi }}_{A}(X, Z, r, s) - \text {i}\Phi _{2A}(X, X+ Z) + \text {i}\frac{r-s}{4} \cdot DA(X) (r + s)} -1\bigr | \nonumber \\&\quad \leqslant C \; \Vert D^2A\Vert _\infty \bigl [ F_T^{3+a}(r-s) + F_T^{1+a}(r-s)\; |s|^2\bigr ] \end{aligned}$$

with $F_T^a$ in (4.93). We need this bound here only for the case $a=0$ but state it for general $a\geqslant 0$ for later reference. In combination with (4.98), this proves (4.97).

We claim that the term involving $\Pi = -\text {i}\nabla + 2 {{\textbf {A}}}_{{{\textbf {B}}}}$, which is understood to act on the center-of-mass coordinate, is bounded by

4.100 $$\begin{aligned}&\Vert \Pi ({\widetilde{L}}_{T, {{\textbf {A}}}}\Delta - {\widetilde{M}}_{T, {{\textbf {A}}}}\Delta )\Vert _2^2 \leqslant C\, h^2 \, \Vert \Psi \Vert _{H_{\text {mag}}^1(Q_h)}^2 \, \Vert D^2 A_h\Vert _\infty ^2 \, \bigl ( 1 + \Vert DA_h\Vert _\infty ^2\bigr ) \nonumber \\&\quad \qquad \quad \times \bigl [ \Vert F_T^4 * |V\alpha _*|\, \Vert _2^2 + \Vert F_T^1 * |\cdot | \, |V\alpha _*| \, \Vert _2^2 + \Vert F_T^2* |\cdot |^2|V\alpha _*| \, \Vert _2^2\bigr ]. \end{aligned}$$

If this holds, the desired bound for this term follows from Young’s inequality and (4.95). To prove (4.100), we use (4.96) and argue as in the proof of (4.69) to see that

4.101 $$\begin{aligned}&\Vert \Pi ({\widetilde{L}}_{T, {{\textbf {A}}}}\Delta - {\widetilde{M}}_{T, {{\textbf {A}}}}\Delta )\Vert _2^2 \leqslant C \, h^2 \, \Vert \Psi \Vert _{H_{\text {mag}}^1(Q_h)}^2 \int _{{\mathbb {R}}^3} \text {d}r\, \Big ( \; \Bigl | \iint _{{\mathbb {R}}^3 \times {\mathbb {R}}^3} \text {d}Z \text {d}s \; \, |V\alpha _*(s)| \nonumber \\&\quad \times |k_T(Z, r-s)| \Bigl [ h\, (1 + |Z|)\, \sup _{X\in {\mathbb {R}}^3} \bigl | \text {e}^{\text {i}{\widetilde{\Phi }}_{A_h}(X, Z, r, s) - \text {i}\Phi _{2A_h}(X, X+ Z) + \text {i}\frac{r-s}{4} \cdot DA_h(X) (r + s)} -1\bigr | \nonumber \\&\quad \qquad \qquad + \sup _{X\in {\mathbb {R}}^3} \bigl | \nabla _X \text {e}^{\text {i}\widetilde{\Phi }_{A_h}(X, Z, r, s)} - \nabla _X \text {e}^{\text {i}\Phi _{2A_h}(X, X + Z)} \text {e}^{-\text {i}\frac{r-s}{4} \cdot DA_h(X)(r + s)} \bigr | \Bigr ] \Bigr |^2 \Big ). \end{aligned}$$

The difference of the phases involving a gradient can be estimated as

$$\begin{aligned}&\bigl | \nabla _X \text {e}^{\text {i}{\widetilde{\Phi }}_{A}(X, Z, r, s)} - \nabla _X \text {e}^{\text {i}\Phi _{2A}(X, X + Z)} \text {e}^{-\text {i}\frac{r-s}{4} \cdot DA(X)(r + s)} \bigr | \\&\quad \qquad \quad \leqslant \bigl | \text {e}^{\text {i}{\widetilde{\Phi }}_{A}(X, Z, r, s) - \text {i}\Phi _{2A}(X, X+Z) + \text {i}\frac{r-s}{4} \cdot DA(X) (r + s)} - 1\bigr | |\nabla _X {\widetilde{\Phi }}_{A}(X, Z, r, s)| \\&\qquad \qquad \quad + \bigl | \nabla _X {\widetilde{\Phi }}_{A}(X, Z, r, s) - \nabla _X \Phi _{2A}(X, X + Z) \bigr | + \bigl |\nabla _X (r-s) \cdot DA(X) (r+s)\bigr |, \end{aligned}$$

which, by (4.70) and Lemma 4.12, is bounded by

$$\begin{aligned}&C \, \Vert D^2A\Vert _\infty \, \bigl (1 + \Vert DA\Vert _\infty \bigr ) \\&\quad \times \bigl [ \bigl ( |s|^2 + |r-s|^2 \bigr ) \bigl ( 1+ |Z|^2 + |r-s|^2\bigr ) + \bigl ( |s| + |r-s|\bigr ) \bigl ( |Z| + |r-s|\bigr )\bigr ]. \end{aligned}$$

Accordingly,

$$\begin{aligned}&\int _{{\mathbb {R}}^3} \text {d}Z \; |k_T(Z, r-s)| \, \sup _{X\in {\mathbb {R}}^3} \bigl | \nabla _X \text {e}^{\text {i}{\widetilde{\Phi }}_{A}(X, Z, r, s)} - \nabla _X \text {e}^{\text {i}\Phi _{2A}(X, X + Z)} \text {e}^{-\text {i}\frac{r-s}{4} \cdot DA(X)(r + s)} \bigr | \\&\quad \leqslant C \, \Vert D^2A\Vert _\infty \bigl ( 1 + \Vert DA\Vert _\infty \bigr ) \\&\qquad \qquad \qquad \quad \times \bigl [ F_T^4(r-s) + F_T^1(r-s) \, |s| + F_T^2(r-s) \, |s|^2 \bigr ]. \end{aligned}$$

Using (4.99), we also find

$$\begin{aligned}&\int _{{\mathbb {R}}^3} \text {d}Z \; |k_T(Z, r-s)| \, h\, (1 + |Z|) \sup _{X\in {\mathbb {R}}^3} \bigl | \text {e}^{\text {i}{\widetilde{\Phi }}_{A}(X, Z, r, s) - \text {i}\Phi _{2A}(X, X+ Z) + \text {i}\frac{r-s}{4} \cdot DA(X) (r + s)} -1\bigr | \\&\quad \leqslant C \, \Vert D^2A\Vert _\infty \; \bigl [ F_T^4(r-s) + F_T^2(r-s) \, |s|^2 \bigr ]. \end{aligned}$$

In combination with (4.101), this proves (4.100).

We claim that the term involving ${\widetilde{\pi }} = -\text {i}\nabla + \frac{1}{2} {{\textbf {A}}}_{{{\textbf {B}}}}$, which is understood to act on the relative coordinate, is bounded by

4.102 $$\begin{aligned} \Vert {\widetilde{\pi }} ({\widetilde{L}}_{T, {{\textbf {A}}}} \Delta - {\widetilde{M}}_{T, {{\textbf {A}}}}\Delta )\Vert _2^2&\leqslant C \, h^2 \, \Vert \Psi \Vert _{H_{\text {mag}}^1(Q_h)}^2 \, \Vert D^2A_h\Vert _\infty ^2 \, \bigl ( 1 + \Vert A_h\Vert _\infty ^2 + \Vert DA_h\Vert _\infty ^2\bigr ) \nonumber \\&\quad \times \bigl \Vert (F_T^4+ G_T^2) * ( 1 + |\cdot |^2 ) |V\alpha _*| \bigr \Vert _2^2 \nonumber \\&\leqslant C \; h^8 \; \Vert \Psi \Vert _{H_{\text {mag}}^1(Q_h)}^2 \; \Vert (1+|\cdot |^2) V \alpha _* \Vert _2^2 . \end{aligned}$$

The second inequality is a consequence of Young’s inequality and (4.95). To prove the first inequality in (4.102), we first use (4.96) and argue as in the proof of (4.69) to see that

$$\begin{aligned}&\Vert {\widetilde{\pi }} ({\widetilde{L}}_{T, {{\textbf {A}}}}\Delta - {\widetilde{M}}_{T, {{\textbf {A}}}}\Delta )\Vert _2^2 \leqslant C \, h^2 \, \Vert \Psi \Vert _{H_{\text {mag}}^1(Q_h)}^2 \, \int _{{\mathbb {R}}^3} \text {d}r \, \Big ( \; \Bigl | \iint _{{\mathbb {R}}^3 \times {\mathbb {R}}^3} \text {d}Z \text {d}s \; |V\alpha _*(s)| \\&\qquad \qquad \times \bigl |{\widetilde{\pi }} k_T(Z, r-s) \text {e}^{\text {i}\frac{{{\textbf {B}}}}{4} \cdot (r\wedge s)}\bigr | \sup _{X\in {\mathbb {R}}^3} \bigl | \text {e}^{\text {i}{\widetilde{\Phi }}_{A_h}(X, Z, r, s) - \text {i}\Phi _{2A_h}(X, X+ Z) + \text {i}\frac{r-s}{4} \cdot DA_h(X) (r + s)} -1\bigr | \\&\quad + |k_T(Z, r-s)| \, \sup _{X\in {\mathbb {R}}^3} \bigl | \nabla _r \text {e}^{\text {i}{\widetilde{\Phi }}_{A_h}(X, Z, r, s)} - \nabla _r \text {e}^{\text {i}\Phi _{2A_h}(X, X+ Z)} \text {e}^{-\text {i}\frac{r-s}{4} \cdot DA_h(X) (r + s)}\bigr | \Bigr |^2 \Big ). \end{aligned}$$

A brief computation shows that the operator ${\widetilde{\pi }}$ obeys the following intertwining relation with respect to $\text {e}^{\text {i}\frac{{{\textbf {B}}}}{4} \cdot (r\wedge s)}$:

4.103 $$\begin{aligned} {\widetilde{\pi }}_r \, \text {e}^ {\frac{\text {i}}{4} {{\textbf {B}}}\cdot (r \wedge s)} = \text {e}^{\frac{\text {i}}{4} {{\textbf {B}}}\cdot (r\wedge s)} \bigl ( -\text {i}\nabla _r + \frac{1}{4} {{\textbf {B}}}\wedge (r-s)\bigr ). \end{aligned}$$

The notation ${\widetilde{\pi }}_r$ in the above equation highlights on which of the two variables the operator ${\widetilde{\pi }}$ is acting. An application of this identity shows

4.104 $$\begin{aligned} |{\widetilde{\pi }} k_T(Z,r-s) \text {e}^{\text {i}\frac{{{\textbf {B}}}}{4} (r\wedge s)}|&\leqslant |\nabla _r k_T(Z, r-s)| + \frac{|{{\textbf {B}}}|}{4} \, |r-s| \, |k_T(Z,r-s)|. \end{aligned}$$

Hence, a computation similar to that leading to (4.99) shows that

4.105 $$\begin{aligned}&\int _{{\mathbb {R}}^3} \text {d}Z \; |{\widetilde{\pi }} k_T(Z, r-s) \text {e}^{\text {i}\frac{{{\textbf {B}}}}{4} \cdot (r\wedge s)}| \nonumber \\&\quad \qquad \quad \times \sup _{X\in {\mathbb {R}}^3} \bigl | \text {e}^{\text {i}{\widetilde{\Phi }}_{A}(X, Z, r, s) - \text {i}\Phi _{2A}(X, X+ Z) + \text {i}\frac{r-s}{4} \cdot DA(X) (r + s)} -1\bigr |&\nonumber \\&\quad \leqslant C \; \Vert D^2A\Vert _\infty \; \bigl [ (F_T^4 + G_T^3)(r-s) + (F_T^2 + G_T^1)(r-s)\; |s|^2\bigr ] \end{aligned}$$

with the function $F_T^a$ in (4.93) and $G_T^a$ in (4.94). Let us also note that we estimate the factor $|{{\textbf {B}}}|$ coming from the second term in (4.104) by 1.

We also have

4.106 $$\begin{aligned} \bigl | \nabla _r \text {e}^{\text {i}{\widetilde{\Phi }}_{A}(X, Z, r, s)}&- \nabla _r \text {e}^{\text {i}\Phi _{2A}(X, X+ Z) - \text {i}\frac{r-s}{4} \cdot DA(X) (r + s)}\bigr | \nonumber \\&\leqslant \bigl | \text {e}^{\text {i}{\widetilde{\Phi }}_{A} (X, Z, r, s) - \text {i}\Phi _{2A}(X, X + Z)+ \text {i}\frac{r-s}{4} \cdot DA(X) (r + s)} - 1\bigr | |\nabla _r {\widetilde{\Phi }}_{A}(X, Z, r, s)| \nonumber \\&\quad + \bigl | \nabla _r {\widetilde{\Phi }}_{A}(X, Z,r, s) + \nabla _r \frac{r-s}{4} \cdot DA(X) (r+s)\bigr |. \end{aligned}$$

We use

4.107 $$\begin{aligned} |\nabla _r {\widetilde{\Phi }}_{A}(X, Z, r, s)|&\leqslant C \; \bigl ( \Vert A\Vert _\infty + \Vert DA\Vert _\infty \bigr ) \, \bigl ( \bigl | Z + \frac{r-s}{2} \bigr | + \bigl | Z - \frac{r-s}{2}\bigr | \bigr ) \end{aligned}$$

and Lemma 4.12 to see that the right side of (4.106) is bounded by

$$\begin{aligned} C \, \Vert D^2A\Vert _\infty&\bigl ( 1+ \Vert A\Vert _\infty + \Vert D A\Vert _\infty \bigr ) \; \bigl [ |s|^2 + |r-s|^2 + |Z|^2 \\&+ \bigl ( |s| + |r-s|\bigr ) \bigl ( |Z|+ |r-s|\bigr ) \bigl ( \bigl | Z+\frac{r-s}{2} \bigr | + \bigl | Z-\frac{r-s}{2} \bigr | \bigr ) \bigr ]. \end{aligned}$$

In combination with (4.74) these considerations imply

$$\begin{aligned} \int _{{\mathbb {R}}^3} \text {d}Z \; |k_T(Z,r-s)| \, \sup _{X\in {\mathbb {R}}^3}&\bigl | \nabla _r \text {e}^{\text {i}{\widetilde{\Phi }}_{A}(X, Z, r, s)} - \nabla _r \text {e}^{\text {i}\Phi _{2A}(X, X+ Z)} \text {e}^{-\text {i}\frac{r-s}{4} \cdot DA(X) (r + s)}\bigr | \\&\leqslant C\, \Vert D^2A\Vert _\infty \bigl ( 1 + \Vert A\Vert _\infty + \Vert DA\Vert _\infty \bigr ) \\&\quad \times \bigl [ F_T^3 (r-s) + F_T^2 (r-s) \, |s| + F_T^0 (r-s) \, |s|^2\bigr ]. \end{aligned}$$

When we combine this with (4.105), we obtain (4.102). $\square$

**4.5.3.3 The operator **$M_{T,{{\textbf {A}}}}$. We define the operator $M_{T,{{\textbf {A}}}}$ by

4.108 $$\begin{aligned} M_{T,{{\textbf {A}}}} \alpha (X,r) {:}{=}\iint _{{\mathbb {R}}^3 \times {\mathbb {R}}^3} \text {d}Z \text {d}s \; k_T(Z, r-s) \;(\cos (Z\cdot \Pi _{{{\textbf {A}}}_h})\alpha )(X,s), \end{aligned}$$

where $k_T$ is defined below (4.76). In our calculation, we may replace ${\widetilde{M}}_{T, {{\textbf {A}}}}$ by $M_{T,{{\textbf {A}}}}$ due to the following error bound.

##### Proposition 4.15

Assume that ${{\textbf {A}}}= {{\textbf {A}}}_{e_3} + A$ with a $A \in W^{2,\infty }({\mathbb {R}}^3,{\mathbb {R}}^3)$ periodic, let $|\cdot |^k V\alpha _*\in L^2({\mathbb {R}}^3)$ for $k \in \{ 0,1,2 \}$, $\Psi \in H_{\text {mag}}^1(Q_h)$, and denote $\Delta \equiv \Delta _\Psi$ as in (3.1). For any $T_0 > 0$ there is $h_0>0$ such that for any $0 < h \leqslant h_0$ and any $T\geqslant T_0$ we have

4.109 $$\begin{aligned} \Vert {\widetilde{M}}_{T,{{\textbf {A}}}}\Delta - M_{T,{{\textbf {A}}}}\Delta \Vert _{H^1(Q_h \times {\mathbb {R}}_{{\text {s}}}^3)}^2&\leqslant C\;h^6 \; \bigl ( \max _{k\in \{0,1,2\}} \Vert \, |\cdot |^k V\alpha _*\Vert _2^2\bigr ) \;\Vert \Psi \Vert _{H_{\text {mag}}^1(Q_h)}^2. \end{aligned}$$

Furthermore,

4.110 $$\begin{aligned} |\langle \Delta , {\widetilde{M}}_{T,{{\textbf {A}}}}\Delta - M_{T,{{\textbf {A}}}}\Delta \rangle |&\leqslant C \;h^6 \; \bigl ( \Vert V\alpha _*\Vert _2^2 + \Vert \, |\cdot |^2 V\alpha _*\Vert _2^2 \bigr ) \;\Vert \Psi \Vert _{H_{\text {mag}}^1(Q_h)}^2. \end{aligned}$$

##### Remark 4.16

The two bounds in (4.109) and (4.110) are needed for the proof of Proposition 3.2 and Theorem 3.6, respectively. We highlight that the bound in (4.109) is not strong enough to be useful in the proof of Theorem 3.6. More precisely, if we apply Cauchy–Schwarz and use Lemma 4.1 as well as (4.109) to estimate $\Vert \Delta \Vert _2$, we obtain a bound that is only of the order $h^4$. This is not good enough because $h^4$ is the order of the Ginzburg–Landau energy. To obtain (4.110), we exploit the fact that $V\alpha _*$ is real-valued, which allows us to replace $\exp (-\text {i}\frac{r-s}{4} \cdot D{{\textbf {A}}}_h(X)(r+s))$ in the definition of $\widetilde{M}_{T, {{\textbf {A}}}}$ by $\cos (\frac{r-s}{4} \cdot D{{\textbf {A}}}_h(X)(r+s))$ and to win an additional factor $h^2$.

##### Proof of Proposition 4.15

The proof is similar to that of Proposition 4.9. We begin by proving (4.109) and claim that

4.111 $$\begin{aligned} \Vert {\widetilde{M}}_{T, {{\textbf {A}}}}\Delta - M_{T, {{\textbf {A}}}}\Delta \Vert _2^2&\leqslant C \, \Vert \Psi \Vert _2^2 \, \Vert D{{\textbf {A}}}_h\Vert _\infty ^2 \, \Vert F_T^2 * |V\alpha _*| + F_T^1 * |\cdot |\, |V\alpha _*|\, \Vert _2^2 \end{aligned}$$

with the function $F_T^a$ in (4.93). If this holds, the desired bound for this term follows from Young’s inequality, (2.7), and the $L^1$-norm estimate on $F_T^a$ in (4.95). To prove (4.111), we argue as in (4.60)–(4.62) and obtain

4.112 $$\begin{aligned}&\Vert {\widetilde{M}}_{T, {{\textbf {A}}}}\Delta - M_{T, {{\textbf {A}}}}\Delta \Vert _2^2 \leqslant 4 \, \Vert \Psi \Vert _2^2 \nonumber \\&\quad \times \int _{{\mathbb {R}}^3} \text {d}r\, \Bigl | \iint _{{\mathbb {R}}^3 \times {\mathbb {R}}^3}\text {d}Z \text {d}s \; |k_T (Z, r-s)| \, \sup _{X\in {\mathbb {R}}^3} \bigl | \text {e}^{-\text {i}\frac{r-s}{4} \cdot D{{\textbf {A}}}_h(X) (r+s)} - 1\bigr | \;|V\alpha _*(s)|\Bigr |^2. \end{aligned}$$

When we combine the bound

4.113 $$\begin{aligned} \bigl | (r-s) \cdot D{{\textbf {A}}}(X) (r+s)\bigr |&\leqslant \Vert D{{\textbf {A}}}\Vert _\infty \, |r-s| \, \bigl ( 2 |s| + |r-s|\bigr ) \end{aligned}$$

$|r-s|$ in (4.74), we obtain

4.114 $$\begin{aligned} \int _{{\mathbb {R}}^3} \text {d}Z \; |k_T (Z, r-s)| \, \sup _{X\in {\mathbb {R}}^3}&\bigl | \text {e}^{-\text {i}\frac{r-s}{4} \cdot D{{\textbf {A}}}(X) (r+s)} - 1\bigr | \nonumber \\&\leqslant C\, \Vert D{{\textbf {A}}}\Vert _\infty \bigl [ F_T^2(r-s) + F_T^1 (r - s)\;|s|\bigr ]. \end{aligned}$$

In combination with (4.112), this proves (4.111).

We also claim that the term involving $\Pi$ is bounded by

4.115 $$\begin{aligned}&\Vert \Pi ({\widetilde{M}}_{T, {{\textbf {A}}}}\Delta - M_{T, {{\textbf {A}}}}\Delta )\Vert _2^2 \leqslant C\, h^2 \; \Vert \Psi \Vert _{H_{\text {mag}}^1(Q_h)}^2 \nonumber \\&\quad \times \bigl [ ( |{{\textbf {B}}}|^2 + \Vert D^2A_h\Vert _\infty ^2 )(1+ |{{\textbf {B}}}|^2 + \Vert D^2A_h\Vert _\infty ^2 ) \, \Vert F_T^3 *|V\alpha _*| + F_T^2 * |\cdot | \, |V\alpha _*| \, \Vert _2^2 \nonumber \\&\qquad + \Vert D^2{{\textbf {A}}}_h\Vert _\infty ^2\, \Vert F_T^2 * |V\alpha _*| + F_T^1 * |\cdot |\, |V\alpha _*|\, \Vert _2^2 \bigr ]. \end{aligned}$$

If this is correct, an application Young’s inequality and (4.95) shows the desired bound for this term. To prove (4.115), we first show that

4.116 $$\begin{aligned} \Vert \Pi \cos (Z\cdot \Pi _{{{\textbf {A}}}_h}) \Psi \Vert _2 \leqslant C \, h^2 \, (1 + |Z|) \, \Vert \Psi \Vert _{H_{\text {mag}}^1(Q_h)} \end{aligned}$$

holds. From Lemma 4.13 we know that

$$\begin{aligned} \text {e}^{\pm \text {i}Z \cdot \Pi _{{{\textbf {A}}}_h}} = \text {e}^{\pm \text {i}Z \cdot \Pi } \text {e}^{-\text {i}\Phi _{2A_h}(X, X {\mp } Z)} \end{aligned}$$

holds. An application of the intertwining relation in (4.67) for $\Pi _X$ and $\text {e}^{\text {i}Z\cdot \Pi _X}$ therefore shows

$$\begin{aligned}{}[\Pi , \text {e}^{\pm \text {i}Z\cdot \Pi _{{{\textbf {A}}}_h}}] = \text {e}^{\pm \text {i}Z\cdot \Pi _{{{\textbf {A}}}_h}} \bigl [ {\mp } 2\, {{\textbf {B}}}\wedge Z - \nabla _X \Phi _{2A_h}(X, X {\mp } Z)\bigr ]. \end{aligned}$$

We highlight that $\Pi$ and $\Pi _{{{\textbf {A}}}_h}$ in the two equations above act on the coordinate *X*. Using Lemma 4.5, we check that

$$\begin{aligned} \nabla _X \Phi _{2A}(X, X {\mp } Z) = 2A(X{\mp } Z) - 2A(X) + 2{\widetilde{A}}(X, X{\mp } Z) - 2{\widetilde{A}} (X{\mp } Z, X) \end{aligned}$$

holds. Accordingly, $|\nabla _X\Phi _{2A}(X, X {\mp } Z)|\leqslant C\, \Vert DA\Vert _\infty |Z|$, which implies

4.117 $$\begin{aligned} \Vert [\Pi , \cos (Z\cdot \Pi _{{{\textbf {A}}}_h})]\Psi \Vert _2 \leqslant C \, ( | {{\textbf {B}}}| + \Vert DA_h\Vert _\infty ) \, |Z| \, \Vert \Psi \Vert _2 \end{aligned}$$

as well as (4.116). Finally, a computation similar to the one leading to (4.69), Lemma 4.12, (4.114), (4.116), and the above considerations prove (4.115). It remains to consider the term proportional to ${\widetilde{\pi }}$.

With an argument that is similar to the one leading to (4.72), we see that

$$\begin{aligned}&\Vert {\widetilde{\pi }} ({\widetilde{M}}_{T, {{\textbf {A}}}}\Delta - M_{T, {{\textbf {A}}}}\Delta )\Vert _2^2 \leqslant 4\, \Vert \Psi \Vert _2^2 \\&\quad \times \int _{{\mathbb {R}}^3} \text {d}r \, \Bigl | \iint _{{\mathbb {R}}^3 \times {\mathbb {R}}^3}\text {d}Z \text {d}s \; \bigl | {\widetilde{\pi }} k_T (Z, r-s) \bigl [ \text {e}^{-\text {i}\frac{r-s}{4} \cdot D{{\textbf {A}}}_h(X) \cdot (r + s)} - 1\bigr ] \bigr | \; |V\alpha _*(s)|\Bigr |^2. \end{aligned}$$

We also note that

$$\begin{aligned} \bigl |\nabla _r (r - s) D{{\textbf {A}}}(X) (r+s) \bigr | \leqslant C \, \Vert D{{\textbf {A}}}\Vert _\infty \bigl ( |s| + |r-s|\bigr ). \end{aligned}$$

In combination with (4.113) and $|{{\textbf {A}}}_{{{\textbf {B}}}}(r)| \leqslant | {{\textbf {B}}}| \ (|r-s| + |s|)$, this implies

$$\begin{aligned} \int _{{\mathbb {R}}^3} \text {d}Z\; \bigl | {\widetilde{\pi }} k_T (Z, r-s)&\bigl [ \text {e}^{-\text {i}\frac{r-s}{4} \cdot D{{\textbf {A}}}(X) \cdot (r+s)} - 1\bigr ] \bigr | \\&\leqslant C\, ( |{{\textbf {B}}}| + \Vert DA\Vert _\infty ) \; \bigl ( (F_T^2 + G_T^2)(r-s) + (F_T^0 + G_T^1)(r-s) \; |s| \bigr ) \end{aligned}$$

with the function $F_T^a$ in (4.93) and $G_T^a$ in (4.94). When we apply Young’s inequality and (4.95), we obtain (4.109). It remains to prove (4.110).

To that end, we need to consider4.118The left side of this equation is real-valued. It therefore equals 1/2 times the right side plus 1/2 times the complex conjugate of the right side. When we use that $V \alpha _*$ is real-valued, that the Matsubara frequencies in (3.10) satisfy $-\omega _n = \omega _{-(n+1)}$, and the transformation $n \mapsto -n-1$ in the sum in the definition of $k_T(Z,r-s)$, we see that the complex conjugate of the right side equals the same expression with $\exp (-\text {i}\frac{r-s}{4} \cdot D{{\textbf {A}}}_h(X) (r+s))$ replaced by its complex conjugate. Using this and the identity $\cos (x) -1 =- 2\sin ^2(\frac{x}{2})$ we find4.119Furthermore, (4.113) implies

$$\begin{aligned} \sin ^2\bigl (\frac{1}{8} (r-s) \cdot D{{\textbf {A}}}_h(X)(r+s) \bigr ) \leqslant C \, \Vert D{{\textbf {A}}}_h\Vert _\infty ^2\, |r-s|^2 \, \bigl ( |s|^2+ |r-s|^2\bigr ). \end{aligned}$$

We use this bound, (4.119), and $\Vert \cos (Z\cdot \Pi _{{{\textbf {A}}}_h}) \Vert _{\infty } \leqslant 1$ to see that

$$\begin{aligned} |\langle \Delta , {\widetilde{M}}_{T, {{\textbf {A}}}}\Delta - M_{T, {{\textbf {A}}}}\Delta \rangle |&\leqslant \Vert D{{\textbf {A}}}_h\Vert _\infty ^2 \; \Vert \Psi \Vert _2^2 \; \bigl \Vert |V\alpha _*| \; \bigl ( F_T^4 * |V\alpha _*| + F_T^2 * |\cdot |^2 |V\alpha _*| \bigr ) \bigr \Vert _1. \end{aligned}$$

The desired bound in (4.110) follows when we apply Young’s inequality and use (2.7) as well as the $L^1$-norm estimate for $F_T^a$ in (4.95). This completes the proof of Proposition 4.15. $\square$

#### Analysis of $M_{T, {{\textbf {A}}}}$ and calculation of two quadratic terms in the Ginzburg–Landau functional

We decompose as $M_{T, {{\textbf {A}}}} = M_T^{(1)} + M_{T, {{\textbf {A}}}}^{(2)} + M_{T, {{\textbf {A}}}}^{(3)}$, where

4.120 $$\begin{aligned} M_T^{(1)} \alpha (X,r)&{:}{=}\iint _{{\mathbb {R}}^3\times {\mathbb {R}}^3} \text {d}Z \text {d}s \; k_T(Z, r-s) \; \alpha (X,s), \end{aligned}$$

4.121 $$\begin{aligned} M_{T, {{\textbf {A}}}}^{(2)} \alpha (X, r)&{:}{=}-\frac{1}{2} \iint _{{\mathbb {R}}^3\times {\mathbb {R}}^3} \text {d}Z \text {d}s\; k_T(Z, r-s) \; (Z\cdot \Pi _{{{\textbf {A}}}_h})^2 \; \alpha (X, s), \end{aligned}$$

4.122 $$\begin{aligned} M_{T, {{\textbf {A}}}}^{(3)} \alpha (X,r)&{:}{=}\iint _{{\mathbb {R}}^3\times {\mathbb {R}}^3} \text {d}Z \text {d}s\; k_T(Z, r-s) \; {\mathcal {R}}(Z\cdot \Pi _{{{\textbf {A}}}_h}) \; \alpha (X, s), \end{aligned}$$

and $${\mathcal {R}}(x) = \cos (x) - 1 + \frac{1}{2} x^2$$.

**4.5.4.1 The operator **$M_T^{(1)}$. From the quadratic form $\langle \Delta , M_T^{(1)} \Delta \rangle$ we extract the quadratic term without external fields or a gradient in the Ginzburg–Landau functional in (1.18). We also obtain a term that cancels the last term on the left side of (3.6). The relevant computation can be found in [15, Proposition 4.11]. For the sake of completeness, we state the result also here. We recall that $\Delta \equiv \Delta _\Psi = -2 V\alpha _* \Psi$ as in (3.1).

##### Proposition 4.17

Let $V\alpha _*\in L^2({\mathbb {R}}^3)$, $\Psi \in L_{\text {mag}}^2(Q_h)$, and $\Delta \equiv \Delta _\Psi$ as in (3.1). Then the following statements hold.

- We have $$M_{{T_{\text {c}}}}^{(1)} \Delta (X, r) = -2\, \alpha _* (r) \Psi (X)$$.
- For any $T_0>0$ there is a constant $c>0$ such that for $T_0 \leqslant T \leqslant {T_{\text {c}}}$ we have
  $$\begin{aligned} \langle \Delta , M_T^{(1)} \Delta - M_{{T_{\text {c}}}}^{(1)} \Delta \rangle \geqslant c \, \frac{{T_{\text {c}}}- T}{{T_{\text {c}}}} \; \Vert \Psi \Vert _2^2. \end{aligned}$$
- Given $D\in {\mathbb {R}}$ there is $h_0>0$ such that for $0< h\leqslant h_0$, and $T = {T_{\text {c}}}(1 - Dh^2)$ we have
  $$\begin{aligned} \langle \Delta , M_T^{(1)} \Delta - M_{{T_{\text {c}}}}^{(1)} \Delta \rangle = 4\; Dh^2 \; \Lambda _2 \; \Vert \Psi \Vert _2^2 + R(\Delta ) \end{aligned}$$
  with the coefficient $\Lambda _2$ in (3.22), and
  $$\begin{aligned} |R(\Delta )|&\leqslant C \; h^6 \; \Vert V\alpha _*\Vert _2^2\; \Vert \Psi \Vert _{H_{\text {mag}}^1(Q_h)}^2. \end{aligned}$$
- Assume additionally that $| \cdot | V\alpha _*\in L^2({\mathbb {R}}^3)$. Then, there is $h_0>0$ such that for any $0< h \leqslant h_0$, any $\Psi \in H_{\text {mag}}^1(Q_h)$, and any $T \geqslant T_0 > 0$ we have
  $$\begin{aligned} \Vert M_T^{(1)}\Delta - M_{{T_{\text {c}}}}^{(1)}\Delta \Vert _{{H^1(Q_h \times {\mathbb {R}}_{{\text {s}}}^3)}}^2&\leqslant C \, h^2 \, | T - {T_{\text {c}}}|^2 \, \bigl ( \Vert V\alpha _*\Vert _2^2 + \Vert \, |\cdot | V\alpha _*\Vert _2^2\bigr ) \Vert \Psi \Vert _{H_{\text {mag}}^1(Q_h)}^2. \end{aligned}$$

##### Remark 4.18

Parts (a) and (c) of the above proposition are needed for the proof of Theorem 3.6, part (b) is needed for the proof of Proposition 3.7, and part (d) for the proof of Proposition 3.2.

**4.5.4.2 The operator **$M_{T,{{\textbf {A}}}}^{(2)}$. The kinetic term in the Ginzburg–Landau functional in (1.18) is contained in $\langle \Delta , M_{T,{{\textbf {A}}}}^{(2)} \Delta \rangle$ with $M_{T,{{\textbf {A}}}}^{(2)}$ defined in (4.121). The following proposition allows us to extract this term.

##### Proposition 4.19

Let $V\alpha _* \in L^2({\mathbb {R}}^3)$ be a radial function, let $A\in W^{1,\infty }({\mathbb {R}}^3,{\mathbb {R}}^3)$ be periodic, assume $\Psi \in H_{\text {mag}}^1(Q_h)$, and denote $\Delta \equiv \Delta _\Psi$ as in (3.1). We then have

4.123 $$\begin{aligned} \langle \Delta , M_{{T_{\text {c}}},{{\textbf {A}}}}^{(2)} \Delta \rangle = - 4\; \Lambda _0 \; \Vert \Pi _{{{\textbf {A}}}_h} \Psi \Vert _2^2 \end{aligned}$$

with $\Lambda _0$ in (3.20). Moreover, for any $T \geqslant T_0 > 0$ we have

4.124 $$\begin{aligned} |\langle \Delta , M_{T,{{\textbf {A}}}}^{(2)} \Delta - M_{{T_{\text {c}}},{{\textbf {A}}}}^{(2)}\Delta \rangle | \leqslant C\; h^4 \; |T - {T_{\text {c}}}| \; \Vert V\alpha _*\Vert _2^2 \; \Vert \Psi \Vert _{H_{\text {mag}}^1(Q_h)}^2. \end{aligned}$$

##### Proof

The proof is analogous to the proof of [15, Proposition 4.13] with the obvious replacements, and is therefore omitted. $\square$

**4.5.4.3 The operator **$M_{T,{{\textbf {A}}}}^{(3)}$. The term $\langle \Delta , M_{T,{{\textbf {A}}}}^{(3)} \Delta \rangle$ with $M_{T,{{\textbf {A}}}}^{(3)}$ in (4.122) does not contribute to the Ginzburg–Landau energy. To obtain a bound for it, we need, as in [15], the $H_{\text {mag}}^2(Q_h)$-norm of $\Psi$.

##### Proposition 4.20

Let $V\alpha _* \in L^2({\mathbb {R}}^3)$, let $A\in W^{1,\infty }({\mathbb {R}}^3,{\mathbb {R}}^3)$ be periodic, assume that $\Psi \in H_{\text {mag}}^1(Q_h)$, and denote $\Delta \equiv \Delta _\Psi$ as in (3.1). For any $T_0>0$ there is $h_0>0$ such that for any $T\geqslant T_0$ and any $0 < h \leqslant h_0$ we have

$$\begin{aligned} |\langle \Delta , M_{T,{{\textbf {A}}}}^{(3)} \Delta \rangle |&\leqslant C \; h^6 \; \Vert V\alpha _*\Vert _2^2 \; \Vert \Psi \Vert _{H_{\text {mag}}^2(Q_h)}^2. \end{aligned}$$

Before we give the proof of Proposition 4.20, we state and prove the following lemma.

##### Lemma 4.21

Assume that ${{\textbf {A}}}= {{\textbf {A}}}_{e_3} + A$ with a periodic function $A\in W^{2,\infty }({\mathbb {R}}^3,{\mathbb {R}}^3)$.

- For $\varepsilon >0$ we have
  4.125 $$\begin{aligned} |Z\cdot \Pi _{{\textbf {A}}}|^4&\leqslant C\; |Z|^4 \; \bigl [ \Pi _{{\textbf {A}}}^4 + \varepsilon \, \Pi _{{\textbf {A}}}^2 + \, |{{\,\textrm{curl}\,}}{{\textbf {A}}}|^2 + \varepsilon ^{-1} \, \bigl ( |{{\,\textrm{curl}\,}}({{\,\textrm{curl}\,}}{{\textbf {A}}})|^2 + |\nabla ({{\,\textrm{curl}\,}}{{\textbf {A}}})|^2 \bigr ) \bigr ]. \end{aligned}$$
  The gradient in the last term is understood to act on each component of ${{\,\textrm{curl}\,}}A$ separately. The result is a vector field with nine components.
- Assume that $\Psi \in H_{\text {mag}}^2(Q_h)$. There is a constant $h_0>0$ such that for any $0 < h \leqslant h_0$, we have
  $$\begin{aligned} \langle \Psi , \, |Z\cdot \Pi _{{{\textbf {A}}}_h}|^4 \, \Psi \rangle&\leqslant C\; h^6 \; |Z|^4 \; \Vert \Psi \Vert _{H_{\text {mag}}^2(Q_h)}^2. \end{aligned}$$

##### Proof

We first give the proof of part (a) and start by noting that

4.126 $$\begin{aligned} \bigl [\Pi _{{\textbf {A}}}^{(i)}, \Pi _{{\textbf {A}}}^{(j)}\bigr ] = -\text {i}\sum _{k=1}^3 \varepsilon _{ijk} \; ({{\,\textrm{curl}\,}}{{\textbf {A}}})_k \end{aligned}$$

with the Levi–Civita symbol $\varepsilon _{ijk}$, which is defined as 1 if (*i*, *j*, *k*) is a cyclic permutation of $\{1, 2, 3\}$, as $-1$ if it is an anticyclic permutation, and zero if at least two indices coincide. We claim that

4.127 $$\begin{aligned} \Pi _{{\textbf {A}}}\; \Pi _{{\textbf {A}}}^2\; \Pi _{{\textbf {A}}}= \Pi _{{\textbf {A}}}^4 + 2 \, |{{\,\textrm{curl}\,}}{{\textbf {A}}}|^2 - {{\,\textrm{curl}\,}}({{\,\textrm{curl}\,}}{{\textbf {A}}}) \cdot \Pi _{{\textbf {A}}}. \end{aligned}$$

If this holds, then we can use the fact that all terms in the above equation except for the last are self-adjoint to see that

4.128 $$\begin{aligned} \bigl [ {{\,\textrm{curl}\,}}({{\,\textrm{curl}\,}}{{\textbf {A}}}) , \Pi _{{\textbf {A}}}\bigr ] = 0. \end{aligned}$$

To prove (4.127), we first note that

4.129 $$\begin{aligned} \Pi _{{\textbf {A}}}\; \Pi _{{\textbf {A}}}^2&= \Pi _{{\textbf {A}}}^2 \; \Pi _{{\textbf {A}}}+ 2 \sum _{i=1}^3 [\Pi _{{\textbf {A}}}, \Pi _{{\textbf {A}}}^{(i)}] \, \Pi _{{\textbf {A}}}^{(i)} + \sum _{i=1}^3 \bigl [\Pi _{{\textbf {A}}}^{(i)} , [\Pi _{{\textbf {A}}}, \Pi _{{\textbf {A}}}^{(i)}] \bigr ]. \end{aligned}$$

Moreover, an application of (4.126) shows that

4.130 $$\begin{aligned} \sum _{i=1}^3 [\Pi _{{\textbf {A}}}, \Pi _{{\textbf {A}}}^{(i)}] \, \Pi _{{\textbf {A}}}^{(i)} = \text {i}({{\,\textrm{curl}\,}}{{\textbf {A}}}) \wedge \Pi _{{\textbf {A}}} \end{aligned}$$

and

4.131 $$\begin{aligned} \sum _{i=1}^3 \bigl [\Pi _{{\textbf {A}}}^{(i)} , [\Pi _{{\textbf {A}}}, \Pi _{{\textbf {A}}}^{(i)}] \bigr ] = - {{\,\textrm{curl}\,}}({{\,\textrm{curl}\,}}{{\textbf {A}}}). \end{aligned}$$

We combine (4.129)-(4.131) and find

$$\begin{aligned} \Pi _{{\textbf {A}}}\; \Pi _{{\textbf {A}}}^2 \; \Pi _{{\textbf {A}}}= \Pi _{{\textbf {A}}}^4 + 2\text {i}\bigl ( ({{\,\textrm{curl}\,}}{{\textbf {A}}}) \wedge \Pi _{{\textbf {A}}}\bigr ) \cdot \Pi _{{\textbf {A}}}- {{\,\textrm{curl}\,}}({{\,\textrm{curl}\,}}{{\textbf {A}}}) \cdot \Pi _{{\textbf {A}}}\end{aligned}$$

Using additionally the identity $\text {i}( ({{\,\textrm{curl}\,}}{{\textbf {A}}}) \wedge \Pi _{{\textbf {A}}}) \cdot \Pi _{{\textbf {A}}}= |{{\,\textrm{curl}\,}}{{\textbf {A}}}|^2$, we conclude that (4.127) holds.

Our next goal is to prove the formula

4.132 $$\begin{aligned} (Z\cdot \Pi _{{\textbf {A}}}) \, \Pi _{{\textbf {A}}}^2\, (Z\cdot \Pi _{{\textbf {A}}})&= \Pi _{{\textbf {A}}}\, (Z\cdot \Pi _{{\textbf {A}}})^2 \, \Pi _{{\textbf {A}}}+ (Z \cdot {{\,\textrm{curl}\,}}({{\,\textrm{curl}\,}}{{\textbf {A}}})) \; (Z \cdot \Pi _{{\textbf {A}}}) \nonumber \\&\quad + (Z \wedge \Pi _{{\textbf {A}}}) \cdot \bigl ( (Z\cdot \nabla ) ({{\,\textrm{curl}\,}}{{\textbf {A}}})\bigr ), \end{aligned}$$

which implies

4.133 $$\begin{aligned} \bigl [ Z \cdot {{\,\textrm{curl}\,}}({{\,\textrm{curl}\,}}{{\textbf {A}}}), Z\cdot \Pi _{{\textbf {A}}}\bigr ] + \bigl [ Z \wedge \Pi _{{\textbf {A}}}, (Z\cdot \nabla ) ({{\,\textrm{curl}\,}}{{\textbf {A}}}) \bigr ] =0. \end{aligned}$$

We note that

4.134 $$\begin{aligned} (Z\cdot \Pi _{{\textbf {A}}}) \, \Pi _{{\textbf {A}}}^2\, (Z\cdot \Pi _{{\textbf {A}}}) = \sum _{i,j =1}^3 Z_iZ_j \; \Pi _{{\textbf {A}}}^{(i)} \, \Pi _{{\textbf {A}}}^2 \, \Pi _{{\textbf {A}}}^{(j)} \end{aligned}$$

and

$$\begin{aligned} \Pi _{{\textbf {A}}}^{(i)} \, \Pi _{{\textbf {A}}}^2 \, \Pi _{{\textbf {A}}}^{(j)} = \sum _{m = 1}^3 \bigl ( \Pi _{{\textbf {A}}}^{(m)} \, \Pi _{{\textbf {A}}}^{(i)} \, \Pi _{{\textbf {A}}}^{(j)} \, \Pi _{{\textbf {A}}}^{(m)} + \Pi _{{\textbf {A}}}^{(m)} \, \Pi _{{\textbf {A}}}^{(i)} \, [ \Pi _{{\textbf {A}}}^{(m)}, \Pi _{{\textbf {A}}}^{(j)}] + [\Pi _{{\textbf {A}}}^{(i)}, \Pi _{{\textbf {A}}}^{(m)}] \, \Pi _{{\textbf {A}}}^{(m)} \, \Pi _{{\textbf {A}}}^{(j)} \bigr ). \end{aligned}$$

The sum in (4.134) is left unchanged when we exchange the indices *i* and *j*. Motivated by this, we combine $\frac{1}{2}$ times the original term and $\frac{1}{2}$ times the term with *i* and *j* interchanged and find

4.135 $$\begin{aligned} \Pi _{{\textbf {A}}}^{(i)} \, \Pi _{{\textbf {A}}}^2 \, \Pi _{{\textbf {A}}}^{(j)} + \Pi _{{\textbf {A}}}^{(j)} \, \Pi _{{\textbf {A}}}^2 \, \Pi _{{\textbf {A}}}^{(i)}&= \sum _{m=1}^3 \bigl ( \Pi _{{\textbf {A}}}^{(m)} \, \Pi _{{\textbf {A}}}^{(i)} \, \Pi _{{\textbf {A}}}^{(j)} \, \Pi _{{\textbf {A}}}^{(m)} + \Pi _{{\textbf {A}}}^{(m)} \, \Pi _{{\textbf {A}}}^{(j)} \, \Pi _{{\textbf {A}}}^{(i)} \, \Pi _{{\textbf {A}}}^{(m)} \nonumber \\&\quad + \bigl [ [\Pi _{{\textbf {A}}}^{(i)}, \Pi _{{\textbf {A}}}^{(m)}] , \Pi _{{\textbf {A}}}^{(m)} \, \Pi _{{\textbf {A}}}^{(j)}\bigr ] + \bigl [ [\Pi _{{\textbf {A}}}^{(j)}, \Pi _{{\textbf {A}}}^{(m)}] , \Pi _{{\textbf {A}}}^{(m)} \, \Pi _{{\textbf {A}}}^{(i)}\bigr ] \bigr ). \end{aligned}$$

Using the commutator identity $[A, BC] = B\, [A, C] + [A, B] \, C$ we write the third term as

4.136 $$\begin{aligned} \bigl [ [\Pi _{{\textbf {A}}}^{(i)}, \Pi _{{\textbf {A}}}^{(m)}] , \Pi _{{\textbf {A}}}^{(m)} \, \Pi _{{\textbf {A}}}^{(j)}\bigr ]&= \Pi _{{\textbf {A}}}^{(m)} \bigl [ [\Pi _{{\textbf {A}}}^{(i)}, \Pi _{{\textbf {A}}}^{(m)}] , \Pi _{{\textbf {A}}}^{(j)}\bigr ] + \bigl [ [\Pi _{{\textbf {A}}}^{(i)}, \Pi _{{\textbf {A}}}^{(m)}] , \Pi _{{\textbf {A}}}^{(m)} \bigr ] \, \Pi _{{\textbf {A}}}^{(j)} \end{aligned}$$

and likewise for the term with *i* and *j* interchanged. We also use (4.131) to see that

$$\begin{aligned} \frac{1}{2} \sum _{i,j =1}^3 Z_iZ_j \Bigl ( \bigl [ [\Pi _{{\textbf {A}}}^{(j)} , \Pi _{{\textbf {A}}}] , \Pi _{{\textbf {A}}}\bigr ] \Pi _{{\textbf {A}}}^{(i)} + \bigl [ [\Pi _{{\textbf {A}}}^{(i)} , \Pi _{{\textbf {A}}}] , \Pi _{{\textbf {A}}}\bigr ] \Pi _{{\textbf {A}}}^{(j)} \Bigr ) = (Z \cdot {{\,\textrm{curl}\,}}({{\,\textrm{curl}\,}}{{\textbf {A}}}) )\; (Z\cdot \Pi _{{\textbf {A}}}) \end{aligned}$$

holds. Concerning the first term on the right side of (4.136), (4.126) can be used to show

$$\begin{aligned} \bigl [ [\Pi _{{\textbf {A}}}^{(i)}, \Pi _{{\textbf {A}}}^{(m)}] , \Pi _{{\textbf {A}}}^{(j)}\bigr ] = \sum _{k=1}^3 \varepsilon _{imk} \, \partial _j ({{\,\textrm{curl}\,}}{{\textbf {A}}})_k, \end{aligned}$$

which implies

4.137 $$\begin{aligned}&\frac{1}{2} \sum _{i,j=1}^3 Z_i Z_j \bigl ( \Pi _{{{\textbf {A}}}} \bigl [ [ \Pi _{{{\textbf {A}}}}^{(i)}, \Pi _{{{\textbf {A}}}} ], \Pi _{{{\textbf {A}}}}^{(j)} \bigr ] + \Pi _{{{\textbf {A}}}} \bigl [ [ \Pi _{{{\textbf {A}}}}^{(j)}, \Pi _{{{\textbf {A}}}} ], \Pi _{{{\textbf {A}}}}^{(i)} \bigr ] \bigr ) = (Z \wedge \Pi _{{\textbf {A}}}) \cdot \bigl ( (Z\cdot \nabla ) ({{\,\textrm{curl}\,}}{{\textbf {A}}})\bigr ). \end{aligned}$$

When we combine (4.134)-(4.137), this proves proves (4.132). We are now prepared to give the proof of (4.125).

We start by noting that $|A + B + C|^2 \leqslant 3(|A|^2 + |B|^2 + |C|^2)$ holds for three linear operators *A*, *B*, *C*, which implies

4.138 $$\begin{aligned} (Z\cdot \Pi _{{\textbf {A}}})^2&\leqslant 3 \; \bigl ( Z_1^2 \; (\Pi _{{\textbf {A}}}^{(1)})^2 + Z_2^2 \; (\Pi _{{\textbf {A}}}^{(2)})^2 + Z_3^2 \; (\Pi _{{\textbf {A}}}^{(3)})^2\bigr ) \leqslant 3 \; |Z|^2 \; \Pi _{{\textbf {A}}}^2. \end{aligned}$$

We use (4.127), (4.132), and (4.138) to see that

$$\begin{aligned} (Z\cdot \Pi _{{\textbf {A}}}) \, \Pi _{{\textbf {A}}}^2\, (Z\cdot \Pi _{{\textbf {A}}})&\leqslant 3\, |Z|^2 \, \bigl (\Pi _{{\textbf {A}}}^4+ 2\, |{{\,\textrm{curl}\,}}{{\textbf {A}}}|^2 - {{\,\textrm{curl}\,}}({{\,\textrm{curl}\,}}{{\textbf {A}}}) \cdot \Pi _{{\textbf {A}}}\bigr ) \\&\quad + (Z \cdot {{\,\textrm{curl}\,}}({{\,\textrm{curl}\,}}{{\textbf {A}}})) \, (Z\cdot \Pi _{{\textbf {A}}}) + (Z \wedge \Pi _{{\textbf {A}}}) \cdot \bigl ( (Z\cdot \nabla ) ({{\,\textrm{curl}\,}}{{\textbf {A}}})\bigr ). \end{aligned}$$

Next, we write $|Z\cdot \Pi _{{\textbf {A}}}|^4 = (Z\cdot \Pi _{{\textbf {A}}}) (Z\cdot \Pi _{{\textbf {A}}})^2 (Z\cdot \Pi _{{\textbf {A}}})$, apply (4.138) to the term in the middle, and find

$$\begin{aligned} |Z\cdot \Pi _{{\textbf {A}}}|^4&\leqslant 9 \, |Z|^4 \bigl ( \Pi _{{\textbf {A}}}^4 + 2\, |{{\,\textrm{curl}\,}}{{\textbf {A}}}|^2 - {{\,\textrm{curl}\,}}({{\,\textrm{curl}\,}}{{\textbf {A}}}) \cdot \Pi _{{\textbf {A}}}\bigr ) \\&\quad + 3 \, |Z|^2 \bigl [ (Z \cdot {{\,\textrm{curl}\,}}({{\,\textrm{curl}\,}}{{\textbf {A}}})) \, (Z\cdot \Pi _{{\textbf {A}}}) + (Z \wedge \Pi _{{\textbf {A}}}) \cdot \bigl ( (Z\cdot \nabla ) ({{\,\textrm{curl}\,}}{{\textbf {A}}})\bigr )\bigr ]. \end{aligned}$$

Moreover, $AB + BA \leqslant \varepsilon \, A^2 + \frac{1}{4 \varepsilon } \, B^2$ for $\varepsilon >0$ and self-adjoint operators *A* and *B*, together with (4.128), (4.133), $|Z\wedge \Pi _{{\textbf {A}}}|^2 \leqslant 3 |Z|^2 \Pi _{{\textbf {A}}}^2$, and (4.138) imply that the right side of the above equation is bounded by

$$\begin{aligned} C \, |Z|^4 \bigl [ \Pi _{{\textbf {A}}}^4 + \, |{{\,\textrm{curl}\,}}{{\textbf {A}}}|^2 + \varepsilon \, \Pi _{{\textbf {A}}}^2 + \varepsilon ^{-1} \bigl ( |{{\,\textrm{curl}\,}}({{\,\textrm{curl}\,}}{{\textbf {A}}})|^2 + |\nabla ({{\,\textrm{curl}\,}}{{\textbf {A}}})|^2 \bigr )\bigr ]. \end{aligned}$$

This proves part (a).

The proof of part (b) is a direct consequence of part (a) with the choice $\varepsilon = h^2$, and is therefore left to the reader. This proves Lemma 4.21. $\square$

##### Proof of Proposition 4.20

We use the definition of $M_{T, {{\textbf {A}}}}^{(3)}$ to write

4.139 $$\begin{aligned} \langle \Delta , M_{T, {{\textbf {A}}}}^{(3)} \Delta \rangle&= 4 \iiint _{{\mathbb {R}}^3\times {\mathbb {R}}^3\times {\mathbb {R}}^3} \text {d}r\text {d}s\text {d}Z \; V\alpha _*(r) V\alpha _*(s)\, k_T(Z, r-s) \; \langle \Psi , {\mathcal {R}}(Z\cdot \Pi _{{{\textbf {A}}}_h})\Psi \rangle . \end{aligned}$$

The function ${\mathcal {R}}(x) = \cos (x) - 1 + \frac{x^2}{2}$ satisfies the bound $0\leqslant {\mathcal {R}}(x) \leqslant \frac{1}{24} x^4$, and hence an application of Lemma 4.21 shows

4.140 $$\begin{aligned} \langle \Psi , {\mathcal {R}}(Z\cdot \Pi _{{{\textbf {A}}}_h}) \Psi \rangle&\leqslant C\; h^6 \; |Z|^4 \; \Vert \Psi \Vert _{H_{\text {mag}}^2(Q_h)}^2. \end{aligned}$$

When we apply the estimate $|Z|^4 \leqslant | Z + \frac{r}{2} |^4 + | Z - \frac{r}{2} |^4$, we see that

4.141 $$\begin{aligned} \int _{{\mathbb {R}}^3} \text {d}Z\; |Z|^4 \; |k_T(Z,r)|&\leqslant F_T^4(r) \end{aligned}$$

holds with $F_T^4$ defined in (4.93). But this also shows

$$\begin{aligned} |\langle \Delta , M_{T, {{\textbf {A}}}}^{(3)} \Delta \rangle | \leqslant C\; h^6 \; \Vert \Psi \Vert _{H_{\text {mag}}^2(Q_h)}^2 \; \Vert (V \alpha _*) F_T^4 *(V \alpha _*) \Vert _1. \end{aligned}$$

In combination with the $L^1({\mathbb {R}}^3)$-norm bound for $F_T^4$ in (4.95), this proves the claim. $\square$

#### A representation formula for the operator ${\mathcal {W}}_{T,{{\textbf {A}}}}$

In the next five subsections we study the operator ${\mathcal {W}}_{T, {{\textbf {A}}}}$ in (4.43). In particular, we extract the term in the GL functional that is proportional to *W* from $\langle \Delta , {\mathcal {W}}_{T, {{\textbf {A}}}} \Delta \rangle$. The operator ${\mathcal {W}}_{T, 0}$ has previously been studied in [19]. After the magnetic field has been removed, our analysis mostly follows ideas in this reference. Because of this and because several ideas of the previous sections appear again, we keep our presentation rather short and only mention the main ideas. As in the case of $L_{T, {{\textbf {A}}}}$, we start our analysis with a representation formula for ${\mathcal {W}}_{T, {{\textbf {A}}}}$ in terms of relative and center-of-mass coordinates.

##### Lemma 4.22

The operator ${\mathcal {W}}_{T, {{\textbf {A}}}} :{L^2(Q_h \times {\mathbb {R}}_{{\text {s}}}^3)}\rightarrow {L^2(Q_h \times {\mathbb {R}}_{{\text {s}}}^3)}$ in (4.43) acts as

4.142 $$\begin{aligned} {\mathcal {W}}_{T, {{\textbf {A}}}} \alpha (X, r)&= \iint _{{\mathbb {R}}^3 \times {\mathbb {R}}^3} \text {d}Z \text {d}s \; k_{T, {{\textbf {A}}}, W}(X, Z, r, s) \; (\text {e}^{\text {i}Z \cdot (-\text {i}\nabla _X)} \alpha )(X, s), \end{aligned}$$

where

4.143 $$\begin{aligned} k_{T, {{\textbf {A}}}, W} (X, Z, r, s)&{:}{=}\smash {\frac{2}{\beta }\sum _{n\in {\mathbb {Z}}} \int _{{\mathbb {R}}^3} \text {d}Y} \; W_h(X + Y) \bigl [ k_{T, {{\textbf {A}}}, +}^n(X, Y, Z, r, s) \; \text {e}^{\text {i}\Theta _{{{\textbf {A}}}_h}^+(Y, Z, r, s)} \nonumber \\&\quad + k_{T, {{\textbf {A}}}, -}^n(X, Y, Z, r, s) \; \text {e}^{\text {i}\Theta _{{{\textbf {A}}}_h}^-(Y, Z, r, s)} \bigr ], \end{aligned}$$

where

4.144 $$\begin{aligned} k_{T, {{\textbf {A}}}, \pm }^n(X, Y, Z, r, s)&{:}{=}g_h^{\pm \text {i}\omega _n} \biggl ( X \pm \frac{r}{2}, X + Y\biggr ) \; g_h^{\pm \text {i}\omega _n} \biggl ( X + Y, X + Z \pm \frac{s}{2}\biggr )\nonumber \\&\quad \times g_h^{{\mp } \text {i}\omega _n} \biggl ( X {\mp } \frac{r}{2}, X + Z {\mp } \frac{s}{2}\biggr ). \end{aligned}$$

The function $g_h^z$ is defined in (4.3) and

4.145 $$\begin{aligned} \Theta _{{\textbf {A}}}^\pm (Y, Z,r, s)&{:}{=}\Phi _{{\textbf {A}}}\biggl ( X \pm \frac{r}{2}, X + Y\biggr ) + \Phi _{{\textbf {A}}}\biggl ( X + Y, X + Z \pm \frac{s}{2}\biggr )\nonumber \\&\quad + \Phi _{{\textbf {A}}}\biggl ( X {\mp } \frac{r}{2}, X + Z {\mp } \frac{s}{2}\biggr ). \end{aligned}$$

##### Proof

The proof that ${\mathcal {W}}_{T, {{\textbf {A}}}}$ is a bounded linear map on ${L^2(Q_h \times {\mathbb {R}}_{{\text {s}}}^3)}$ goes along the same lines as that of Lemma 3.4. The proof of the representation formula is analogous to the proof of Lemma 4.7. We use (4.40) for $W=0$ to write

4.146 $$\begin{aligned} {\mathcal {W}}_{T, {{\textbf {A}}}} \alpha (x, y)&= \frac{2}{\beta }\sum _{n\in {\mathbb {Z}}} \iiint _{{\mathbb {R}}^9} \text {d}u \text {d}v \text {d}w \; \bigl [ G_h^{\text {i}\omega _n} (x, u) \, W_h(u) \, G_h^{\text {i}\omega _n} (u,v) \, \alpha (v, w) \, G_h^{-\text {i}\omega _n}(y, w) \nonumber \\&\quad + G_{{{\textbf {A}}}_h}^{\text {i}\omega _n} (x, v) \, \alpha (v, w) \, G_h^{-\text {i}\omega _n} (u,w) \, W_h(u) \, G_h^{-\text {i}\omega _n}(y, u)\bigr ]. \end{aligned}$$

When we define the coordinates $X = \frac{x + y}{2}$ and $r = x-y$, apply the change of variables

$$\begin{aligned} u&= X + Y,&v&= X + Z + \frac{s}{2},&w&= X + Z - \frac{s}{2}, \end{aligned}$$

and use (4.3), this yields (4.142). We highlight that, by a slight abuse of notation, we denoted the function $\alpha$ depending on the original coordinates in (4.146) and the function depending on relative and center-of-mass coordinates in (4.142) by the same symbol. $\square$

#### Approximation of the operator ${\mathcal {W}}_{T, {{\textbf {A}}}}$

The operator ${\mathcal {W}}_{T, {{\textbf {A}}}}$ will be analyzed in three steps. More precisely, we write

4.147 $$\begin{aligned} {\mathcal {W}}_{T, {{\textbf {A}}}} = \bigl ( {\mathcal {W}}_{T, {{\textbf {A}}}} - \widetilde{\mathcal {W}}_{T, {{\textbf {A}}}} \bigr ) + \bigl ( {\widetilde{{\mathcal {W}}}}_{T, {{\textbf {A}}}} - {\mathcal {W}}_T \bigr ) + {\mathcal {W}}_T, \end{aligned}$$

where ${\widetilde{{\mathcal {W}}}}_{T, {{\textbf {A}}}}$ and ${\mathcal {W}}_T$ are operators of increasing simplicity in their dependence on *W* and ${{\textbf {A}}}$. They are defined below in (4.148) and (4.151), respectively. The term in the Ginzburg–Landau functional that is proportional to *W* will be extracted from the expectation of the operator ${\mathcal {W}}_T$ with respect to $\Delta$. The expectation of the first two terms in (4.147) will be shown to be negligible.

**4.5.6.1 The operator **$${\widetilde{{\mathcal {W}}}}_{T, {{\textbf {A}}}}$$.

We define the operator

4.148 $$\begin{aligned} {\widetilde{{\mathcal {W}}}}_{T, A} \alpha (X, r)&{:}{=}W_h(X) \iint _{{\mathbb {R}}^3 \times {\mathbb {R}}^3} \text {d}Z \text {d}s \; k_{T, {{\textbf {A}}}, 0}(X, Z, r, s) \; (\text {e}^{\text {i}Z \cdot (-\text {i}\nabla _X)} \alpha )(X, s), \end{aligned}$$

where $k_{T, {{\textbf {A}}}, W}$ is defined in (4.143). The following proposition allows us to estimate the expectation of the first term in (4.147) with respect to $\Delta$.

##### Proposition 4.23

Let $| \cdot |^k V\alpha _* \in L^2({\mathbb {R}}^3)$ for $k \in \{ 0,1 \}$, let $A\in W^{3,\infty }({\mathbb {R}}^3,{\mathbb {R}}^3)$ and $W \in W^{1,\infty }({\mathbb {R}}^3,{\mathbb {R}})$ be periodic, assume $\Psi \in H_{\text {mag}}^1(Q_h)$, and denote $\Delta \equiv \Delta _\Psi$ as in (3.1). For any $T_0>0$ there is $h_0>0$ such that for any $T\geqslant T_0$ and any $0 < h \leqslant h_0$ we have

$$\begin{aligned} |\langle \Delta , {\mathcal {W}}_{T, {{\textbf {A}}}} \Delta - \widetilde{\mathcal {W}}_{T, {{\textbf {A}}}} \Delta \rangle | \leqslant C\, h^5\, \max _{k=0,1} \Vert \, |\cdot |^k \ V\alpha _*\Vert _2^2 \, \Vert \Psi \Vert _{H_{\text {mag}}^1(Q_h)}^2. \end{aligned}$$

##### Proof

We write

4.149 $$\begin{aligned}&|\langle \Delta , {\mathcal {W}}_{T, {{\textbf {A}}}}\Delta - {\widetilde{{\mathcal {W}}}}_{T, {{\textbf {A}}}} \Delta \rangle | \nonumber \\&\quad \leqslant 4 \, \Vert \Psi \Vert _2^2 \iiint _{{\mathbb {R}}^9} \text {d}Z \text {d}r \text {d}s \; \mathop {\mathrm {ess \, sup}}\limits _{X\in {\mathbb {R}}^3} |(k_{T, {{\textbf {A}}}, W} - k_{T, {{\textbf {A}}}, 0})(X, Z, r, s)| \; |V\alpha _*(r)| \, |V\alpha _*(s)| \end{aligned}$$

and note that

4.150 $$\begin{aligned}&|(k_{T, {{\textbf {A}}}, W} - k_{T, {{\textbf {A}}}, W})(X, Z, r, s)| \nonumber \\&\quad \leqslant \frac{2}{\beta }\sum _{n\in {\mathbb {Z}}} \int _{{\mathbb {R}}^3} \text {d}Y \; |W_h(X + Y) - W_h(X)| \; \bigl ( |k_{T, {{\textbf {A}}}, +}^n| + |k_{T, {{\textbf {A}}}, -}^n|\bigr )(X, Y, Z, r, s). \end{aligned}$$

When we use (4.150), $|W(X + Y) - W(X)|\leqslant \Vert \nabla W\Vert _\infty \, |Y|$, $|Y| \leqslant |Y \pm \frac{r}{2}| + |\frac{r}{2}|$, and Proposition 4.2, we see that the right side of (4.149) is bounded by a constant times

$$\begin{aligned} \Vert \Psi \Vert _2^2 \; \Vert \nabla W_h \Vert _{\infty } \; \Vert (1+|\cdot |) (V \alpha _*) {\widetilde{F}}_{T}^1 *| V \alpha _* | \ \Vert _1, \end{aligned}$$

where

$$\begin{aligned} {\widetilde{F}}_{T}^a {:}{=}\frac{2}{\beta }\sum _{n\in {\mathbb {Z}}} \sum _{b=0}^a \left[ \bigl ( |\cdot |^b \, \rho ^{ \text {i}\omega _n}\bigr ) * \rho ^{\text {i}\omega _n} * \rho ^{- \text {i}\omega _n} + \bigl ( |\cdot |^b \, \rho ^{- \text {i}\omega _n}\bigr ) * \rho ^{- \text {i}\omega _n} * \rho ^{ \text {i}\omega _n} \right] . \end{aligned}$$

With Proposition 4.2 and (4.56) we see that $\Vert {\widetilde{F}}_{T}^a\Vert _1 \leqslant C$. In combination with the bound $\Vert \nabla W_h\Vert _\infty \leqslant Ch^3$, this proves the claim. $\square$

**4.5.6.2 The operator **$${\mathcal {W}}_T$$.

We define the operator ${\mathcal {W}}_T$ by

4.151 $$\begin{aligned} {\mathcal {W}}_T \alpha (X, r)&{:}{=}W_h(X) \iint _{{\mathbb {R}}^3 \times {\mathbb {R}}^3} \text {d}Z \text {d}s \; k_T(Z, r-s) \; \alpha (X, s) \end{aligned}$$

where

4.152 $$\begin{aligned} k_T(Z, r)&{:}{=}\frac{2}{\beta }\sum _{n\in {\mathbb {Z}}} \bigl ( k_{T, +}^n (Z, r) + k_{T,-}^n(Z, r) \bigr ) \end{aligned}$$

and

4.153 $$\begin{aligned} k_{T, \pm }^n(Z, r)&{:}{=}(g_0^{\pm \text {i}\omega _n} * g_0^{\pm \text {i}\omega _n})\biggl ( Z {\mp } \frac{r}{2}\biggr )\; g_0^{{\mp } \text {i}\omega _n} \biggl ( Z \pm \frac{r}{2}\biggr ). \end{aligned}$$

##### Proposition 4.24

Let $| \cdot |^k V\alpha _* \in L^2({\mathbb {R}}^3)$ for $k \in \{ 0,1 \}$, let $A\in W^{3,\infty }({\mathbb {R}}^3,{\mathbb {R}}^3)$ and $W \in L^{\infty }({\mathbb {R}}^3,{\mathbb {R}})$ be periodic, assume $\Psi \in H_{\text {mag}}^1(Q_h)$, and denote $\Delta \equiv \Delta _\Psi$ as in (3.1). For any $T_0>0$ there is $h_0>0$ such that for any $T\geqslant T_0$ and any $0 < h \leqslant h_0$ we have

$$\begin{aligned} |\langle \Delta , {\widetilde{{\mathcal {W}}}}_{T, {{\textbf {A}}}} \Delta - {\mathcal {W}}_T \Delta \rangle | \leqslant C \, h^5 \, \max _{k=0,1} \Vert \, |\cdot |^k \ V\alpha _*\Vert _2^2 \; \Vert \Psi \Vert _{H_{\text {mag}}^1(Q_h)}^2. \end{aligned}$$

##### Sketch of proof

The proof goes along the same lines as that of Propositions 4.9, 4.11, and 4.15 with the notable simplification that we only need to prove bounds for the quadratic form. We therefore only mention the main steps that need to be carried out and leave the details to the reader. In the first step $g_h^z$ is replaced by $g_0^z$. In the second step the part of the magnetic phase coming from ${{\textbf {A}}}_{{\textbf {B}}}$ is split off. A careful analysis shows that

$$\begin{aligned} \Theta _{{{\textbf {A}}}_{{\textbf {B}}}}^\pm (X, Y, Z, r, s) = Z \cdot ({{\textbf {B}}}\wedge X) + \frac{{{\textbf {B}}}}{2} \cdot \theta _\pm (Y, Z, r, s), \end{aligned}$$

where

4.154 $$\begin{aligned} \theta _\pm (Y, Z, r, s)&{:}{=}\pm \frac{r}{2}\wedge \left( Y {\mp } \frac{r}{2}\right) + \left( Y {\mp } \frac{r}{2}\right) \wedge \left( Z - Y \pm \frac{s}{2}\right) \nonumber \\&\quad \pm \frac{r}{2}\wedge \left( Z - Y\pm \frac{s}{2}\right) {\mp } \frac{r}{2} \wedge \left( Z\pm \frac{r-s}{2}\right) . \end{aligned}$$

The phase $\exp (\text {i}Z \cdot ({{\textbf {B}}}\wedge X))$ and $\exp (\text {i}Z \cdot (-\text {i}\nabla _X))$ are combined and give $\exp (\text {i}Z \cdot \Pi )$, see (4.90). In the third step the magnetic phases coming from $\theta _\pm$ and from the periodic vector potential $A$, that is, from $\Theta _{A}$, are removed. Afterwards, the emergent symmetry of the integrand under the transformation $Z \rightarrow -Z$ is used to replace the operator $\exp (\text {i}Z \cdot \Pi )$ by $\cos (Z\cdot \Pi )$. In the final step, we apply the estimate $1 - \cos (Z\cdot \Pi )\leqslant C |Z|^2 \, \Pi ^2$. This ends our sketch of proof. $\square$

#### Analysis of ${\mathcal {W}}_T$ and calculation of the quadratic *W*-term

##### Proposition 4.25

Let $V\alpha _* \in L^2({\mathbb {R}}^3)$, let $W \in L^{\infty }({\mathbb {R}}^3,{\mathbb {R}})$ be periodic, assume that $\Psi \in H_{\text {mag}}^1(Q_h)$, and denote $\Delta \equiv \Delta _\Psi$ as in (3.1). There is $h_0>0$ such that for any $0< h \leqslant h_0$ we have

4.155 $$\begin{aligned} \langle \Delta , {\mathcal {W}}_{T_{\text {c}}}\Delta \rangle = -4\; \Lambda _1 \; \langle \Psi , W_h \Psi \rangle \end{aligned}$$

with $\Lambda _1$ in (3.21). Moreover, for any $T \geqslant T_0 > 0$ we have

4.156 $$\begin{aligned} |\langle \Delta , {\mathcal {W}}_T \Delta - {\mathcal {W}}_{T_{\text {c}}}\Delta \rangle | \leqslant C\; h^4 \; |T - {T_{\text {c}}}| \; \Vert V\alpha _*\Vert _2^2 \; \Vert \Psi \Vert _{H_{\text {mag}}^1(Q_h)}^2. \end{aligned}$$

##### Proof

Using the definition of $k_T(Z,r)$ in (4.152), we write

$$\begin{aligned} k_T(Z, r)&= - \frac{2}{\beta }\sum _{n\in {\mathbb {Z}}} \int _{{\mathbb {R}}^3} \frac{\text {d}p}{(2\pi )^3} \int _{{\mathbb {R}}^3} \frac{\text {d}q}{(2\pi )^3} \; \Bigl [ \frac{\text {e}^{\text {i}Z\cdot (p+q)} \text {e}^{\text {i}\frac{r}{2}\cdot (q-p)}}{(\text {i}\omega _n + \mu - p^2)^2 (\text {i}\omega _n - \mu + p^2)} \\&\quad - \frac{\text {e}^{\text {i}Z\cdot (p+q)} \text {e}^{\text {i}\frac{r}{2}\cdot (p-q)}}{(\text {i}\omega _n - \mu + p^2)^2 (\text {i}\omega _n + \mu - p^2)}\Bigr ] \end{aligned}$$

as well as

$$\begin{aligned} \int _{{\mathbb {R}}^3} \text {d}Z \; k_T(Z, r)&= -\frac{4}{\beta }\sum _{n\in {\mathbb {Z}}} \int _{{\mathbb {R}}^3} \frac{\text {d}p}{(2\pi )^3} \; \text {e}^{\text {i}r \cdot p} \, \frac{p^2 -\mu }{(\text {i}\omega _n + \mu - p^2)^2 (\text {i}\omega _n - \mu + p^2)^2}. \end{aligned}$$

With the Mittag-Leffler series expansion in (4.35), we also check that

4.157 $$\begin{aligned} \frac{\beta }{2} \frac{1}{\cosh ^2(\frac{\beta }{2}z)} = \frac{\text {d}}{\text {d}z} \tanh \bigl ( \frac{\beta }{2} z\bigr ) = - \frac{2}{\beta } \sum _{n\in {\mathbb {Z}}} \frac{1}{(\text {i}\omega _n - z)^2} \end{aligned}$$

holds. We use (4.157) and the partial fraction expansion

$$\begin{aligned} \frac{1}{(\text {i}\omega _n - E)^2(\text {i}\omega _n + E)^2} = \frac{1}{4E^2} \Bigl [ \frac{1}{(\text {i}\omega _n - E)^2} + \frac{1}{(\text {i}\omega _n + E)^2}\Bigr ] - \frac{1}{4E^3} \Bigl [ \frac{1}{\text {i}\omega _n - E} - \frac{1}{\text {i}\omega _n + E}\Bigr ] \end{aligned}$$

to see that

$$\begin{aligned} \frac{4}{\beta } \sum _{n\in {\mathbb {Z}}} \frac{E}{(\text {i}\omega _n - E)^2(\text {i}\omega _n + E)^2} = \beta ^2 \; g_1(\beta E) \end{aligned}$$

with the function $g_1$ in (3.19). Therefore,

$$\begin{aligned} \langle \Delta , M_{T_{\text {c}}}^W \Delta \rangle&= -{\beta _{\text {c}}}^2 \int _{{\mathbb {R}}^3} \frac{\text {d}p}{(2\pi )^3} \; |(-2)\widehat{V\alpha _*}(p)|^2 \, g_1({\beta _{\text {c}}}(p^2-\mu )) \; \langle \Psi , W_h \Psi \rangle \\&= - 4\, \Lambda _1 \, \langle \Psi , W_h\Psi \rangle . \end{aligned}$$

This proves (4.155).

To obtain the bound in (4.156), we argue as in the proof of (4.124). $\square$

#### Summary: the quadratic terms

In this section, we summarize our results concerning the quadratic terms (in $\Delta$) that are relevant for the proof of Theorem 3.6. We also use our results to prove another statement (Proposition 4.26 below), which will later be used in the proof of Proposition 3.7. We start by summarizing our findings.

Let the assumptions of Theorem 3.6 hold and recall the definition of ${\mathcal {R}}_{T, {{\textbf {A}}}, W}^{(2)}$ in (4.44). An application of Hölder’s inequality in (2.1) and the bound $\Vert ( \text {i}\omega _n - {\mathfrak {h}}_{{\textbf {A}}})^{-1} \Vert _{\infty } \leqslant |\omega _n|^{-1}$ show that

$$\begin{aligned} | \langle \Delta , {\mathcal {R}}_{T, {{\textbf {A}}}, W}^{(2)}\Delta \rangle | \leqslant C \; \Vert \Delta \Vert _2^2 \; \Vert W_h \Vert ^2_{\infty } \leqslant C \; h^4 \; \Vert \Psi \Vert _{2}^2 \leqslant C \; h^6 \; \Vert \Psi \Vert _{H_{\text {mag}}^2(Q_h)}^2. \end{aligned}$$

We combine (4.42), this bound, and the results of Propositions 4.17, 4.19, 4.20, 4.23, 4.24, and 4.25, to see that for $T = {T_{\text {c}}}(1-Dh^2)$ with $D \in {\mathbb {R}}$ the identity

4.158 $$\begin{aligned}&-\frac{1}{4} \langle \Delta , L_{T,{{\textbf {A}}}, W} \Delta \rangle + \Vert \Psi \Vert _2^2 \, \langle \alpha _*, V \alpha _* \rangle \nonumber \\&\quad = \Lambda _0 \; \Vert \Pi _{{{\textbf {A}}}_h}\Psi \Vert _2^2 + \Lambda _1 \; \langle \Psi , W_h\Psi \rangle - Dh^2 \; \Lambda _2 \; \Vert \Psi \Vert _2^2 + R_2(\Delta ) \end{aligned}$$

holds. The remainder term $R_2(\Delta )$ obeys the estimate

$$\begin{aligned} | R_2(\Delta ) | \leqslant C\; \bigl ( h^5 \; \Vert \Psi \Vert _{H_{\text {mag}}^1(Q_h)}^2 + h^6 \; \Vert \Psi \Vert _{H_{\text {mag}}^2(Q_h)}^2\bigr ). \end{aligned}$$

This concludes the computation of the quadratic terms in the Ginzburg–Landau functional. It remains to compute the term that is proportional to $| \Psi |^4$, which is the content of the remaining part of Sect. 4.5.

Before we continue with the proof of Theorem 3.6, we state and prove the following statement, which will later be used in the proof of Proposition 3.7. It is a straightforward consequence of our results for the quadratic terms, and we therefore prove it here.

##### Proposition 4.26

Let $| \cdot |^k V\alpha _* \in L^2({\mathbb {R}}^3)$ for $k \in \{ 0,1,2 \}$, let $A\in W^{3,\infty }({\mathbb {R}}^3,{\mathbb {R}}^3)$ and $W \in W^{1,\infty }({\mathbb {R}}^3,{\mathbb {R}})$ be periodic, assume $\Psi \in H_{\text {mag}}^1(Q_h)$, and denote $\Delta \equiv \Delta _\Psi$ as in (3.1). For any $T_0>0$ there is $h_0>0$ such that for any $T\geqslant T_0$ and any $0 < h \leqslant h_0$ we have

4.159 $$\begin{aligned} - \frac{1}{4} \langle \Delta , L_{T,{{\textbf {A}}}, W} \Delta \rangle + \Vert \Psi \Vert _2^2 \; \langle \alpha _*, V\alpha _*\rangle&\leqslant c \, \frac{T - {T_{\text {c}}}}{{T_{\text {c}}}}\, \Vert \Psi \Vert _2^2 + C h^4 \, \Vert \Psi \Vert _{H_{\text {mag}}^1(Q_h)}^2. \end{aligned}$$

##### Proof

We write

4.160 $$\begin{aligned} -\frac{1}{4} \langle \Delta , L_{T, {{\textbf {A}}}, W} \Delta \rangle = - \frac{1}{4} \langle \Delta , L_{T,{{\textbf {A}}}} \Delta \rangle - \frac{1}{4} \langle \Delta , L_{T, {{\textbf {A}}}, W} \Delta - L_{T, {{\textbf {A}}}} \Delta \rangle \end{aligned}$$

and use the resolvent identity in (4.36) to write one of the operators on the right side as

4.161 $$\begin{aligned} L_{T, {{\textbf {A}}}, W} \Delta - L_{T, {{\textbf {A}}}} \Delta&= -\frac{2}{\beta }\sum _{n\in {\mathbb {Z}}} \frac{1}{\text {i}\omega _n - {\mathfrak {h}}_{{\textbf {A}}}} W_h \frac{1}{\text {i}\omega _n - {\mathfrak {h}}_{{{\textbf {A}}}, W}} \Delta \frac{1}{\text {i}\omega _n + \overline{{\mathfrak {h}}_{{{\textbf {A}}}, W}}} \nonumber \\&\quad - \frac{1}{\text {i}\omega _n - {\mathfrak {h}}_{{\textbf {A}}}} \Delta \frac{1}{\text {i}\omega _n + \overline{{\mathfrak {h}}_{{{\textbf {A}}}, W}}} W_h \frac{1}{\text {i}\omega _n + \overline{{\mathfrak {h}}_{{\textbf {A}}}}}. \end{aligned}$$

An application of Hölder’s inequality therefore implies the bound

$$\begin{aligned} |\langle \Delta , L_{T, {{\textbf {A}}}, W} \Delta - L_{T, {{\textbf {A}}}} \Delta \rangle | \leqslant C \, h^4 \, \Vert \Psi \Vert _{H_{\text {mag}}^1(Q_h)}^2. \end{aligned}$$

When we additionally use the decomposition of $L_{T,{{\textbf {A}}}}$ in (4.49), Propositions 4.9, 4.11, and 4.15, we find

4.162 $$\begin{aligned}&- \frac{1}{4} \langle \Delta , L_{T,{{\textbf {A}}}} \Delta \rangle + \Vert \Psi \Vert _2^2 \; \langle \alpha _*, V\alpha _*\rangle \nonumber \\&\quad \qquad \qquad = - \frac{1}{4} \langle \Delta , M_T^{(1)}\Delta - M_{{T_{\text {c}}}}^{(1)} \Delta \rangle - \frac{1}{4} \langle \Delta , M_{T,{{\textbf {A}}}} \Delta - M_T^{(1)}\Delta \rangle + R_1(\Delta ), \end{aligned}$$

with a remainder $R_1(\Delta )$ obeying the bound

$$\begin{aligned} |R_1(\Delta ) | \leqslant C \; h^5 \; \Vert \Psi \Vert _{H_{\text {mag}}^1(Q_h)}^2. \end{aligned}$$

From Proposition 4.17 we know that

$$\begin{aligned} -\frac{1}{4} \langle \Delta , M_T^{(1)}\Delta - M_{{T_{\text {c}}}}^{(1)} \Delta \rangle \leqslant c \; \frac{T - {T_{\text {c}}}}{{T_{\text {c}}}} \; \Vert \Psi \Vert _2^2. \end{aligned}$$

We also claim that the bound

4.163 $$\begin{aligned} |\langle \Delta , M_{T,{{\textbf {A}}}}\Delta - M_T^{(1)}\Delta \rangle |&\leqslant C\; h^4 \; \Vert V\alpha _*\Vert _2^2 \; \Vert \Psi \Vert _{H_{\text {mag}}^1(Q_h)}^2 \end{aligned}$$

holds. Its proof can be achieved with the same methods that have been used to prove Proposition 4.15. The main point is that we have to use the bound

4.164 $$\begin{aligned} |\langle \Psi , [\cos (Z\cdot \Pi _{{\textbf {A}}}) - 1] \Psi \rangle |&\leqslant C\; h^4 \; |Z|^2 \; \Vert \Psi \Vert _{H_{\text {mag}}^1(Q_h)}^2, \end{aligned}$$

as well as the operator inequality in (4.138) for $(Z\cdot \Pi _{{\textbf {A}}})^2$. Since no additional difficulties occur, we leave the details to the reader. This completes the proof of (4.159). $\square$

#### A representation formula for the operator $N_{T, {{\textbf {A}}}}$

In this and the following sections we investigate the nonlinear operator

4.165 $$\begin{aligned} N_{T, {{\textbf {A}}}} {:}{=}N_{T, {{\textbf {A}}}, 0} \end{aligned}$$

with $N_{T, {{\textbf {A}}}, W}$ in (3.12). In particular, we show that the quartic term in the GL functional emerges from $\langle \Delta , N_{T, {{\textbf {A}}}} (\Delta ) \rangle$. In Sect. 4.5.12 we show that $\langle \Delta , N_{T, {{\textbf {A}}}, W}(\Delta ) - N_{T, {{\textbf {A}}}} (\Delta ) \rangle$ yields a negligible contribution. In this section we will also collect all previous results and finish the proof of Theorem 3.6.

Before we state a representation formula for $N_{T, {{\textbf {A}}}}$ in terms of relative and center-of-mass coordinates, we introduce the notation ${{\textbf {Z}}}$ to denote the vector $(Z_1,Z_2,Z_3)$ with $Z_1, Z_2, Z_3 \in {\mathbb {R}}^3$ as well as $\text {d}{{\textbf {Z}}}= \text {d}Z_1 \text {d}Z_2 \text {d}Z_3$.

##### Lemma 4.27

The operator $N_{T, {{\textbf {A}}}}$ in (3.12) acts as

$$\begin{aligned} N_{T, {{\textbf {A}}}}(\alpha ) (X, r)&= \iiint _{{\mathbb {R}}^9} \text {d}{{\textbf {Z}}}\iiint _{{\mathbb {R}}^9} \text {d}{{\textbf {s}}}\; \ell _{T, {{\textbf {A}}}}(X, {{\textbf {Z}}}, r, {{\textbf {s}}})\; {\mathcal {A}}(X, {{\textbf {Z}}}, {{\textbf {s}}}), \end{aligned}$$

where

4.166 $$\begin{aligned} {\mathcal {A}}(X, {{\textbf {Z}}}, {{\textbf {s}}})&{:}{=}\text {e}^{\text {i}Z_1\cdot (-\text {i}\nabla _X)} \alpha (X, s_1) \; \overline{\text {e}^{\text {i}Z_2\cdot (-\text {i}\nabla _X)} \alpha (X, s_2)} \; \text {e}^{\text {i}Z_3\cdot (-\text {i}\nabla _X)} \alpha (X,s_3) \end{aligned}$$

and

4.167 $$\begin{aligned} \ell _{T, {{\textbf {A}}}}(X, {{\textbf {Z}}}, r, {{\textbf {s}}})&{:}{=}\frac{2}{\beta } \sum _{n\in {\mathbb {Z}}} \ell _{T, {{\textbf {A}}}}^n(X, {{\textbf {Z}}}, r, {{\textbf {s}}}) \; \text {e}^{\text {i}\Upsilon _{{{\textbf {A}}}_h}(X, {{\textbf {Z}}}, r, s)}. \end{aligned}$$

Here,

4.168 $$\begin{aligned} \ell _{T, {{\textbf {A}}}}^n(X, {{\textbf {Z}}}, r, {{\textbf {s}}})&{:}{=}g_h^{\text {i}\omega _n}\biggl (X + \frac{r}{2} , \, X + Z_1 + \frac{s_1}{2}\biggr ) \, g_h^{-\text {i}\omega _n} \biggl ( X + Z_2 + \frac{s_2}{2} , \, X + Z_1 - \frac{s_1}{2}\biggr ) \nonumber \\&\quad \times g_h^{\text {i}\omega _n}\biggl ( X + Z_2 - \frac{s_2}{2} , \, X + Z_3 + \frac{s_3}{2}\biggr ) \, g_h^{-\text {i}\omega _n}\biggl (X - \frac{r}{2} , \, X + Z_3 - \frac{s_3}{2}\biggr ), \end{aligned}$$

with $g_h^z$ in (4.3) and

4.169 $$\begin{aligned} \Upsilon _{{\textbf {A}}}(X, {{\textbf {Z}}}, r, {{\textbf {s}}})&{:}{=}\Phi _{{\textbf {A}}}\left( X + \frac{r}{2} , \, X + Z_1 + \frac{s_1}{2}\right) + \Phi _{{\textbf {A}}}\left( X + Z_2 + \frac{s_2}{2} , \, X + Z_1 - \frac{s_1}{2}\right) \nonumber \\&\quad + \Phi _{{\textbf {A}}}\left( X + Z_2 - \frac{s_2}{2} , \, X + Z_3 + \frac{s_3}{2}\right) + \Phi _{{\textbf {A}}}\left( X - \frac{r}{2} \, , \, X + Z_3 - \frac{s_3}{2}\right) . \end{aligned}$$

##### Remark 4.28

The above representation formula for $N_{T,{{\textbf {A}}}}$ should be compared to that in the case of a constant magnetic field in [15, Lemma 4.16] and to the representation formula for $L_{T,{{\textbf {A}}}}$ in 4.7. The following two properties are relevant for us: (a) The functions $\alpha$ are multiplied by translation operators that can later be completed with appropriate phase factors to give magnetic translation operators. (b) The coordinates appearing in $\Upsilon _{{{\textbf {A}}}}$ in (4.169) equal those in the different factors in the definition of $\ell _{T, {{\textbf {A}}}}^n(X, {{\textbf {Z}}}, r, {{\textbf {s}}})$ in (4.168). When proving bounds, this allows us to find a similar structure of nested convolutions as the one we already encountered in the analysis of $L_{T,{{\textbf {A}}}}$. The center-of-mass part of $\alpha$ never participates in these convolutions.

##### Proof of Lemma 4.27

When we compute the integral kernel of $N_{T, {{\textbf {A}}}}$ using (4.40) in the case $W =0$, we get

4.170 $$\begin{aligned} N_{T, {{\textbf {A}}}} (\alpha )(x,y)&= \smash {\frac{2}{\beta }\sum _{n\in {\mathbb {Z}}} \iiint _{{\mathbb {R}}^{9}} \text {d}{{\textbf {u}}} \iiint _{{\mathbb {R}}^9}\text {d}{{\textbf {v}}}} \; G_h^{\text {i}\omega _n} (x, u_1)\, \alpha (u_1,v_1)\, G_h^{-\text {i}\omega _n} (u_2,v_1) \, \overline{\alpha (u_2,v_2)}\nonumber \\ \nonumber \\&\quad \times G_h^{\text {i}\omega _n}(v_2,u_3)\, \alpha (u_3,v_3)\, G_h^{-\text {i}\omega _n}(y, v_3), \end{aligned}$$

We highlight that, by a slight abuse of notation, $\alpha$ and $N_{T, {{\textbf {A}}}} (\alpha )$ in the above equation are functions of the original coordinates, while they are functions of relative and center-of-mass coordinates in (4.166). Let us denote $X = \frac{x+y}{2}$, $r=x-y$ and let us also introduce the relative coordinate ${{\textbf {s}}}$ and the center-of-mass coordinate ${{\textbf {Z}}}$ by

$$\begin{aligned} u_i&= X + Z_i + \frac{s_i}{ 2 },&v_i&= X + Z_i - \frac{s_i}{ 2 },&i&=1,2,3. \end{aligned}$$

When we express the integration in (4.170) in terms of these coordinates and use (4.3), we see that the claimed formula holds. $\square$

As the operator $L_{T,{{\textbf {A}}}}$, we analyze the operator $N_{T, {{\textbf {A}}}}$ in four steps. More precisely, we decompose $N_{T, {{\textbf {A}}}}$ as

4.171 $$\begin{aligned} N_{T, {{\textbf {A}}}} = (N_{T, {{\textbf {A}}}} - {\widetilde{N}}_{T, {{\textbf {A}}}}) + (\widetilde{N}_{T, {{\textbf {A}}}} - N_{T, {{\textbf {B}}}}^{(1)}) + (N_{T, {{\textbf {B}}}}^{(1)} - N_T^{(2)}) + N_{T}^{(2)} \end{aligned}$$

with ${\widetilde{N}}_{T, {{\textbf {A}}}}$ defined below in (4.172), $N_{T, {{\textbf {B}}}}^{(1)}$ in (4.181), and $N_T^{(2)}$ in (4.190). To obtain the map ${\widetilde{N}}_{T, {{\textbf {A}}}}$ from $N_{T, {{\textbf {A}}}}$ we need to replace $g_{h}^z$ by $g_0^z$. The operator $N_{T,{{\textbf {B}}}}^{(1)}$ emerges when we use a part of the phase $\exp (\text {i}\Upsilon _{{{\textbf {A}}}_h}(X, {{\textbf {Z}}}, r, s))$ in the definition of $\ell _{T, {{\textbf {A}}}}$ in (4.167) to replace the translation operators $\exp (\text {i}Z\cdot P_X)$ in front the $\alpha$ factors by magnetic translation operators. The part of the phase factor that is not needed during this procedure is shown to yield a negligible contribution. Finally, the operator $N_T^{(2)}$ is obtained when we replace the just found magnetic translations by 1. The above decomposition of $N_{T, {{\textbf {A}}}}$ should be compared to that in [15, Eq. (4.120)]. In Sect. 4.5.10 we show that the terms in brackets in (4.171) only yield negligible contributions. Afterwards, we extract in Sect. 4.5.11 the quartic term in the Ginzburg–Landau functional from $\langle \Delta , N_{T}^{(2)}(\Delta ) \rangle$. In Sect. 4.5.12 we summarize our findings.

#### Approximation of $N_{T, {{\textbf {A}}}}$

**4.5.10.1 The operator **${\widetilde{N}}_{T, {{\textbf {A}}}}$. We define the operator ${\widetilde{N}}_{T, {{\textbf {A}}}}$ by

4.172 $$\begin{aligned} {\widetilde{N}}_{T, {{\textbf {A}}}}(\alpha ) (X,r)&{:}{=}\iiint _{{\mathbb {R}}^{9}} \text {d}{{\textbf {Z}}}\iiint _{{\mathbb {R}}^{9}} \text {d}{{\textbf {s}}}\; {\widetilde{\ell }}_{T, {{\textbf {A}}}} (X, {{\textbf {Z}}}, r, {{\textbf {s}}}) \; {\mathcal {A}}(X, {{\textbf {Z}}}, {{\textbf {s}}}) \end{aligned}$$

with ${\mathcal {A}}$ in (4.166) and

$$\begin{aligned} {\widetilde{\ell }}_{T, {{\textbf {A}}}}(X, {{\textbf {Z}}}, r, {{\textbf {s}}})&{:}{=}\frac{2}{\beta }\sum _{n\in {\mathbb {Z}}} \ell _T^n ({{\textbf {Z}}}, r, {{\textbf {s}}}) \; \text {e}^{\text {i}\Upsilon _{{{\textbf {A}}}_h}(X, {{\textbf {Z}}}, r, {{\textbf {s}}})}, \end{aligned}$$

where $\Upsilon _{{\textbf {A}}}$ has been defined in (4.169), and

4.173 $$\begin{aligned} \ell _T^n({{\textbf {Z}}}, r, {{\textbf {s}}})&{:}{=}g_0^{\text {i}\omega _n} \biggl (Z_1 - \frac{r-s_1}{2}\biggr ) \; g_0^{-\text {i}\omega _n} \biggl ( Z_1 - Z_2 - \frac{s_1 + s_2}{2}\biggr ) \nonumber \\&\quad \times g_0^{\text {i}\omega _n} \biggl ( Z_2 - Z_3 - \frac{s_2 + s_3}{2} \biggr ) \; g_0^{-\text {i}\omega _n} \biggl ( Z_3 + \frac{r-s_3}{2}\biggr ). \end{aligned}$$

In our calculation of the BCS energy we can replace $N_{T, {{\textbf {A}}}}(\Delta )$ by ${\widetilde{N}}_{T, {{\textbf {A}}}}(\Delta )$ because of the following error bound.

##### Proposition 4.29

Let , let $A\in W^{3,\infty }({\mathbb {R}}^3,{\mathbb {R}}^3)$ be periodic, assume that $\Psi \in H_{\text {mag}}^1(Q_h)$, and denote $\Delta \equiv \Delta _\Psi$ as in (3.1). For any $T_0>0$ there is $h_0>0$ such that for any $T\geqslant T_0$ and any $0 < h \leqslant h_0$ we have

The function

4.174 $$\begin{aligned} J_{T,{{\textbf {A}}}}&{:}{=}\smash {\frac{2}{\beta }\sum _{n\in {\mathbb {Z}}}} \; \tau ^{\text {i}\omega _n} * \rho ^{-\text {i}\omega _n} * \rho ^{\text {i}\omega _n} * \rho ^{-\text {i}\omega _n} + |g_0^{\text {i}\omega _n}| * \tau ^{-\text {i}\omega _n} * \rho ^{\text {i}\omega _n} * \rho ^{-\text {i}\omega _n} \nonumber \\\nonumber \\&\quad + |g_0^{\text {i}\omega _n}| * |g_0^{-\text {i}\omega _n} | * \tau ^{\text {i}\omega _n} * \rho ^{-\text {i}\omega _n} + |g_0^{\text {i}\omega _n}| * |g_0^{-\text {i}\omega _n} | * |g_0^{\text {i}\omega _n}| * \tau ^{-\text {i}\omega _n}. \end{aligned}$$

plays a prominent role in the proof of Proposition 4.29. Using Lemmas 4.2 and 4.4 as well as (4.56), we see that for any $T \geqslant T_0 > 0$ there is a constant $C>0$ such that

4.175 $$\begin{aligned} \Vert J_{T, {{\textbf {A}}}_h}\Vert _1 \leqslant C \; h^3 \end{aligned}$$

holds.

##### Proof of Proposition 4.29

The function $|\Psi |$ is periodic, and hence (2.9) implies

4.176 $$\begin{aligned} \Vert \text {e}^{\text {i}Z \cdot (-\text {i}\nabla _X)}\Psi \Vert _6^2 = \Vert \Psi \Vert _6^2 \leqslant C\, h^2 \, \Vert \Psi \Vert _{H_{\text {mag}}^1(Q_h)}^2. \end{aligned}$$

In particular, we have4.177as well as

4.178 $$\begin{aligned}&|\langle \Delta , N_{T, {{\textbf {A}}}}(\Delta )- {\widetilde{N}}_{T, {{\textbf {A}}}}(\Delta )\rangle | \nonumber \\&\quad \leqslant C\, h^4 \, \Vert \Psi \Vert _{H_{\text {mag}}^1(Q_h))}^4 \int _{{\mathbb {R}}^3} \text {d}r \, \iiint _{{\mathbb {R}}^9} \text {d}{{\textbf {s}}}\; |V\alpha _*(r)|\; |V\alpha _*(s_1)| \; |V\alpha _*(s_2)| \; |V\alpha _*(s_3)| \nonumber \\&\qquad \qquad \qquad \qquad \qquad \qquad \times \iiint _{{\mathbb {R}}^9} \text {d}{{\textbf {Z}}}\; \mathop {\mathrm {ess \, sup}}\limits _{X\in {\mathbb {R}}^3} \bigl |(\ell _{T, {{\textbf {A}}}} - {\widetilde{\ell }}_{T, {{\textbf {A}}}})(X, {{\textbf {Z}}}, r, {{\textbf {s}}})\bigr |. \end{aligned}$$

Next, we define the variables $Z_1',Z_2',Z_3'$ via the equation

4.179 $$\begin{aligned} Z_1' - Z_2'&{:}{=}Z_1 - Z_2 - \frac{s_1 +s_2}{2},&Z_2' - Z_3'&{:}{=}Z_2 - Z_3 - \frac{s_2 + s_3}{2},&Z_3'&{:}{=}Z_3 + \frac{r-s_3}{2}, \end{aligned}$$

which implies

4.180 $$\begin{aligned} Z_1 - \frac{r-s_1}{2} = Z_1' - (r - s_1 - s_2 - s_3). \end{aligned}$$

We argue as in the proof of (4.65) to see that

$$\begin{aligned} \iiint _{{\mathbb {R}}^9} \text {d}{{\textbf {Z}}}\; \mathop {\mathrm {ess \, sup}}\limits _{X\in {\mathbb {R}}^3} \bigl |(\ell _{T, {{\textbf {A}}}} - {\widetilde{\ell }}_{T, {{\textbf {A}}}})(X, {{\textbf {Z}}}, r, {{\textbf {s}}})\bigr | \leqslant J_{T, {{\textbf {A}}}_h}(r - s_1 - s_2 - s_3) \end{aligned}$$

holds with $J_{T, {{\textbf {A}}}}$ in (4.174). When insert this bound into (4.178) and useas well as (4.175), this finishes the proof. $\square$

**4.5.10.2 The operator **$N_{T, {{\textbf {B}}}}^{(1)}$. We define the operator $N_{T, {{\textbf {B}}}}^{(1)}$ by

4.181 $$\begin{aligned} N_{T, {{\textbf {B}}}}^{(1)}(\alpha )(X,r)&{:}{=}\iiint _{{\mathbb {R}}^9} \text {d}{{\textbf {Z}}}\iiint _{{\mathbb {R}}^9} \text {d}{{\textbf {s}}}\; \ell _T ({{\textbf {Z}}}, r, {{\textbf {s}}}) \; {\mathcal {A}}_{{\textbf {B}}}(X, {{\textbf {Z}}}, {{\textbf {s}}}), \end{aligned}$$

where

4.182 $$\begin{aligned} \ell _T({{\textbf {Z}}}, r, {{\textbf {s}}})&{:}{=}\ell _{T,0}(0, {{\textbf {Z}}}, r, {{\textbf {s}}}), \end{aligned}$$

with $\ell _{T,0}$ in (4.167) and

4.183 $$\begin{aligned} {\mathcal {A}}_{{\textbf {B}}}(X, {{\textbf {Z}}}, {{\textbf {s}}})&{:}{=}\text {e}^{\text {i}Z_1\cdot \Pi } \alpha (X, s_1) \; \overline{\text {e}^{\text {i}Z_2\cdot \Pi } \alpha (X, s_2)} \; \text {e}^{\text {i}Z_3\cdot \Pi } \alpha (X,s_3). \end{aligned}$$

The following bound allows us to replace $\langle \Delta , \widetilde{N}_{T, {{\textbf {A}}}}(\Delta ) \rangle$ by $\langle \Delta , N_{T, {{\textbf {B}}}}^{(1)}(\Delta ) \rangle$ in our computation of the energy.

##### Proposition 4.30

Let  for $k\in \{0,1\}$, let $A\in W^{1,\infty }({\mathbb {R}}^3,{\mathbb {R}}^3)$ be periodic, assume $\Psi \in H_{\text {mag}}^1(Q_h)$, and denote $\Delta \equiv \Delta _\Psi$ as in (3.1). For any $T_0>0$ there is $h_0>0$ such that for any $T\geqslant T_0$ and any $0 < h \leqslant h_0$ we have

Before we give the proof of Proposition 4.30 we define the functions

4.184 $$\begin{aligned} J_T^{(1)}&{:}{=}\smash {\frac{2}{\beta }\sum _{n\in {\mathbb {Z}}}} \, |g_0^{\text {i}\omega _n}| * \bigl (|\cdot |\, |g_0^{-\text {i}\omega _n}|\bigr ) * \bigl (|\cdot |\, |g_0^{\text {i}\omega _n}|\bigr ) * |g_0^{-\text {i}\omega _n}| \nonumber \\\nonumber \\&\quad + |g_0^{\text {i}\omega _n}| * \bigl (|\cdot |\, |g_0^{-\text {i}\omega _n}|\bigr ) * |g_0^{\text {i}\omega _n}| * \bigl (|\cdot |\, |g_0^{-\text {i}\omega _n}|\bigr ) \nonumber \\&\quad + |g_0^{\text {i}\omega _n}| * |g_0^{-\text {i}\omega _n}| * \bigl (|\cdot |\, |g_0^{\text {i}\omega _n}|\bigr ) * \bigl (|\cdot |\, |g_0^{-\text {i}\omega _n}|\bigr ) \end{aligned}$$

and

4.185 $$\begin{aligned} J_T^{(2)}&{:}{=}\smash {\frac{2}{\beta }\sum _{n\in {\mathbb {Z}}}} \, \bigl ( |\cdot | \, |g_0^{\text {i}\omega _n}|\bigr ) * |g_0^{-\text {i}\omega _n}| * |g_0^{\text {i}\omega _n}| * |g_0^{-\text {i}\omega _n}| + |g_0^{\text {i}\omega _n}| * \bigl (|\cdot |\, |g_0^{-\text {i}\omega _n}|\bigr ) * |g_0^{\text {i}\omega _n}| * |g_0^{-\text {i}\omega _n}| \nonumber \\\nonumber \\&\quad + |g_0^{\text {i}\omega _n}| * |g_0^{-\text {i}\omega _n}| * \bigl (|\cdot |\, |g_0^{\text {i}\omega _n}|\bigr ) * |g_0^{-\text {i}\omega _n}| + |g_0^{\text {i}\omega _n}| * |g_0^{-\text {i}\omega _n}| * |g_0^{\text {i}\omega _n}| * \bigl (|\cdot |\, |g_0^{-\text {i}\omega _n}|\bigr ). \end{aligned}$$

Using Lemma 4.4 and (4.56), we show that for any $T_0 > 0$ there is a constant $C>0$ such that for $T \geqslant T_0$ we have

4.186 $$\begin{aligned} \Vert J_T^{(1)} \Vert _1 + \Vert J_T^{(2)} \Vert _1 \leqslant C. \end{aligned}$$

##### Proof of Proposition 4.30

We recall the definition of the phase $\Upsilon _{{\textbf {A}}}$ in (4.169). A tedious but straightforward computation shows that

$$\begin{aligned} \Upsilon _{{{\textbf {A}}}_{{{\textbf {B}}}}} (X, {{\textbf {Z}}}, r, {{\textbf {s}}})&= \,Z_1 \cdot ({{\textbf {B}}}\wedge X) - Z_2 \cdot ({{\textbf {B}}}\wedge X) + Z_3 \cdot ({{\textbf {B}}}\wedge X) + \frac{{{\textbf {B}}}}{2} \cdot I({{\textbf {Z}}}, r, {{\textbf {s}}}), \end{aligned}$$

where

4.187 $$\begin{aligned} I({{\textbf {Z}}}, r, {{\textbf {s}}})&{:}{=}\, \frac{r}{2} \wedge \biggl ( Z_1 - \frac{r - s_1}{2}\biggr ) + \frac{r}{2} \wedge \biggl ( Z_3 + \frac{r-s_3}{2}\biggr ) \nonumber \\&\quad + \biggl ( Z_2 - Z_3 - \frac{s_2 + s_3}{2}\biggr ) \wedge \biggl ( Z_1 - Z_2 - \frac{s_1 + s_2}{2}\biggr ) \nonumber \\&\quad + \biggl ( Z_3 + \frac{r - s_3}{2}\biggr ) \wedge \biggl ( Z_1 - Z_2 - \frac{s_1 + s_2}{2}\biggr )+ \biggl ( s_2 + s_3 - \frac{r}{2}\biggr ) \wedge \biggl ( Z_1 - Z_2 - \frac{s_1 + s_2}{2}\biggr ) \nonumber \\&\quad + \biggl ( Z_3 + \frac{r - s_3}{2} \biggr ) \wedge \biggl ( Z_3 - Z_2 + \frac{s_2 + s_3}{2}\biggr )+ \biggl ( s_3 - \frac{r}{2}\biggr ) \wedge \biggl ( Z_3 - Z_2 + \frac{s_2 + s_3}{2}\biggr ). \end{aligned}$$

By (4.90), the operator ${\widetilde{N}}_{T, {{\textbf {A}}}}$ can therefore be rewritten as

$$\begin{aligned} {\widetilde{N}}_{T, {{\textbf {A}}}}(\alpha )&= \iiint _{{\mathbb {R}}^9} \text {d}{{\textbf {Z}}}\iiint _{{\mathbb {R}}^9} \text {d}s \; \ell _T({{\textbf {Z}}}, r, {{\textbf {s}}}) \, \text {e}^{\text {i}\Upsilon _{A_h}(X, {{\textbf {Z}}}, r, {{\textbf {s}}})} \text {e}^{\text {i}\frac{{{\textbf {B}}}}{2} \cdot I({{\textbf {Z}}}, r, {{\textbf {s}}})} \; {\mathcal {A}}_{{\textbf {B}}}(X, {{\textbf {Z}}}, {{\textbf {s}}}). \end{aligned}$$

This formula and the estimate in (4.177) imply the bound

4.188 $$\begin{aligned}&|\langle \Delta , {\widetilde{N}}_{T, {{\textbf {A}}}}(\Delta ) - N_{T, {{\textbf {B}}}}^{(1)}(\Delta )\rangle | \nonumber \\&\quad \leqslant C\; h^4 \, \Vert \Psi \Vert _{H_{\text {mag}}^1(Q_h)}^4 \int _{{\mathbb {R}}^3} \text {d}r \iiint _{{\mathbb {R}}^9} \text {d}{{\textbf {s}}}\; |V\alpha _*(r)| \; |V\alpha _*(s_1)| \; |V\alpha _*(s_2)| \; |V\alpha _*(s_3)| \nonumber \\&\qquad \times \frac{2}{\beta } \sum _{n\in {\mathbb {Z}}} \iiint _{{\mathbb {R}}^9} \text {d}{{\textbf {Z}}}\; |\ell _T^n({{\textbf {Z}}}, r, {{\textbf {s}}})| \; \sup _{X\in {\mathbb {R}}^3} \bigl | \text {e}^{\text {i}\Upsilon _{A_h}(X, {{\textbf {Z}}}, r, {{\textbf {s}}})} \, \text {e}^{\text {i}\frac{{{\textbf {B}}}}{2} \cdot I({{\textbf {Z}}}, r,{{\textbf {s}}})} - 1\bigr | \end{aligned}$$

with $\Upsilon _A$ in (4.169) and *I* in (4.187). In terms of the coordinates in (4.179) and with the help of (4.180), the phase function *I* can be written as

4.189 $$\begin{aligned} I({{\textbf {Z}}}, r,{{\textbf {s}}})&= (Z_2' - Z_3') \wedge (Z_1' - Z_2') + Z_3' \wedge (Z_1' - Z_2') + Z_3' \wedge (Z_3'- Z_2') \nonumber \\&\quad + \frac{r}{2} \wedge \bigl ( Z_1' - (r - s_1 - s_2 - s_3)\bigr ) + \bigl ( s_2 + s_3 - \frac{r}{2}\bigr ) \wedge (Z_1 ' - Z_2')\nonumber \\&\quad + \bigl ( s_3 - \frac{r}{2}\bigr ) \wedge (Z_3' - Z_2') + \frac{r}{2} \wedge Z_3'. \end{aligned}$$

Moreover, using the definition of $\Phi _{{\textbf {A}}}$ in (4.2), the definition of $\Upsilon _{A}(X, {{\textbf {Z}}}, r, {{\textbf {s}}})$ in (4.169), (4.179), and (4.180), we obtain the bound

$$\begin{aligned} |\Upsilon _{A}(X, {{\textbf {Z}}}, r, {{\textbf {s}}})|&\leqslant \Vert A\Vert _\infty \bigl ( (Z_1' - (r - s_1 - s_2 - s_3)) + (Z_1' - Z_2') + (Z_2' - Z_3') + Z_3'\bigr ). \end{aligned}$$

In combination with (4.188), (4.189), and an argument that is similar to the one used to obtain (4.114), we find

$$\begin{aligned}&\frac{2}{\beta } \sum _{n\in {\mathbb {Z}}} \iiint _{{\mathbb {R}}^9} \text {d}{{\textbf {Z}}}\; |\ell _{T,0}^n({{\textbf {Z}}}, r, {{\textbf {s}}})| \; \bigl | \text {e}^{\text {i}\Upsilon _{A_h}(X, {{\textbf {Z}}}, r, {{\textbf {s}}})}\, \text {e}^{\text {i}\frac{{{\textbf {B}}}}{2} \cdot I({{\textbf {Z}}}, r, {{\textbf {s}}})} - 1\bigr | \\&\quad \leqslant Ch \; \bigl [ J_T^{(1)} (r - s_1 - s_2 - s_3) + J_T^{(2)}(r - s_1 - s_2 - s_3) \; \bigl (1 + |r| + |s_1| + |s_2| + |s_3|\bigr )\bigr ] \end{aligned}$$

with the functions $J_T^{(1)}$ and $J_T^{(2)}$ in (4.184) and (4.185), respectively. Accordingly, an application of Young’s inequality shows thatThe claim of the proposition follows when we apply (4.186) on the right side of the above equation. $\square$

**4.5.10.3 The operator **$N_T^{(2)}$. We define the operator $N_{T}^{(2)}$ by

4.190 $$\begin{aligned} N_T^{(2)}(\alpha ) (X, r)&{:}{=}\iiint _{{\mathbb {R}}^9} \text {d}{{\textbf {Z}}}\iiint _{{\mathbb {R}}^9} \text {d}{{\textbf {s}}}\; \ell _{T} ({{\textbf {Z}}}, r, {{\textbf {s}}}) \, \prod _{i=1}^3 \alpha (X,s_i) \end{aligned}$$

with $$\ell _{T}$$ in (4.182).

In the computation of the BCS energy we can replace $\langle \Delta , N_{T, {{\textbf {B}}}}^{(1)}(\Delta ) \rangle$ by $\langle \Delta , N_{T}^{(2)}(\Delta ) \rangle$ with the help of the following error bound. Its proof can be found in [15, Proposition 4.20]. We highlight that the $H_{\text {mag}}^2(Q_h)$-norm of $\Psi$ is needed once more.

##### Proposition 4.31

Assume that  for $k\in \{0,1,2\}$, let $\Psi \in H_{\text {mag}}^2(Q_h)$, and $\Delta \equiv \Delta _\Psi$ as in (3.1). For any $T \geqslant T_0 >0$ there is $h_0 > 0$ such that for $0 < h \leqslant h_0$ we have

#### Calculation of the quartic term in the Ginzburg–Landau functional

The quartic term in the Ginzburg–Landau functional in (1.18) is contained in $\langle \Delta , N_T^{(2)}(\Delta ) \rangle$. It can be extracted with the following proposition, whose proof can be found in [15, Proposition 4.21].

##### Proposition 4.32

Assume  and let $\Psi \in H_{\text {mag}}^1(Q_h)$ as well as $\Delta \equiv \Delta _\Psi$ as in (3.1). For any $h>0$, we have

$$\begin{aligned} \langle \Delta , N_{{T_{\text {c}}}}^{(2)}(\Delta )\rangle = 8\; \Lambda _3 \; \Vert \Psi \Vert _4^4 \end{aligned}$$

with $\Lambda _3$ in (3.23). Moreover, for any $T \geqslant T_0 > 0$, we have

#### Summary: the quartic term and proof of Theorem 3.6

Let the assumptions of Theorem 3.6 hold. We use the resolvent identity in (4.36) to decompose the operator $N_{T, {{\textbf {A}}}, W}$ in (3.12) as

4.191 $$\begin{aligned} N_{T, {{\textbf {A}}}, W} = N_{T, {{\textbf {A}}}} + {\mathcal {R}}_{T, {{\textbf {A}}},W}^{(3)} \end{aligned}$$

with $N_{T, {{\textbf {A}}}}$ in (4.165) and

4.192 $$\begin{aligned}&{\mathcal {R}}_{T, {{\textbf {A}}}, W}^{(3)}(\Delta ) \nonumber \\&\qquad {:}{=}\frac{2}{\beta }\sum _{n\in {\mathbb {Z}}} \Bigl [ \frac{1}{\text {i}\omega _n - {\mathfrak {h}}_{{\textbf {A}}}} \, W_h \, \frac{1}{\text {i}\omega _n -{\mathfrak {h}}_{{{\textbf {A}}}, W}} \, \Delta \, \frac{1}{\text {i}\omega _n + \overline{{\mathfrak {h}}_{{{\textbf {A}}}, W}}} \, \overline{\Delta }\, \frac{1}{\text {i}\omega _n - {\mathfrak {h}}_{{{\textbf {A}}}, W}} \, \Delta \, \frac{1}{\text {i}\omega _n + \overline{{\mathfrak {h}}_{{{\textbf {A}}}, W}}} \nonumber \\&\qquad \quad - \frac{1}{\text {i}\omega _n - {\mathfrak {h}}_{{\textbf {A}}}} \, \Delta \, \frac{1}{\text {i}\omega _n + \overline{{\mathfrak {h}}_{{\textbf {A}}}}} \, W_h \, \frac{1}{\text {i}\omega _n + \overline{{\mathfrak {h}}_{{{\textbf {A}}}, W}}} \, \overline{\Delta }\, \frac{1}{\text {i}\omega _n - {\mathfrak {h}}_{{{\textbf {A}}}, W}} \, \Delta \, \frac{1}{\text {i}\omega _n + \overline{{\mathfrak {h}}_{{{\textbf {A}}}, W}}} \nonumber \\&\qquad \quad + \frac{1}{\text {i}\omega _n - {\mathfrak {h}}_{{\textbf {A}}}} \, \Delta \, \frac{1}{\text {i}\omega _n + \overline{{\mathfrak {h}}_{{\textbf {A}}}}} \, \overline{\Delta }\, \frac{1}{\text {i}\omega _n - {\mathfrak {h}}_{{\textbf {A}}}} \, W_h \, \frac{1}{\text {i}\omega _n - {\mathfrak {h}}_{{{\textbf {A}}}, W}} \, \Delta \, \frac{1}{\text {i}\omega _n + \overline{{\mathfrak {h}}_{{{\textbf {A}}}, W}}} \nonumber \\&\qquad \quad - \frac{1}{\text {i}\omega _n - {\mathfrak {h}}_{{\textbf {A}}}} \, \Delta \, \frac{1}{\text {i}\omega _n + \overline{{\mathfrak {h}}_{{\textbf {A}}}}} \, \overline{\Delta }\, \frac{1}{\text {i}\omega _n - {\mathfrak {h}}_{{\textbf {A}}}} \, \Delta \, \frac{1}{\text {i}\omega _n + \overline{{\mathfrak {h}}_{{\textbf {A}}}}} \, W_h \, \frac{1}{\text {i}\omega _n + \overline{{\mathfrak {h}}_{{{\textbf {A}}}, W}}} \Bigr ]. \end{aligned}$$

We claim that the operator ${\mathcal {R}}_{T, {{\textbf {A}}}, W}^{(3)}$ satisfies the bound

$$\begin{aligned} \Vert {\mathcal {R}}_{T, {{\textbf {A}}}, W}^{(3)} (\Delta )\Vert _{{L^2(Q_h \times {\mathbb {R}}_{{\text {s}}}^3)}}&\leqslant C\, T^{-5} \, h^{5} \, \Vert \Psi \Vert _{H_{\text {mag}}^1(Q_h)}^3. \end{aligned}$$

This is a direct consequence of Hölder’s inequality in (2.1) for the trace per unit volume, which implies that the Hilbert–Schmidt norm per unit volume of the terms in the sum in (4.192) are bounded by $C\, |\omega _n|^{-5} \, \Vert W_h\Vert _\infty \, \Vert \Delta \Vert _6^3$. Moreover, an application of Lemma 4.1 and (2.9) show that this expression is bounded by $C\, |2n+1|^{-5} \, T^{-5} \, h^5 \Vert \Psi \Vert _{H_{\text {mag}}^1(Q_h)}^3$, which proves our claim.

When we combine this bound, Lemma 4.27, and Propositions 4.29-4.32, we find

4.193 $$\begin{aligned} \frac{1}{8} \langle \Delta , N_{T, {{\textbf {A}}}, W}(\Delta ) \rangle = \; \Lambda _3 \; \Vert \Psi \Vert _4^4 + R_4(h), \end{aligned}$$

where the remainder *R*(*h*) satisfies the bound

$$\begin{aligned} | R_4(h) | \leqslant C \; \Vert \Psi \Vert _{H_{\text {mag}}^1(Q_h)}^3 \, \bigl ( h^5 \; \Vert \Psi \Vert _{H_{\text {mag}}^1(Q_h)} + h^6 \; \Vert \Psi \Vert _{H_{\text {mag}}^2(Q_h)}\bigr ). \end{aligned}$$

The statement in Theorem 3.6 is a direct consequence of (4.193) and (4.158).

### Proof of Proposition 3.2

We assume that the assumptions of Proposition 3.2 hold and recall the definition of $\Gamma _\Delta$ in (3.4). Using the resolvent equation in (4.36) and (4.37), we write $\alpha _\Delta = [\Gamma _\Delta ]_{12}$ as

$$\begin{aligned} \alpha _\Delta&= [{\mathcal {O}}]_{12} + {\mathcal {R}}_{T, {{\textbf {A}}}, W}^{(4)}(\Delta ). \end{aligned}$$

Here, ${\mathcal {O}}= \frac{1}{\beta }\sum _{n\in {\mathbb {Z}}} \frac{1}{ \text {i}\omega _n - H_0} \delta \frac{1}{ \text {i}\omega _n - H_0}$, see (4.38), with $\delta$ in (3.1) and

4.194 $$\begin{aligned} {\mathcal {R}}_{T,{{\textbf {A}}}, W}^{(4)}(\Delta )&{:}{=}\frac{1}{\beta }\sum _{n\in {\mathbb {Z}}} \Bigl [ \frac{1}{ \text {i}\omega _n - H_0} \delta \frac{1}{ \text {i}\omega _n - H_0} \delta \frac{1}{ \text {i}\omega _n - H_\Delta } \delta \frac{1}{ \text {i}\omega _n - H_0}\Bigr ]_{12}. \end{aligned}$$

Moreover, we have $[{\mathcal {O}}]_{12} = -\frac{1}{2} L_{T, {{\textbf {A}}}, W}\Delta$ with $L_{T, {{\textbf {A}}}, W}$ in (3.11). Using the decomposition of $L_{T, {{\textbf {A}}}, W}$ in (4.42), we define

4.195 $$\begin{aligned} \eta _0(\Delta )&{:}{=}\frac{1}{2} \bigl ( L_{T, {{\textbf {A}}}, W} \Delta - L_{T, {{\textbf {A}}}}\Delta \bigr ) + \frac{1}{2} \bigl (L_{T, {{\textbf {A}}}}\Delta - M_{T, {{\textbf {A}}}}\Delta \bigr ) + \frac{1}{2} \bigl ( M_T^{(1)}\Delta - M_{{T_{\text {c}}}}^{(1)}\Delta \bigr ) \nonumber \\&\quad + \frac{1}{2} \bigl ( M_{T, {{\textbf {A}}}}\Delta - M_{T, {{\textbf {A}}}_{e_3}}\Delta \bigr ) + {\mathcal {R}}_{T, {{\textbf {A}}}, W}^{(4)}(\Delta ), \nonumber \\ \eta _\perp (\Delta )&{:}{=}\, \frac{1}{2} \bigl ( M_{T, {{\textbf {A}}}_{e_3}}\Delta - M_{T}^{(1)}\Delta \bigr ), \end{aligned}$$

with $M_{T, {{\textbf {A}}}}$ in (4.108) and $M_T^{(1)}$ in (4.120). From Proposition 4.17 we know that $-\frac{1}{2} M_{{T_{\text {c}}}}^{(1)} \Delta = \Psi \alpha _*$, which allows us to write $\alpha _{\Delta }$ as in (3.6). The operator $M_{T, {{\textbf {A}}}_{e_3}}$ equals $M_{T, {{\textbf {A}}}}$ in (4.108) with ${{\textbf {A}}}$ replaced by ${{\textbf {A}}}_{e_3}$. The contribution from this operator needs to be carefully isolated for the orthogonality property in (3.9) to hold. This should be compared to part (c) of [15, Proposition 3.2]. In the following, we will establish the properties of $\eta _0$ and $\eta _\perp$ that are stated in Proposition 3.2.

We will first prove (3.7), and start by noting that

$$\begin{aligned} {\mathcal {R}}_{T, {{\textbf {A}}}, W}^{(4)}(\Delta )&= \frac{1}{\beta }\sum _{n\in {\mathbb {Z}}} \frac{1}{\text {i}\omega _n - {\mathfrak {h}}_{{{\textbf {A}}}, W}} \, \Delta \, \frac{1}{\text {i}\omega _n + \overline{{\mathfrak {h}}_{{{\textbf {A}}}, W}}}\, \overline{\Delta }\, \Bigl [ \frac{1}{\text {i}\omega _n - H_\Delta }\Bigr ]_{11}\, \Delta \, \frac{1}{\text {i}\omega _n + \overline{{\mathfrak {h}}_{{{\textbf {A}}}, W}}}. \end{aligned}$$

An application of Hölder’s inequality shows $\Vert {\mathcal {R}}_{T, {{\textbf {A}}}, W}^{(4)}(\Delta )\Vert _2 \leqslant C \beta ^{3} \Vert \Delta \Vert _6^3$. With the operator $\pi = -\text {i}\nabla + {{\textbf {A}}}_{{{\textbf {B}}}}$ understood to act on the *x*-coordinate of the integral kernel of ${\mathcal {R}}_{T, {{\textbf {A}}}, W}^{(4)}(\Delta )$ we also have

$$\begin{aligned} \Vert \pi {\mathcal {R}}_{T, {{\textbf {A}}}, W}^{(4)}(\Delta ) \Vert _2 \leqslant \frac{1}{\beta }\sum _{n\in {\mathbb {Z}}} \Bigl \Vert \pi \frac{1}{\text {i}\omega _n - {\mathfrak {h}}_{{{\textbf {A}}}, W}} \Bigr \Vert _{\infty } \Bigl \Vert \frac{1}{\text {i}\omega _n + \overline{{\mathfrak {h}}_{{{\textbf {A}}}, W}}} \Bigr \Vert _{\infty }^2 \Bigl \Vert \Bigl [ \frac{1}{\text {i}\omega _n - H_\Delta }\Bigr ]_{11} \Bigr \Vert _{\infty } \Vert \Delta \Vert _6^3. \end{aligned}$$

An application of Cauchy–Schwarz shows

4.196 $$\begin{aligned} (- \text {i}\nabla + {{\textbf {A}}}_{{{\textbf {B}}}} + A)^2 + W_h \geqslant \frac{1}{2} (- \text {i}\nabla + {{\textbf {A}}}_{{{\textbf {B}}}} )^2 - C h^2. \end{aligned}$$

Accordingly, we haveIt follows that

4.197 $$\begin{aligned} \Vert \pi {\mathcal {R}}_{T, {{\textbf {A}}}, W}^{(4)}(\Delta ) \Vert _2 \leqslant C\, \Vert \Delta \Vert _6^3. \end{aligned}$$

The same argument with obvious adjustments also shows that $\Vert {\mathcal {R}}_{T, {{\textbf {A}}}, W}^{(4)}(\Delta )\pi \Vert _2$ is bounded by the right side of (4.197), too. Finally, (2.13), an application of Lemma 4.1, and (2.9) allow us to conclude that

4.198 $$\begin{aligned} \Vert {\mathcal {R}}_{T, {{\textbf {A}}}, W}^{(4)}(\Delta ) \Vert _{{H^1(Q_h \times {\mathbb {R}}_{{\text {s}}}^3)}}^2 \leqslant C \; h^6 \; \Vert \Psi \Vert _{H_{\text {mag}}^1(Q_h)}^6 \end{aligned}$$

holds.

To control $M_{T, {{\textbf {A}}}}\Delta - M_{T, {{\textbf {A}}}_{e_3}}\Delta$, we need the following proposition.

#### Proposition 4.33

Let $| \cdot | V\alpha _*\in L^{2}({\mathbb {R}}^3)$ for $k\in \{0,1\}$, let $A\in W^{1,\infty }({\mathbb {R}}^3,{\mathbb {R}}^3)$ be periodic, assume $\Psi \in H_{\text {mag}}^1(Q_h)$, and denote $\Delta \equiv \Delta _\Psi$ as in (3.1). For any $T_0>0$ there is $h_0>0$ such that for any $T\geqslant T_0$ and any $0 < h \leqslant h_0$ we have

4.199 $$\begin{aligned} \Vert M_{T, {{\textbf {A}}}} \Delta - M_{T, {{\textbf {A}}}_{e_3}} \Delta \Vert _{H^1(Q_h \times {\mathbb {R}}_{{\text {s}}}^3)}^2&\leqslant C \, h^5 \, \max _{k=0,1} \Vert \ | \cdot |^k \ V\alpha _*\Vert _2^2 \, \Vert \Psi \Vert _{H_{\text {mag}}^1(Q_h)}^2. \end{aligned}$$

#### Proof

Let us define the operator

$$\begin{aligned} {\mathcal {Q}}_{T, {{\textbf {B}}}, A}\alpha (X, r) {:}{=}\iint _{{\mathbb {R}}^3\times {\mathbb {R}}^3} \text {d}Z \text {d}s \; k_T(Z, r-s) \; \text {e}^{2\text {i}A_h(X) \cdot Z} \; (\text {e}^{\text {i}Z\cdot \Pi } \alpha )(X,s), \end{aligned}$$

where $k_T(Z,r) {:}{=}k_{T, 0}(0,Z, r, 0)$ with $k_{T, 0}$ in (4.45) and $\Pi = -\text {i}\nabla + 2 {{\textbf {A}}}_{{{\textbf {B}}}}$ is understood to act on the center-of-mass coordinate *X* of $\alpha$. We start our analysis by writing

4.200 $$\begin{aligned} M_{T, {{\textbf {A}}}} \Delta - M_{T, {{\textbf {A}}}_{e_3}} \Delta = \bigl ( M_{T, {{\textbf {A}}}} \Delta - {\mathcal {Q}}_{T, {{\textbf {B}}}, A} \Delta \bigr ) + \bigl ( Q_{T, {{\textbf {B}}}, A} \Delta - M_{T, {{\textbf {A}}}_{e_3}} \Delta \bigr ). \end{aligned}$$

In the following we derive bounds on the ${H^1(Q_h \times {\mathbb {R}}_{{\text {s}}}^3)}$-norms of the two terms on the right side of (4.199). When we use that the integrand in the definition of $M_{T, {{\textbf {A}}}}$ is symmetric with respect to the transformation $Z \mapsto -Z$ and apply Lemma 4.13, we see that

$$\begin{aligned}&(M_{T, {{\textbf {A}}}} \alpha - {\mathcal {Q}}_{T, {{\textbf {B}}}, A} \alpha )(X, r) \\&\quad = \iint _{{\mathbb {R}}^3\times {\mathbb {R}}^3} \text {d}Z \text {d}s\; k_T(Z, r-s) \, \bigl [ \text {e}^{\text {i}\Phi _{2A_h}(X, X+Z)} - \text {e}^{2\text {i}A_h(X) \cdot Z}\bigr ] \, (\text {e}^{\text {i}Z\cdot \Pi } \alpha )(X, s) \end{aligned}$$

holds. Let us also recall that $\Phi _{{{\textbf {A}}}}$ is defined in (4.2).

We have

$$\begin{aligned} \Phi _{2A}(X, X+ Z) - 2A(X)\cdot Z = 2\int _0^1 \text {d}t \; \bigl [ A(X + (1-t) Z) - A(X)\bigr ] \cdot Z, \end{aligned}$$

and hence

4.201 $$\begin{aligned} \bigl | \text {e}^{\text {i}\Phi _{2A}(X, X+Z)} - \text {e}^{2\text {i}A(X)\cdot Z} \bigr | \leqslant \Vert DA\Vert _\infty \; |Z|^2. \end{aligned}$$

When we apply this bound and $|Z|^2 \leqslant | Z + \frac{r}{2}|^2 + | Z - \frac{r}{2}|^2$, it follows that

$$\begin{aligned} \Vert M_{T, {{\textbf {A}}}}\Delta - {\mathcal {Q}}_{T, {{\textbf {B}}}, A}\Delta \Vert _2^2&\leqslant C\, \Vert \Psi \Vert _2^2 \, \Vert DA_h \Vert _\infty ^2 \, \Vert F_T^{2} \Vert _1 \, \Vert V\alpha _*\Vert _2^2 \end{aligned}$$

with $F_T^{2}$ in (4.93). Using the $L^1$-norm bound for $F_T^{2}$ in (4.95), we conclude the claimed estimate for this term.

To obtain a bound for the first gradient term, we start by noting that

$$\begin{aligned} \Vert \Pi (M_{T, A}\Delta - {\mathcal {Q}}_{T, {{\textbf {B}}}, A}\Delta )\Vert _2^2&\leqslant C \, \Vert \Psi \Vert _2^2 \int _{{\mathbb {R}}^3} \text {d}r \; \Bigl | \iint _{{\mathbb {R}}^3\times {\mathbb {R}}^3} \text {d}Z \text {d}s\; |k_T(Z, r-s)| \, |V\alpha _*(s)| \\&\quad \times \sup _{X\in {\mathbb {R}}^3} \bigl | \nabla _X \text {e}^{\text {i}\Phi _{2A_h}(X, X + Z)} - \nabla _X \text {e}^{2\text {i}A_h(X)\cdot Z} \bigr | \Bigr |^2 \\&\quad + C \, \Vert \Pi \text {e}^{\text {i}Z\cdot \Pi } \Psi \Vert _2 \, \int _{{\mathbb {R}}^3} \text {d}r \; \Bigl | \iint _{{\mathbb {R}}^3\times {\mathbb {R}}^3} \text {d}Z \text {d}s\; |k_T(Z, r-s)| \, |V\alpha _*(s)| \\&\quad \times \sup _{X\in {\mathbb {R}}^3} \bigl | \text {e}^{\text {i}\Phi _{2A_h}(X, X + Z)} - \text {e}^{2\text {i}A_h(X)\cdot Z}\bigr | \Bigr |^2, \end{aligned}$$

where $\Pi = -\text {i}\nabla + 2 {{\textbf {A}}}_{{{\textbf {B}}}}$ is understood to act on the center-of-mass coordinate. When we additionally use

$$\begin{aligned} \bigl | \nabla _X \text {e}^{\text {i}\Phi _{2A}(X, X + Z)} - \nabla _X\text {e}^{2\text {i}A(X)\cdot Z} \bigr |&\leqslant \bigl | \nabla _X \Phi _{2A}(X, X + Z) - 2\nabla _X A(X)\cdot Z \bigr | \\&+ \bigl |\Phi _{2A}(X, X+Z) - 2A(X)\cdot Z\bigr | \, |\nabla _XA(X)\cdot Z| \\&\leqslant \bigl [ \Vert D^2A\Vert _\infty + \Vert DA\Vert _\infty ^2 \bigr ] \bigl [ |Z|^2 + |Z|^3\bigr ], \end{aligned}$$

as well as

4.202 $$\begin{aligned} |Z|^a \leqslant \left| Z + \frac{r}{2} \right| ^a + \left| Z - \frac{r}{2} \right| ^a, \quad a \geqslant 0 \end{aligned}$$

for the choices $a=2,3$, $\Vert D^2A_h\Vert _\infty \leqslant Ch^3$, $\Vert DA_h\Vert _\infty ^2 \leqslant Ch^4$, (4.117), and (4.201), we see that

$$\begin{aligned} \Vert \Pi (M_{T, {{\textbf {A}}}} \Delta - {\mathcal {Q}}_{T, {{\textbf {B}}}, A} \Delta )\Vert _2^2&\leqslant C \, h^5 \, \Vert \Psi \Vert _{H_{\text {mag}}^1(Q_h)}^2 \, \Vert V\alpha _*\Vert _2^2 \, \left( \Vert F_T^{2} \Vert _1^2 + \Vert F_T^{3} \Vert _1^2 \right) \end{aligned}$$

holds with $F_T^{a}$ in (4.95). An application of (4.95) proves the claimed bound for this term.

In the last step we consider

$$\begin{aligned}&\Vert {\widetilde{\pi }} (M_{T, {{\textbf {A}}}} \Delta - {\mathcal {Q}}_{T, {{\textbf {B}}}, A}\Delta ) \Vert _2^2 \leqslant C \, \Vert \Psi \Vert _2^2 \\&\quad \times \int _{{\mathbb {R}}^3} \text {d}r \, \Bigl | \iint _{{\mathbb {R}}^3\times {\mathbb {R}}^3} \text {d}Z\text {d}s\; |{\widetilde{\pi }} k_T(Z, r-s)| \, |V\alpha _*(s)| \, \sup _{X\in {\mathbb {R}}^3} \bigl | \text {e}^{\text {i}\Phi _{2A_h}(X, X + Z)} - \text {e}^{2\text {i}A_h(X)\cdot Z}\bigr |\Bigr |^2. \end{aligned}$$

The estimate in (4.202) and $\frac{1}{4} |{{\textbf {B}}}\wedge r| \leqslant \frac{1}{4} |{{\textbf {B}}}| ( |r-s| + |s| )$ allow us to prove the bound

4.203 $$\begin{aligned} \int _{{\mathbb {R}}^3} \text {d}Z \; |{\widetilde{\pi }} k_T(Z, r-s)| \, |Z|^2 \leqslant F_T^3(r-s) \, (1 + |s| ) + G_T^2(r-s) \end{aligned}$$

with $F_T^a$ in (4.93) and $G_T^a$ in (4.94). In combination with (4.201), this proves the claimed bound for this term. It also ends the proof of the claimed bound for the first term on the right side of (4.200). It remains to consider the second term.

A short computation that uses $k_T(-Z,r-s) = k_T(Z,r-s)$ and $\cos (x) - 1 = -2\sin ^2(\frac{x}{2})$ shows

$$\begin{aligned}&({\mathcal {Q}}_{T, {{\textbf {B}}}, A}\alpha - M_{T, {{\textbf {A}}}_{e_3}}\alpha )(X, r) \\&\quad = -2 \iint _{{\mathbb {R}}^3\times {\mathbb {R}}^3} \text {d}Z \text {d}s \; k_T(Z, r-s) \, \sin ^2(A_h(X)\cdot Z) (\cos (Z\cdot \Pi ) \alpha )(X, s) \\&\quad \qquad - \int _{{\mathbb {R}}^3\times {\mathbb {R}}^3} \text {d}Z \text {d}s \; k_T(Z, r-s) \, \sin (2A_h(X) \cdot Z) \, (\sin (Z\cdot \Pi ) \alpha )(X, s). \end{aligned}$$

From this, we check that

$$\begin{aligned} \Vert {\mathcal {Q}}_{T, {{\textbf {B}}}, A}\Delta - M_{T, {{\textbf {A}}}_{e_3}}\Delta \Vert _2^2&\leqslant C\, \bigl [ \Vert \Psi \Vert _2^2 \, \Vert A_h\Vert _\infty ^4 + \Vert \Pi \Psi \Vert _2^2 \Vert A_h\Vert _\infty ^2 \bigr ] \, \Vert F_T^{2} \Vert _1^2 \, \Vert V\alpha _*\Vert _2^2 \end{aligned}$$

holds with $F_T^{a}$ in (4.93). When we use (4.201) to obtain a bound for the $L^1$-norm of $F_T^{2}$, this proves the claimed bound for this term.

Next, we note that

4.204 $$\begin{aligned}&\Vert \Pi ( {\mathcal {Q}}_{T, {{\textbf {B}}}, A}\Delta - M_{T, {{\textbf {A}}}_{e_3}}\Delta )\Vert _2^2 \leqslant C \, \int _{{\mathbb {R}}^3} \text {d}r \, \Bigl | \iint _{{\mathbb {R}}^3\times {\mathbb {R}}^3} \text {d}Z\text {d}s \; |k_T(Z, r-s)| \, |V\alpha _*(s)| \nonumber \\&\quad \times \Bigl [ \sup _{X \in {\mathbb {R}}^3} | \nabla _X \sin ^2 (A_h(X)\cdot Z) | \, \Vert \cos (Z\cdot \Pi )\Psi \Vert _2 + \sup _{X \in {\mathbb {R}}^3} | \sin ^2(A_h(X)\cdot Z) | \, \Vert \Pi \cos (Z\cdot \Pi )\Psi \Vert _2 \nonumber \\&\quad + \sup _{X \in {\mathbb {R}}^3} | \sin (2A_h(X)\cdot Z) | \, \Vert \sin (Z\cdot \Pi ) \Psi \Vert _2 + \sup _{X \in {\mathbb {R}}^3} | \sin (2A_h(X)\cdot Z) | \, \Vert \Pi \sin (Z\cdot \Pi ) \Psi \Vert _2\Bigr ]\Bigr |^2. \end{aligned}$$

We have

$$\begin{aligned} \Vert \sin (Z\cdot \Pi )\Psi \Vert _2 \leqslant C \, h^2 \, \Vert \Psi \Vert _{H_{\text {mag}}^1(Q_h)} \, |Z|. \end{aligned}$$

Furthermore, from a straight forward computation or from [15, Lemma 5.12], we know that

$$\begin{aligned} \Pi _X \, \sin (Z\cdot \Pi _X) = \sin (Z\cdot \Pi _X) \;\Pi _X + 2\text {i}\, \cos (Z\cdot \Pi _X) \; {{\textbf {B}}}\wedge Z, \end{aligned}$$

and hence

$$\begin{aligned} \Vert \Pi \sin (Z\cdot \Pi )\Psi \Vert _2&\leqslant C \, h^2 \Vert \Psi \Vert _{H_{\text {mag}}^1(Q_h)} \, (1 + |Z|). \end{aligned}$$

To obtain the bound we used that $|{{\textbf {B}}}| = h^2$. For $\Pi \cos (Z\cdot \Pi )\Psi$ a similar estimate was obtained in (4.116). Putting these bounds together, we find that the term on the left side of (4.204) is bounded by a constant times $h^6 \Vert V \alpha _* \Vert _2^2 \Vert \Psi \Vert _{H_{\text {mag}}^1(Q_h)}^2$. It remains to consider the term proportional to ${\widetilde{\pi }}$.

A straightforward computation shows that

$$\begin{aligned} \Vert {\widetilde{\pi }}({\mathcal {Q}}_{T, {{\textbf {B}}}, A}\Delta - M_{T, {{\textbf {A}}}_{e_3}}\Delta )\Vert _2^2&\leqslant C\bigl [ \Vert A_h\Vert _\infty ^4 \Vert \Psi \Vert _2^2 + \Vert A_h\Vert _\infty ^2 \Vert \Pi \Psi \Vert _2^2 \bigr ] \\&\quad \times \int _{{\mathbb {R}}^3} \text {d}r \, \Bigl | \iint _{{\mathbb {R}}^3\times {\mathbb {R}}^3} \text {d}Z \text {d}s \; |{\widetilde{\pi }} k_T(Z, r-s)| \, |V\alpha _*(s)| \, |Z|^2\Bigr |^2. \end{aligned}$$

We use (4.203) to see that the term on the left side is bounded by a constant times $h^6 \max _{k=0,1} \Vert \ | \cdot |^k \ V\alpha _*\Vert _2^2 \, \Vert \Psi \Vert _{H_{\text {mag}}^1(Q_h)}^2$. This proves Proposition 4.33. $\square$

The next lemma provides us with a bound for the term in (4.195) that is proportional to $L_{T, {{\textbf {A}}}, W} - L_{T, {{\textbf {A}}}}$.

#### Lemma 4.34

Let $V\alpha _*\in L^{2}({\mathbb {R}}^3)$, let $W \in L^{\infty }({\mathbb {R}}^3)$ and $A\in L^{\infty }({\mathbb {R}}^3,{\mathbb {R}}^3)$ be periodic, assume $\Psi \in H_{\text {mag}}^1(Q_h)$, and denote $\Delta \equiv \Delta _\Psi$ as in (3.1). For any $T_0>0$ there is $h_0>0$ such that for any $T\geqslant T_0$ and any $0 < h \leqslant h_0$ we have

$$\begin{aligned} \Vert L_{T, {{\textbf {A}}}, W} \Delta - L_{T, {{\textbf {A}}}} \Delta \Vert _{{H^1(Q_h \times {\mathbb {R}}_{{\text {s}}}^3)}}^2&\leqslant C \, h^6 \, \Vert V \alpha _* \Vert _2^2 \, \Vert \Psi \Vert _{H_{\text {mag}}^1(Q_h)}^2. \end{aligned}$$

#### Proof

To prove the lemma, we write $L_{T, {{\textbf {A}}}, W} \Delta - L_{T, {{\textbf {A}}}} \Delta$ as in (4.161) and use the representation of the $H^1$-norm in (2.13). The details are a straightforward application of arguments that have been used already several times above, and are therefore left to the reader. $\square$

When we combine Propositions 4.9, 4.11, 4.15, 4.17 as well as (4.198) and Lemma 4.34, we obtain the claimed bound for $\eta _0(\Delta )$ in (3.7), that is, part (a) of Proposition 3.2. The proofs of parts (b) and (c) can be found in [15], see the proofs of Proposition 3.2 (b), (c). This ends our proof of Proposition 3.2.

### Proof of Proposition 3.7

Let the assumptions of Proposition 3.7 hold. In the following we prove that there are constants $D_0>0$ and $h_0>0$ such that for $0 < h \leqslant h_0$ and

$$\begin{aligned} 0< T_0 \leqslant T < {T_{\text {c}}}(1 - D_0 h^2) \end{aligned}$$

there is a function $\Psi \in H_{\text {mag}}^2(Q_h)$ such that the energy of the Gibbs state $\Gamma _\Delta$ in (3.4) with gap function $\Delta (X,r) = -2 V\alpha _*(r) \Psi (X)$ satisfies (3.24).

Let $\psi \in H_{\text {mag}}^2(Q)$ with $\Vert \psi \Vert _{H_{\text {mag}}^2(Q_h)}=1$ and define $\Psi (X) = h \psi (hX)$. The function $\Psi$ satisfies $\Vert \Psi \Vert _{H_{\text {mag}}^2(Q_h)}=1$. When we apply Propositions 3.2, 3.5, 4.26, as well as (2.9) and (4.193), we find

$$\begin{aligned} {\mathcal {F}}^{\text {BCS}}_{h, T}(\Gamma _\Delta ) - {\mathcal {F}}^{\text {BCS}}_{h, T}(\Gamma _0)&< h^2 \, \bigl ( - cD_0 \, \Vert \psi \Vert _2^2 + C \bigr ) \end{aligned}$$

for *h* small enough. The proof of Proposition 3.7 is completed when we choose $D_0 = \frac{C}{c \Vert \psi \Vert _2^2}$.

## The structure of low-energy states

In this section we prove a priori bounds for low-energy states of the BCS functional in the sense of (5.1) below. The goal is to show that their Cooper pair wave function has a structure similar to that of the trial state we use in the proof of the upper bound in Sect. 3. These bounds and the trial state analysis in Sect. 3 are the main technical ingredients for the proof of the lower bound in Sect. 6. To prove the a priori bounds, we show that the periodic external potentials $W_h$ and $A_h$ can be treated as a perturbation, which reduces the problem to proving a priori bounds for the case of a constant magnetic field. The solution of this problem has been the main novelty in [15, Theorem 5.1] and we apply it here. In case of a magnetic field with zero flux through the unit cell such bounds have been proved for the first time in [23]. The idea to reduce the problem to the case of a constant magnetic field is inspired by a similar perturbative analysis in [23].

We recall the definition of the generalized one-particle density matrix $\Gamma$ in (1.5), its Cooper pair wave function $\alpha = \Gamma _{12}$, as well as the normal state $\Gamma _0$ in (1.12).

### Theorem 5.1

(Structure of low-energy states) Let Assumptions 1.1 and 1.3 hold. For given $D_0, D_1 \geqslant 0$, there is a constant $h_0>0$ such that for all $0 <h \leqslant h_0$ the following holds: If $T>0$ obeys $T - {T_{\text {c}}}\geqslant -D_0h^2$ and if $\Gamma$ is a gauge-periodic state with low energy, that is,

5.1 $$\begin{aligned} {\mathcal {F}}^{\text {BCS}}_{h, T}(\Gamma ) - {\mathcal {F}}^{\text {BCS}}_{h, T}(\Gamma _0) \leqslant D_1h^4, \end{aligned}$$

then there are $\Psi \in H_{\text {mag}}^1(Q_h)$ and $\xi \in {H^1(Q_h \times {\mathbb {R}}_{{\text {s}}}^3)}$ such that

5.2 $$\begin{aligned} \alpha (X,r) = \alpha _*(r) \Psi (X) + \xi (X,r), \end{aligned}$$

where

5.3 $$\begin{aligned} \sup _{0< h\leqslant h_0} \Vert \Psi \Vert _{H_{\text {mag}}^1(Q_h)}^2&\leqslant C,&\Vert \xi \Vert _{H^1(Q_h \times {\mathbb {R}}_{{\text {s}}}^3)}^2&\leqslant Ch^4 \bigl ( \Vert \Psi \Vert _{H_{\text {mag}}^1(Q_h)}^2 + D_1\bigr ). \end{aligned}$$

### Remarks

- Equation (5.3) shows that $\Psi$ is a macroscopic quantity in the sense that its $H_{\text {mag}}^1(Q_h)$-norm scales as that of the function in (1.20). It is important to note that the $H_{\text {mag}}^1(Q_h)$-norm is scaled with *h*, see (2.7). The unscaled $L_{\text {mag}}^2(Q_h)$-norm of $\Psi$ is of the order *h*, and therefore much larger than that of $\xi$, see (5.3).
- Theorem 5.1 has been proven in [15, Theorem 5.1] for the case of a constant external magnetic field, where $A_h =0$ and $W_h = 0$. Our proof of Theorem 5.1 for general external fields reduces the problem to that case.

Although Theorem 5.1 contains the natural a priori bounds for low-energy states, we need a slightly different version of it in our proof of the lower bound for the BCS free energy in Sect. 6. The main reason is that we intend to use the function $\Psi$ from the decomposition of the Cooper pair wave function of a low-energy state in (5.2) to construct a Gibbs state $\Gamma _{\Delta }$ as in (3.4). In order to be able to justify the relevant computations with this state, we need $\Psi \in H_{\text {mag}}^2(Q_h)$, which is not guaranteed by Theorem 5.1 above, see also Remark 3.3. The following corollary provides us with a decomposition of $\alpha$, where the center-of-mass wave function $\Psi _\leqslant$ has the required $H_{\text {mag}}^2(Q_h)$-regularity. A decomposition with a cut-off function of the form in the corollary has also been used in [15, 23, 24, 26].

### Corollary 5.2

Let the assumptions of Theorem 5.1 hold and let $\varepsilon \in [h^2, h_0^2]$. Let $\Psi$ be as in (5.2) and define

5.4 $$\begin{aligned} \Psi _\leqslant&{:}{=}\mathbb {1}_{[0,\varepsilon ]}(\Pi ^2) \Psi ,&\Psi _>&{:}{=}\mathbb {1}_{(\varepsilon ,\infty )}(\Pi ^2) \Psi . \end{aligned}$$

Then, we have

5.5 $$\begin{aligned} \Vert \Psi _\leqslant \Vert _{H_{\text {mag}}^1(Q_h)}^2&\leqslant \Vert \Psi \Vert _{H_{\text {mag}}^1(Q_h)}^2, \nonumber \\ \Vert \Psi _\leqslant \Vert _{H_{\text {mag}}^k(Q_h)}^2&\leqslant C\, (\varepsilon h^{-2})^{k-1} \, \Vert \Psi \Vert _{H_{\text {mag}}^1(Q_h)}^2, \qquad k\geqslant 2, \end{aligned}$$

as well as

5.6 $$\begin{aligned} \Vert \Psi _>\Vert _2^2&\leqslant C \varepsilon ^{-1}h^4 \, \Vert \Psi \Vert _{H_{\text {mag}}^1(Q_h)}^2,&\Vert \Pi \Psi _>\Vert _2^2&\leqslant Ch^4 \, \Vert \Psi \Vert _{H_{\text {mag}}^1(Q_h)}^2. \end{aligned}$$

Furthermore,

5.7 $$\begin{aligned} \sigma _0(X,r) {:}{=}\, \alpha _*(r) \Psi _>(X) \end{aligned}$$

satisfies

5.8 $$\begin{aligned} \Vert \sigma _0\Vert _{H^1_\text {symm}(Q_h\times {\mathbb {R}}^3)}^2&\leqslant C\varepsilon ^{-1}h^4 \, \Vert \Psi \Vert _{H_{\text {mag}}^1(Q_h)}^2 \end{aligned}$$

and, with $\xi$ in (5.2), the function

5.9 $$\begin{aligned} \sigma {:}{=}\, \xi + \sigma _0 \end{aligned}$$

obeys

5.10 $$\begin{aligned} \Vert \sigma \Vert _{H^1_\text {symm}(Q_h\times {\mathbb {R}}^3)}^2 \leqslant Ch^4 \bigl ( \varepsilon ^{-1}\Vert \Psi \Vert _{H_{\text {mag}}^1(Q_h)}^2 + D_1\bigr ). \end{aligned}$$

In terms of these functions, the Cooper pair wave function $\alpha$ of the low-energy state $\Gamma$ in (5.1) admits the decomposition

5.11 $$\begin{aligned} \alpha (X,r) = \alpha _*(r) \Psi _\leqslant (X) + \sigma (X,r). \end{aligned}$$

For a proof of the corollary we refer to the proof of Corollary 5.2 in [15].

### A lower bound for the BCS functional

We start the proof of Theorem 5.1 with the following lower bound on the BCS functional, whose proof is literally the same as that of the comparable statement in [23].

#### Lemma 5.3

Let $\Gamma _0$ be the normal state in (1.12). We have the lower bound

5.12 $$\begin{aligned} {\mathcal {F}}^{\text {BCS}}_{h, T}(\Gamma ) - {\mathcal {F}}^{\text {BCS}}_{h, T}(\Gamma _0) \geqslant {{\,\textrm{Tr}\,}}\bigl [ (K_{T,{{\textbf {A}}}, W} - V) \alpha \alpha ^*\bigr ] + \frac{4T}{5} {{\,\textrm{Tr}\,}}\bigl [ (\alpha ^* \alpha )^2\bigr ], \end{aligned}$$

where

5.13 $$\begin{aligned} K_{T, {{\textbf {A}}}, W} = \frac{(-\text {i}\nabla + {{\textbf {A}}}_h)^2 + W_h- \mu }{\tanh (\frac{(-\text {i}\nabla + {{\textbf {A}}}_h)^2 + W_h - \mu }{2T})} \end{aligned}$$

and $$V\alpha (x,y) = V(x-y) \alpha (x,y)$$.

In Proposition A.1 in “Appendix A” we show that the external electric and magnetic fields can lower the lowest eigenvalue zero of $K_{{T_{\text {c}}}} - V$ at most by a constant times $h^2$. We use this in the next lemma to show that $K_{T, {{\textbf {A}}}, W} - V$ is bounded from below by a nonnegative operator, up to a correction of the size $Ch^2$.

#### Lemma 5.4

Let Assumptions 1.1 and 1.3 be true. For any $D_0 \geqslant 0$, there are constants $h_0>0$ and $T_0>0$ such that for $0< h\leqslant h_0$ and $T>0$ with $T - {T_{\text {c}}}\geqslant -D_0h^2$, the estimate

5.14 $$\begin{aligned} K_{T, {{\textbf {A}}}, W} - V&\geqslant c \; (1 - P) (1 + \pi ^2) (1- P) + c \, \min \{ T_0, (T - {T_{\text {c}}})_+\} - Ch^2 \end{aligned}$$

holds. Here, $P = |\alpha _*\rangle \langle \alpha _*|$ is the orthogonal projection onto the ground state $\alpha _*$ of $K_{{T_{\text {c}}}} - V$ and $\pi = -\text {i}\nabla + {{\textbf {A}}}_{{\textbf {B}}}$.

#### Proof

Since $W \in L^{\infty }({\mathbb {R}}^3)$ we can use Lemma A.7 to show that $K_{T, {{\textbf {A}}}, W} \geqslant K_{T, {{\textbf {A}}}, 0} - C h^2$ holds. The rest of the proof goes along the same lines as that of [15, Lemma 5.4] with the obvious replacements. In particular, [15, Proposition A.1] needs to be replaced by Proposition A.1. We omit the details. $\square$

We deduce two corollaries from (5.12) and Lemma 5.4. The first statement is an a priori bound that will be used in the proof of Theorem 2 (b). Its proof goes along the same lines as that of [15, Corollary 5.5].

#### Corollary 5.5

Let Assumptions 1.1 and 1.3 be true. Then, there are constants $h_0>0$ and $C>0$ such that for all $0 < h \leqslant h_0$ and all temperatures $T\geqslant {T_{\text {c}}}(1 + Ch^2)$, we have ${\mathcal {F}}^{\text {BCS}}_{h, T}(\Gamma ) - {\mathcal {F}}^{\text {BCS}}_{h, T}(\Gamma _0) >0$ unless $\Gamma = \Gamma _0$.

The second corollary provides us with an inequality for Cooper pair wave functions of low-energy BCS states in the sense of (5.1). The left side of (5.16) appears as a lower bound for the full BCS functional. Despite of its apparent simplicity, it still contains all the information needed for a proof of Theorem 5.1. Before we state the corollary, let us define the operator

5.15 $$\begin{aligned} U&{:}{=}\text {e}^{-\text {i}\frac{r}{2} \Pi _X}, \end{aligned}$$

with $\Pi _X$ in (2.4), which acts on the relative coordinate $r = x-y$ as well as on the center-of-mass coordinate $X = \frac{x+y}{2}$ of a function $\alpha (X,r)$.

#### Corollary 5.6

Let Assumptions 1.1 and 1.3 be true. For any $D_0, D_1 \geqslant 0$, there is a constant $h_0>0$ such that if $\Gamma$ is a low-energy state in the sense that it satisfies (5.1), if $0 < h\leqslant h_0$, and if *T* is such that $T - {T_{\text {c}}}\geqslant -D_0h^2$, then $\alpha = \Gamma _{12}$ obeys

5.16 $$\begin{aligned} \langle \alpha , [U(1 - P)(1 + \pi ^2)(1 - P)U^* + U^*(1 - P)(1 + \pi ^2)(1&- P)U] \alpha \rangle + {{\,\textrm{Tr}\,}}\bigl [(\alpha ^* \alpha )^2\bigr ] \nonumber \\&\quad \leqslant C h^2 \Vert \alpha \Vert _2^2 {+} D_1h^4, \end{aligned}$$

where $P = | \alpha _* \rangle \langle \alpha _* |$ and $\pi = -\text {i}\nabla _r + {{\textbf {A}}}_{{\textbf {B}}}(r)$ both act on the relative coordinate *r* of $\alpha (X,r)$.

In the statement of the corollary and in the following, we refrain from equipping the operator $\pi$ and the projection $P = |\alpha _*\rangle \langle \alpha _*|$ with an index *r* although it acts on the relative coordinate. This does not lead to confusion and keeps the formulas readable. The proof of the corollary is inspired by the proof of [26, Proposition 23].

#### Proof of Corollary 5.6

In the following we use the notation $\pi _{{{\textbf {A}}}}^x = -\text {i}\nabla _x + {{\textbf {A}}}(x)$. We claim that

5.17 $$\begin{aligned} \pi _{{{\textbf {A}}}_h}^x&= U \pi _{{{\textbf {A}}}^+_h}^r U^*,&-\pi _{{{\textbf {A}}}_h}^y&= U^* \pi _{{{\textbf {A}}}^-_h}^r U, \end{aligned}$$

where $$\pi _{{{\textbf {A}}}^\pm }^r = -\text {i} \nabla _r + {{\textbf {A}}}^{\pm }(r)$$ with

$$\begin{aligned} {{\textbf {A}}}^\pm (r)&{:}{=}{{\textbf {A}}}_{e_3}(r) \pm A(X \pm r). \end{aligned}$$

To obtain (5.17), we use the identity $\text {e}^{\text {i}{{\textbf {B}}}\cdot (X\wedge Z)}\text {e}^{\text {i}Z\cdot P_X} = \text {e}^{\text {i}Z\cdot \Pi _X}$ in (4.90) to see that

$$\begin{aligned} U \; (-\text {i} \nabla _r + {{\textbf {A}}}_{{{\textbf {B}}}}(r)) \, U^*&= -\text {i} \nabla _r + \frac{1}{2} {{\textbf {A}}}_{{{\textbf {B}}}}(r) + \frac{1}{2} \Pi _X = \pi _{{{\textbf {A}}}_{{\textbf {B}}}}^x, \\ U^* (-\text {i} \nabla _r + {{\textbf {A}}}_{{{\textbf {B}}}}(r)) \, U \;&= -\text {i} \nabla _r + \frac{1}{2} {{\textbf {A}}}_{{{\textbf {B}}}}(r) - \frac{1}{2} \Pi _X = -\pi _{{{\textbf {A}}}_{{\textbf {B}}}}^y, \end{aligned}$$

where $\Pi _X = -\text {i}\nabla _X + 2 {{\textbf {A}}}_{{{\textbf {B}}}}(X)$. Equation (5.17) is a direct consequence of these two identities.

We also have

$$\begin{aligned} W(x)&= U \, W^+(r) \, U^*,&W(y)&= U^* \, W^-(r) \, U,&W^\pm (r)&{:}{=}W(X \pm r). \end{aligned}$$

Consequently, if $K_{T, {{\textbf {A}}}, W}^x$ and $K_{T, {{\textbf {A}}}, W}^y$ denote the operators $K_{T, {{\textbf {A}}}, W}$ acting on the *x* and *y* coordinate, respectively, we infer

5.18 $$\begin{aligned} K_{T, {{\textbf {A}}}, W}^x - V(r)&= U^* ( K_{T, {{\textbf {A}}}^+, W^+}^r - V(r) ) \, U, \nonumber \\ K_{T, {{\textbf {A}}}, W}^y - V(r)&= U \; ( K_{T, {{\textbf {A}}}^-, W^-}^r - V(r) ) \, U^*. \end{aligned}$$

We highlight that ${{\textbf {A}}}^\pm$ and $W^\pm$ depend on *X*.

The operator *V* in (5.12) acts by multiplication with the function $V(x-y)$. We use the symmetry $\alpha (x,y) = \alpha (y,x)$ to deduce5.19In combination, (5.1), (5.12), (5.18), and (5.19) therefore prove the bound

$$\begin{aligned} \frac{1}{2}\langle \alpha , [U^* ( K_{T, {{\textbf {A}}}^+, W^+}^r - V(r) ) \, U + U \; ( K_{T, {{\textbf {A}}}^-, W^-}^r - V(r) ) \, U^*]\alpha \rangle + c {{\,\textrm{Tr}\,}}\bigl [ (\alpha ^* \alpha )^2\bigr ] \leqslant D_1 h^4. \end{aligned}$$

An application of Lemma 5.4 on the left side establishes (5.16). $\square$

### Proof of Theorem 5.1

The statement in Theorem 5.1 is a direct consequence of Corollary 5.6 above and the results in [15]. More precisely, we need to combine Corollary 5.6, [15, Proposition 5.7], [15, Lemma 5.14], and the arguments in [15, Section 5.4].

## The lower bound on (1.22) and proof of Theorem 2 (b)

### The BCS energy of low-energy states

In this section, we complete the proofs of Theorems 1 and 2, which amounts to providing the lower bound on (1.22), the bound in (1.27), and the proof of Theorem 2 (b). Since these proofs mostly go along the same lines as those in [15], we only mention the differences and keep the presentation to a minimal length. Once the a priori estimates in [15, Theorem 5.1] are proved, the proofs of the lower bound for the free energy, the decomposition of the Cooper pair wave function of an approximate minimizer, and the upper bound for the critical temperature shift in [15] follow the same strategy as the related proofs in [23, 24]. In the following we will, however, only refer to [15] because our presentation is closer to the analysis in this reference than to those in [23, 24].

Let $D_1\geqslant 0$ and $D\in {\mathbb {R}}$ be given, choose $T = {T_{\text {c}}}(1 - Dh^2)$, and assume that $\Gamma$ is a gauge-periodic state that satisfies (5.1). Corollary 5.2 guarantees a decomposition of the Cooper pair wave function $\alpha = [\Gamma ]_{12}$ in terms of $\Psi _{\leqslant }$ in (5.4) and $\sigma$ in (5.9). The function $\Psi _{\leqslant }$ satisfies the bounds

6.1 $$\begin{aligned} \Vert \Psi _{\leqslant } \Vert _{H_{\text {mag}}^1(Q_h)}^2&\leqslant \Vert \Psi \Vert _{H_{\text {mag}}^1(Q_h)}^2 \leqslant C,&\Vert \Psi _{\leqslant } \Vert _{H_{\text {mag}}^2(Q_h)}^2&\leqslant C \varepsilon h^{-2} \Vert \Psi \Vert _{H_{\text {mag}}^1(Q_h)}^2, \end{aligned}$$

with $\Psi$ in (5.2). Let us define the state $\Gamma _{\Delta }$ as in (3.4) with $\Delta (X,r) = -2 V \alpha _* (r) \Psi _{\leqslant }(X)$. We apply Proposition 3.5 and Theorem 3.6 to obtain the following lower bound for the BCS energy of $\Gamma$:6.2In the next section we prove a lower bound for the terms in the second line of (6.2).

### Estimate on the relative entropy

The arguments in [15, Eqs. (6.1)-(6.14)] apply in literally the same way here, too. We obtain the correct bounds when we replace *B* by $h^2$ in all formulas. This, in particular, applies to the statement of [15, Lemma 6.2]. The only difference is that [15, Eq. (6.10)] is now given bywhich is due to the reason that the bound for the $L^2$-norm of $\eta _0$ in Proposition 3.2 is worse than the comparable bound we obtained in [15, Proposition 3.2]. This, however, does not change the size of the remainder in the final bound because other error terms come with a worse rate.

With the choice , we therefore obtain the bound6.3which is the equivalent of [15, Eq (6.14)].

### Conclusion

The arguments in [15, Section 6.3] apply in the same way also here and we obtain the correct formulas when we replace  by *h*. This concludes the proof of Theorem 1 and Theorem 2.

### Proof of the equivalent of [15, Lemma 6.2] in our setting

To obtain a proof of the equivalent of [15, Lemma 6.2] in our setting, we follow the strategy of proof in [15]. The additional terms coming from the external electric potential are not difficult to bound because *W* is a bounded function. To obtain bounds of the correct size in *h* for the terms involving the periodic vector potential $A_h$, we need to use that $A(0) = 0$, which is guaranteed by Assumption 1.1. This is relevant for example when we estimate our equivalent of the term on the left side of [15, Eq. (6.24)], that is,

$$\begin{aligned} \Vert [ \pi _{{{\textbf {A}}}_h}^2 + W_h(r) - p_r^2 ] \sigma _0 \Vert _2 \end{aligned}$$

with $p_r = - \text {i} \nabla _r$ and $\sigma _0$ in (5.7). We write the operator multiplying $\sigma _0$ as

6.4 $$\begin{aligned} \pi _r^2 - p_r^2 + W(r) + A(r) \cdot \pi _r + \pi _r \cdot A(r) + |A(r)|^2, \end{aligned}$$

where $\pi _r = -\text {i}\nabla _r + {{\textbf {A}}}_{{{\textbf {B}}}}(r)$. When we use (5.6), we see that the terms involving $|A_h|^2$ and $W_h$ are bounded by6.5Moreover, from [15, Eq. (6.24)] we know thatTo obtain a bound for the contribution from the fourth and the fifth term on the right side of (6.4), we write

$$\begin{aligned} A_h(r) = h^2 \int _0^1 \text {d}t \ (D A)(h r t) \cdot r, \end{aligned}$$

where *DA* denotes the Jacobi matrix of *A*. Hence,The term involving $\pi _r \cdot A_h(r)$ can be treated similarly when we commute $\pi _r$ to the right. In combination, the above considerations showAll other bounds in the proof of the equivalent of [15, Lemma 6.2] in our setting that involve $W_h$ or $A_h$ can be estimated with similar ideas. We therefore omit further details.

## Data Availability

Data sharing not applicable to this article as no datasets were generated or analysed during the current study.

## Gauge-invariant perturbation theory for $K_{{T_{\text {c}}}, {{\textbf {A}}}} -V$

In this appendix, we discuss the behavior of the eigenvalues below the essential spectrum of the operator $K_{{T_{\text {c}}}, {{\textbf {A}}}} - V$ for small $h >0$, where $K_{{T_{\text {c}}}, {{\textbf {A}}}} {:}{=}K_{{T_{\text {c}}}, {{\textbf {A}}}, 0}$ with $K_{T, {{\textbf {A}}}, 0}$ defined in (5.13). We recall that the full magnetic vector potential is given by ${{\textbf {A}}}{:}{=}{{\textbf {A}}}_{e_3} + A$ with the magnetic vector potential ${{\textbf {A}}}_{e_3}(x) = \frac{1}{2} e_3\wedge x$ of a constant magnetic field and a periodic vector potential *A* that satisfies $A(0) =0$. We also recall the notation ${{\textbf {A}}}_h (x) {:}{=}h{{\textbf {A}}}(hx)$. The goal of this appendix is to prove the following proposition.

### Proposition A.1

Assume $(1+|\cdot |^2) V \in L^{2}({\mathbb {R}}^3) \cap L^{\infty }({\mathbb {R}}^3)$ and let $A \in W^{3, \infty }({\mathbb {R}}^3; {\mathbb {R}}^3)$ be a periodic function. Then there is $h_0>0$ such that for $0 < h \leqslant h_0$ the following statements hold:

- Let $\lambda$ be an isolated eigenvalue of multiplicity $m \in {\mathbb {N}}$ of the operator $K_{T_{\text {c}}}- V$ with spectral projection *P*. Then there are exactly *m* eigenvalues $\lambda _1(h), \ldots ,\lambda _m(h)$ of the operator $K_{{T_{\text {c}}}, {{\textbf {A}}}}-V$ with spectral projection *P*(*h*) such that
  A.1 $$\begin{aligned} \max _{i=1,\ldots ,m} | \lambda _i(h) - \lambda |&\leqslant C h^2,&\Vert P(h) - P \Vert _{\infty }&\leqslant C h^2. \end{aligned}$$
- Assume that $K_{T_{\text {c}}}-V$ has a simple lowest eigenvalue with eigenfunction $\alpha _*$ and denote by $\alpha _*^h$ the eigenfunction to the lowest eigenvalue of $K_{{T_{\text {c}}}, {{\textbf {A}}}} -V$, which is normalized such that $\langle \alpha _*, \alpha _*^h \rangle \geqslant 0$ holds. Then we have the bound
  A.2 $$\begin{aligned} \Vert (1+\pi ^2) (\alpha _*^h - \alpha _*) \Vert _2 \leqslant C h^2, \end{aligned}$$
  where $\pi ^2 = (-\text {i}\nabla + {{\textbf {A}}}_{{{\textbf {B}}}})^2$ denotes the magnetic Laplacian.

### Remark A.2

The above Proposition should be compared to [15, Proposition A.1], where a similar statement is proved in the special case, where $V \geqslant 0$ and where ${{\textbf {A}}}(x) = \frac{1}{2} e_3 \wedge x$. In the reference the assumption for *V* is needed to assure that the Birman–Schwinger operator related to $K_{T,{{\textbf {A}}}} - V$ is self-adjoint. To prove the above proposition we investigate the resolvent kernel of $K_{T,{{\textbf {A}}}} - V$ rather than the Birman–Schwinger operator, which is the reason why we do not need the positivity assumption for *V*. The main technical ingredient of our proof is the estimate for the integral kernel of the resolvent of $K_{T} - V$ in Lemma A.4 below. Afterwards, we apply a standard version of gauge-invariant perturbation theory. As has been shown in [40, Chapter 6] one can also obtain exponential decay estimates for the resolvent kernel of $K_{T} - V$ via Combes–Thomas estimates. The proof, however, requires more effort than for the case of the Laplacian because $K_{T}$ has a more complicated structure. Since these estimates are not needed for the proof of Proposition A.1,we refrain from presenting them here.

We denote the resolvent of $K_{T, {{\textbf {A}}}} - V$ at $z\in \rho (K_{T, {{\textbf {A}}}} - V)$ by ${\mathcal {R}}_{{\textbf {A}}}^{z, V} {:}{=}(z - (K_{{T_{\text {c}}}, {{\textbf {A}}}} - V))^{-1}$. The integral kernel of ${\mathcal {R}}_{{\textbf {A}}}^{z, V}$ is denoted by ${\mathcal {G}}_{{\textbf {A}}}^{z, V}(x,y)$. We highlight that we use another symbol for the resolvent and for its integral kernel in this section. Since we frequently work with $L^p$-norms of integral kernels this simplifies our notation. If ${{\textbf {A}}}=0$, we write ${\mathcal {R}}^{z, V}$ for ${\mathcal {R}}_0^{z, V}$ and ${\mathcal {G}}^{z, V}$ for ${\mathcal {G}}_0^{z, V}$. Similarly, ${\mathcal {G}}^z$ stands for ${\mathcal {G}}^{z, 0}$. Before we give the proof of the above proposition, we state and prove four preparatory lemmas.

### Preparatory lemmas

The first lemma concerns the regularity of the kernel ${\mathcal {G}}^z$.

#### Lemma A.3

There is a continuous function $a :{\mathbb {C}} \backslash [2{T_{\text {c}}},\infty ) \rightarrow {\mathbb {R}}_+$ such that

A.3 $$\begin{aligned} \int _{{\mathbb {R}}^3} \text {d}x \ (1+x^2) | \nabla {\mathcal {G}}^z(x) | \leqslant a(z). \end{aligned}$$

#### Proof

We use the resolvent identity ${\mathcal {R}}^z = {\mathcal {R}}^0 + z {\mathcal {R}}^0 {\mathcal {R}}^z$ to write ${\mathcal {G}}^z$ as

A.4 $$\begin{aligned} {\mathcal {G}}^z(x) = {\mathcal {G}}^0(x) + z \int _{{\mathbb {R}}^3} \text {d}v \; {\mathcal {G}}^0(x-v) \, {\mathcal {G}}^z(v), \end{aligned}$$

which implies

A.5 $$\begin{aligned} \Vert (1+|\cdot |^2) \nabla {\mathcal {G}}^z \Vert _1 \leqslant \Vert (1+2 |\cdot |^2) \nabla {\mathcal {G}}^0 \Vert _1 \left( 1 + |z|\, \Vert (1+ |\cdot |^2) {\mathcal {G}}^z \Vert _1 \right) . \end{aligned}$$

The second $L^1({\mathbb {R}}^3)$-norm on the right side of (A.5) is bounded by

A.6

which, when multiplied with *z*, meets the requirements of the lemma. It therefore remains to consider $\Vert (1+|\cdot |^2) \nabla {\mathcal {G}}^0 \Vert _1$.

From [23, Eq. (A.6)] and [39, Theorem 6.23] we know that ${\mathcal {G}}^0(x)$ can be written as

A.7 $$\begin{aligned} {\mathcal {G}}^0(x)&= \frac{2}{\pi } \sum _{n=1}^{\infty } \frac{1}{n - \frac{1}{2}} \text {Im} \ g_0^{\text {i}(n-\frac{1}{2}) 2 \pi {T_{\text {c}}}}(x) \\&= \frac{1}{2 \pi ^2 |x|} \sum _{n=1}^{\infty } \frac{1}{n - \frac{1}{2}} \text {Im} \exp \Bigl ( \text {i}\sqrt{\mu +\text {i}\left( n-\frac{1}{2} \right) 2 \pi {T_{\text {c}}}} \, |x| \Bigr ), \nonumber \end{aligned}$$

where $g_0^z$ is the resolvent kernel of the Laplacian in (4.3) and $\sqrt{\cdot }$ denotes the principal square root. We use ${{\,\mathrm{\text {Im}}\,}}\text {e}^{\text {i}z} = \sin ({{\,\mathrm{\text {Re}}\,}}z) \exp (- {{\,\mathrm{\text {Im}}\,}}z)$ for $z \in {\mathbb {C}}$ and

A.8 $$\begin{aligned} \sqrt{a+ \text {i}b} = \frac{1}{\sqrt{2}} \sqrt{\sqrt{a^2+b^2}+a} + \frac{\text {i}{{\,\textrm{sgn}\,}}(b)}{\sqrt{2}} \sqrt{\sqrt{a^2+b^2}-a} \end{aligned}$$

for $a, b \in {\mathbb {R}}$ to see that

A.9 $$\begin{aligned} \text {Im} \exp \Bigl ( \text {i}\sqrt{\mu +\text {i}\left( n-\frac{1}{2} \right) 2 \pi {T_{\text {c}}}} \, |x| \Bigr ) = \sin \left( |x| c_n^{+} \right) \exp \left( -|x| c_n^{-} \right) , \end{aligned}$$

where

A.10 $$\begin{aligned} c_n^{\pm } = \frac{ 1 }{ \sqrt{2} } \sqrt{ \sqrt{\mu ^2 + ((n-1/2) 2 \pi {T_{\text {c}}})^2} \pm \mu }. \end{aligned}$$

In particular,

A.11 $$\begin{aligned} \nabla {\mathcal {G}}^0(x)&= -\frac{x}{2 \pi ^2 |x|^3} \sum _{n=1}^{\infty } \frac{1}{n - \frac{1}{2}} \sin \left( |x| c_n^{+} \right) \exp \left( -|x| c_n^{-} \right) \nonumber \\&\quad + \frac{x}{2 \pi ^2 |x|^2} \sum _{n=1}^{\infty } \frac{c_n^{+}}{n - \frac{1}{2}} \cos \left( |x| c_n^{+} \right) \exp \left( -|x| c_n^{-} \right) \nonumber \\&\quad - \frac{x}{2 \pi ^2 |x|^2} \sum _{n=1}^{\infty } \frac{c_n^{-}}{n - \frac{1}{2}} \sin \left( |x| c_n^{+} \right) \exp \left( -|x| c_n^{-} \right) . \end{aligned}$$

The above formula implies the bound

A.12 $$\begin{aligned} \Vert (1+|\cdot |^2) \nabla {\mathcal {G}}^0 \Vert _1&\leqslant \sum _{n=1}^{\infty } \frac{C}{n} \int _{0}^{\infty } \text {d}r \left( 1+r^2 + (r+r^3) \sqrt{1+n} \right) \exp \left( -r c_n^{-} \right) \nonumber \\&\leqslant C \sum _{n=1}^{\infty } n^{-\frac{3}{2}}. \end{aligned}$$

This proves the claim of the lemma. $\square$

The second lemma concerns bounds for the operator norm of $(z- (K_{T_{\text {c}}}- V))^{-1}$ and commutators of this operator with *x*, when viewed as maps from $L^2({\mathbb {R}}^3)$ to $L^{\infty }({\mathbb {R}}^3)$. Here and in the following we denote by $\Vert A \Vert _{2;\infty }$ the norm of a bounded operator *A* from $L^2({\mathbb {R}}^3) \rightarrow L^{\infty }({\mathbb {R}}^3)$. From [48, Corollary A.1.2] we know that if such an operator is also a bounded from $L^2({\mathbb {R}}^3)$ to $L^2({\mathbb {R}}^3)$ then it has an integral kernel given by a measurable function *A*(*x*, *y*), which obeys

A.13

Moreover, the norm $\Vert A \Vert _{2;\infty }$ equals the norm of the integral kernel of *A* in (A.13).

The following Lemma A.4 implies that the resolvent kernel ${\mathcal {G}}^{z, V}$ satisfies

A.14

In passing we note that our assumptions on *V* would allow for more: it can be shown that ${\mathcal {G}}^{z, V}$ is exponentially decaying in the sense that (A.14) holds with $1 + |x-y|^4$ replaced by $\text {e}^{\delta |x-y|}$ for some $\delta >0$ depending on the distance of *z* to the spectrum of $K_{T_{\text {c}}}- V$. For more information we refer to Remark A.2 above.

#### Lemma A.4

Assume that $V \in L^{\infty }({\mathbb {R}}^3)$ vanishes at infinity. Then there is a continuous function $a :\rho (K_{T_{\text {c}}}-V) \rightarrow {\mathbb {R}}_+$ such that

A.15 $$\begin{aligned} \Vert {\mathcal {R}}^{z, V} \Vert _{2;\infty } + \bigl \Vert [x, {\mathcal {R}}^{z, V} ] \bigr \Vert _{2;\infty } + \bigl \Vert [x,[x, {\mathcal {R}}^{z, V} ]] \bigr \Vert _{2;\infty } \leqslant a(z). \end{aligned}$$

#### Proof

We start by proving the bound for the first term on the right side of (A.15). We use the fact that $(1-\Delta )^{-1}$ is a bounded linear map from $L^2({\mathbb {R}}^3)$ to $L^{\infty }({\mathbb {R}}^3)$ and the resolvent identity to estimate

A.16 $$\begin{aligned} \Vert {\mathcal {R}}^{z, V} \Vert _{2;\infty } \leqslant C \Vert (1-\Delta ) {\mathcal {R}}^{z, V} \Vert _\infty \leqslant C \Vert (1-\Delta ) {\mathcal {R}}^z \Vert _\infty \left( 1 + \Vert V \Vert _\infty \Vert {\mathcal {R}}^{z, V} \Vert _\infty \right) . \end{aligned}$$

Our assumptions on *V* imply $\rho (K_{T_{\text {c}}}- V) \subseteq \rho (K_{T_{\text {c}}})$, whence both *z*-dependent terms on the right side meet the requirements of the lemma.

To obtain a bound for the second term on the right side of (A.15), we note that

A.17 $$\begin{aligned}{}[x, {\mathcal {R}}^{z, V} ]&= {\mathcal {R}}^{z, V} [K_{T_{\text {c}}},x] {\mathcal {R}}^{z, V},&[K_{T_{\text {c}}},x]&= -\text {i}(\nabla f)(-\text {i}\nabla ), \end{aligned}$$

where $f(p) {:}{=}K_{T_{\text {c}}}(p)$ is the symbol in (1.14). Using this, we estimate

A.18 $$\begin{aligned} \Vert [x,{\mathcal {R}}^{z, V}] \Vert _{2;\infty } \leqslant \Vert {\mathcal {R}}^{z, V} \Vert _{2;\infty } \, \Vert (\nabla f)(-\text {i}\nabla ) {\mathcal {R}}^{z, V} \Vert _\infty . \end{aligned}$$

A bound for the first factor on the right side was obtained in (A.16). Using the resolvent identity again, we bound the second factor by

A.19 $$\begin{aligned} \Vert (\nabla f)(-\text {i}\nabla ) {\mathcal {R}}^{z, V} \Vert _\infty \leqslant \Vert (\nabla f)(-\text {i}\nabla ) {\mathcal {R}}^z \Vert _\infty \left( 1 + \Vert V \Vert _\infty \Vert {\mathcal {R}}^{z, V} \Vert _\infty \right) , \end{aligned}$$

which proves the claim for the second term on the right side of (A.15). A bound for the third term can be derived similarly, and we therefore leave the remaining details to the reader. This proves the claim. $\square$

#### Lemma A.5

Assume $(1+|\cdot |^2)V \in L^{2}({\mathbb {R}}^{3}) \cap L^{\infty }({\mathbb {R}}^{3})$ and let $\alpha$ be an eigenfunction of the operator $K_{T_{\text {c}}}- V$ with eigenvalue $\lambda < 2 {T_{\text {c}}}$. Then we have

A.20 $$\begin{aligned} \Vert \, |\cdot | \nabla \alpha \Vert _2 + \Vert \, |\cdot |^2 \alpha \Vert _2 < \infty . \end{aligned}$$

#### Proof

We use the eigenvalue equation to write the Fourier transform of $\alpha$ as

A.21 $$\begin{aligned} {\widehat{\alpha }}(p) = -\frac{1}{\lambda - K_{T_{\text {c}}}(p)} \, ({\widehat{V}} *{\widehat{\alpha }})(p). \end{aligned}$$

Using Young’s inequality, we see that this implies

A.22

To prove the other bound, we use the resolvent identity to write (A.21) as

A.23 $$\begin{aligned} {\widehat{\alpha }}(p) = - \frac{1}{K_{T_{\text {c}}}(p)} \, ({\widehat{V}} *{\widehat{\alpha }})(p) + \frac{\lambda }{K_{T_{\text {c}}}(p)} \frac{1}{\lambda - K_{T_{\text {c}}}(p)} \, ({\widehat{V}} *{\widehat{\alpha }})(p). \end{aligned}$$

We argue as in (A.22) to see that the $L^2({\mathbb {R}}^3)$-norm of $\nabla _p p \frac{\lambda }{K_{T_{\text {c}}}(p)} \frac{1}{\lambda - K_{T_{\text {c}}}(p)} ({\widehat{V}} *{\widehat{\alpha }})(p)$ is bounded by a constant times $\Vert \, |\cdot | V \Vert _\infty$. To treat the other term, we go back to position space and note that

A.24

In combination with Lemma A.3, these considerations prove the claim. $\square$

The last lemma provides us with a convenient representation for the operator $K_{T,{{\textbf {A}}}}$. Its proof can be found in [15, Lemma 6.4]. Before we can state the lemma we need the following definition.

#### Definition A.6

*(Speaker path)* Let $R>0$, assume that $\mu \geqslant -1$ and define the following complex paths

The speaker path is defined as the union of paths $u_i$, $i=1, \ldots , 5$, with $u_1$ taken in reverse direction, i.e.,

If $\mu < -1$ we choose the same path as in the case $\mu = -1$.

#### Lemma A.7

Let $H:{\mathcal {D}}(H)\rightarrow {\mathcal {H}}$ be a self-adjoint operator on a separable Hilbert space ${\mathcal {H}}$ with $H\geqslant -\mu$ and let $\beta > 0$. Then, we have

A.25

with the speaker path  in Definition A.6. The above integral including the limit is understood as an improper Riemann integral with respect to the uniform operator topology.

In the following, we use the symbol  to denote the integral on the right side of (A.25) including the limit and we denote .

### Proof of Proposition A.1

The proof of Proposition A.1 is based on an adaption of gauge-invariant perturbation theory for Schrödinger operators as introduced in [42] to our setting. The core of the argument is contained in the following lemma.

#### Lemma A.8

Assume that $(1+|\cdot |^2) V \in L^{2}({\mathbb {R}}^3) \cap L^{\infty }({\mathbb {R}}^3)$. There is a continuous function $a :\rho (K_{T_{\text {c}}}-V) \rightarrow {\mathbb {R}}_+$ such that the following holds: For every compact set $K \subset \rho (K_{T_{\text {c}}}-V)$ there is a constant $h(K) > 0$ such that for $0 < h \leqslant h(K)$ we have

A.26 $$\begin{aligned} {\mathcal {R}}_{{\textbf {A}}}^{z, V} = {\mathcal {S}}_{{{\textbf {A}}}_h}^{z, V} + h^2 \, \eta _h(z) \quad \text { with } \quad \Vert (1+\pi ^2) \eta _h(z) \Vert _\infty \leqslant a(z). \end{aligned}$$

Here ${\mathcal {S}}_{{\textbf {A}}}^{z, V}$ denotes the operator defined by the kernel

A.27 $$\begin{aligned} {\mathcal {S}}_{{\textbf {A}}}^{z, V}(x,y)&{:}{=}\text {e}^{\text {i}\Phi _{{{\textbf {A}}}}(x,y)} \, {\mathcal {G}}^{z, V}(x,y) \end{aligned}$$

with the phase factor $\Phi _{{\textbf {A}}}(x, y)$ in (4.2).

#### Proof

We employ (4.18) and apply the integral representation in Lemma A.7 to $K_{{T_{\text {c}}}, {{\textbf {A}}}}$ to see that

A.28 $$\begin{aligned} K_{{T_{\text {c}}}, {{\textbf {A}}}}^x \, \text {e}^{\text {i}\Phi _{{{\textbf {A}}}_h}(x,y)} = \text {e}^{\text {i}\Phi _{{{\textbf {A}}}_h}(x,y)} \, K_{{T_{\text {c}}}, {{\textbf {A}}}_y}^x \end{aligned}$$

holds. Here $K_{{T_{\text {c}}}, {{\textbf {A}}}}^x$ is understood to act on the *x*-coordinate and ${{\textbf {A}}}_y(x) {:}{=}{\widetilde{{{\textbf {A}}}}}(x,y)$ denotes the vector potential in transversal Poincaré gauge relative to the point *y*, defined in (4.12). Using Lemma A.7 again, we also find the identity

A.29

where $\varphi (z) = (z/\tanh ( z/(2{T_{\text {c}}}) ) - z )$. Let us define the operator ${\mathcal {T}}_{{\textbf {A}}}^{z, V}$ via the equation

A.30 $$\begin{aligned} \left( z - (K_{{T_{\text {c}}}, {{\textbf {A}}}} - V) \right) {\mathcal {S}}_{{{\textbf {A}}}_h}^{z, V} = 1 - {\mathcal {T}}_{{{\textbf {A}}}_h}^{z, V}. \end{aligned}$$

Using (A.28), (A.29), and (A.30), we write the integral kernel of ${\mathcal {T}}_{{\textbf {A}}}^{z, V}$ as

A.31

In the next step we use this formula to prove a bound for the operator norm of ${\mathcal {T}}_{{\textbf {A}}}^{z, V}$.

Let us denote the first and the second term on the right side of (A.31) by ${\mathcal {T}}_{{\textbf {A}}}^{(1)}(x,y)$ and ${\mathcal {T}}_{{\textbf {A}}}^{(2)}(x,y)$, respectively. Using (4.24) and (4.25), we see that

A.32 $$\begin{aligned} | {\mathcal {T}}_{{{\textbf {A}}}_h}^{(1)}(x,y) | \leqslant Ch^2 \bigl ( |x-y| \, | \nabla _x {\mathcal {G}}^{z, V}(x,y) | + \bigl ( |x - y| + | x-y |^2 \bigr ) | {\mathcal {G}}^{z, V}(x,y) | \bigr ). \end{aligned}$$

Using the resolvent identity ${\mathcal {R}}^{z, V} = {\mathcal {R}}^z + {\mathcal {R}}^z V {\mathcal {R}}^{z, V}$, we estimate the first term on the right side of (A.32) by

A.33 $$\begin{aligned} \left| x-y \right| \left| \nabla _x {\mathcal {G}}^{z, V}(x,y) \right| \leqslant&\left| x-y \right| \left| \nabla {\mathcal {G}}^z( x-y) \right| \nonumber \\&+ \int _{{\mathbb {R}}^3} \text {d}w \ \left| |x-w| \, \nabla {\mathcal {G}}^z (x-w) \, V( w) \, {\mathcal {G}}^{z, V}(w,y) \right| \nonumber \\&+ \int _{{\mathbb {R}}^3} \text {d}w \ \left| \nabla {\mathcal {G}}^z (x-w) \, V(w) \, |w-y| \, {\mathcal {G}}^{z, V}(w,y) \right| . \end{aligned}$$

Eq. (A.33) allows us to obtain the following bound for the operator norm of ${\mathcal {T}}_{{\textbf {A}}}^{(1)}$:

A.34 $$\begin{aligned} \Vert {\mathcal {T}}_{{{\textbf {A}}}_h}^{(1)} \Vert _{\infty }&\leqslant Ch^2 \big [ \left\| |\cdot | \nabla {\mathcal {G}}^z \right\| _1 \left( 1 + \left\| V \right\| _2 \Vert {\mathcal {R}}^{z, V} \Vert _{2;\infty } \right) \nonumber \\&\quad + \left( 1+ \Vert \nabla {\mathcal {G}}^z \Vert _1 \left\| V \right\| _2 \right) \Vert [x, {\mathcal {R}}^{z, V} ] \Vert _{2;\infty } + \Vert [x,[x, {\mathcal {R}}^{z, V} ]] \Vert _{2;\infty } \big ]. \end{aligned}$$

From Lemmas A.3 and A.4, we know that there is a continuous $a :\rho (K_{T_{\text {c}}}-V) \rightarrow {\mathbb {R}}_+$ such that the right side of (A.34) is bounded by *a*(*z*). In the following we will denote by *a*(*z*) a generic function with these properties whose precise form may change from line to line.

To obtain a bound for the operator norm of ${\mathcal {T}}_{{\textbf {A}}}^{(2)}(z)$, we first estimate its kernel by

A.35

From Lemma 4.2 we know that the absolute value of the resolvent kernel of the magnetic Laplacian is bounded from above by a function only depending on $x-v$, whose $L^1({\mathbb {R}}^3)$-norm is bounded by a constant times $f( {{\,\mathrm{\text {Re}}\,}}\zeta , {{\,\mathrm{\text {Im}}\,}}\zeta )$ with *f* in (4.4). This, in particular, implies that this $L^1({\mathbb {R}}^3)$-norm is uniformly bounded in  and *h* as long as the latter is small enough, compare this to the bound in [15, Eq. (6.19)]. We use this bound, $| v-y | \leqslant | v-w | + | w - y |$, and the resolvent identity for ${\mathcal {R}}^{z, V}$ to bound the operator norm of ${\mathcal {T}}_{{\textbf {A}}}^{(2)}$ by

A.36

The constant on the right side is an affine function of each of its arguments. From Lemma 4.4 we know that the norms involving $g_0^{\zeta }$ are finite. In combination with Lemmas A.3 and A.4, this implies that the right side of (A.36) is bounded by *a*(*z*). We conclude that

A.37 $$\begin{aligned} \Vert {\mathcal {T}}_{{{\textbf {A}}}_h}^{z, V} \Vert _\infty \leqslant a(z)\, h^2 \end{aligned}$$

holds.

Let $K \subset \rho (K_{T_{\text {c}}}-V)$ be compact. The above bounds allow us to find $h_0(K) > 0$ such that for $z \in K$ and as long as $0< h < h_0(K)$ we can write the resolvent of $K_{{T_{\text {c}}}, {{\textbf {A}}}}-V$ as

A.38 $$\begin{aligned} \frac{1}{z - (K_{{T_{\text {c}}}, {{\textbf {A}}}} - V)} = {\mathcal {S}}_{{{\textbf {A}}}_h}^{z, V} + h^2 \, \eta _h(z) \quad \text { with } \quad \eta _h(z) {:}{=}h^{-2} {\mathcal {S}}_{{{\textbf {A}}}_h}^{z, V} \sum _{n=1}^{\infty } ({\mathcal {T}}_{{{\textbf {A}}}_h}^{z, V})^n. \end{aligned}$$

To show that the operator norm of $(1 + \pi ^2) \eta _h(z)$ is bounded by *a*(*z*), we use

A.39 $$\begin{aligned} \Vert (1 + \pi ^2) \eta _h(z) \Vert _\infty \leqslant \Vert (1 + \pi ^2) {\mathcal {S}}_{{{\textbf {A}}}_h}^{z, V} \Vert _\infty \; \sum _{n=1}^{\infty } h^{2(n-1)} a(z)^n. \end{aligned}$$

With the resolvent identity for ${\mathcal {R}}^{z, V}$ and Lemma A.3, we easily see that the operator norm of $(1 + \pi ^2) {\mathcal {S}}_{{{\textbf {A}}}_h}^{z, V}$ is bounded by *a*(*z*). This proves the claim. $\square$

With the resolvent estimates in Lemma A.8 at hand, we turn to the proof of Proposition A.1. Let $\lambda < 2{T_{\text {c}}}$ be an eigenvalue of the operator $K_{T_{\text {c}}}- V$. Our assumption on *V* guarantees that it has finite multiplicity $m \in {\mathbb {N}}$. We choose $\varepsilon > 0$ such that the ball $B_{\varepsilon }(\lambda ) \subseteq {\mathbb {C}}$ contains no other point of the spectrum of $K_{T_{\text {c}}}- V$ than $\lambda$ and define

A.40 $$\begin{aligned} P(h) {:}{=}\int _{\partial B_{\varepsilon }(\lambda )} \frac{\text {d}z}{2 \pi \text {i}} \; {\mathcal {R}}_{{\textbf {A}}}^{z, V} = \int _{\partial B_{\varepsilon }(\lambda )} \frac{\text {d}z}{2 \pi \text {i}} \; {\mathcal {S}}_{{{\textbf {A}}}_h}^{z, V} + h^2 \int _{\partial B_{\varepsilon }(\lambda )} \frac{\text {d}z}{2 \pi \text {i}} \; \eta _h(z). \end{aligned}$$

From Lemma A.8 we know that the operator norm of the second term on the right side is bounded by a constant times $h^2$ provided *h* is small enough. The integral kernel of the first term is given by

A.41 $$\begin{aligned} \Bigl ( \int _{\partial B_{\varepsilon }(\lambda )} \frac{\text {d}z}{2 \pi \text {i}} \; {\mathcal {S}}_{{\textbf {A}}}^{z, V} \Bigr )(x,y) = \text {e}^{\text {i}\Phi _{{{\textbf {A}}}}(x,y)} \sum _{i=1}^m u_i(x) \overline{ u_i(y) }, \end{aligned}$$

where the vectors $\{ u_i \}_{i=1}^m$ span the eigenspace of $\lambda$. Let us denote by *P* the projection onto that linear space. Using (A.40), (A.41), and $|\Phi _{{{\textbf {A}}}_h}(x,y)| \leqslant C h^2 (|x|^2 + |y|^2)$, which follows from the assumption $A(0) =0$, we obtain the bound

A.42 $$\begin{aligned} \Vert P(h) - P \Vert _{\infty } \leqslant C h^2 \, \max _{i=1, \ldots ,m} \Vert \, |\cdot |^2 u_i \Vert _2. \end{aligned}$$

In combination with Lemma A.5, this proves ${{\,\textrm{rank}\,}}P(h) = m$ for *h* small enough as well as the second bound in (A.1).

To prove the bounds for the eigenvalues we use the identity

A.43 $$\begin{aligned} (K_{{T_{\text {c}}}, {{\textbf {A}}}} - V) P(h) = \int _{\partial B_{\varepsilon }(\lambda )} \frac{\text {d}z}{2 \pi \text {i}} \; z \, {\mathcal {R}}_{{\textbf {A}}}^{z, V} . \end{aligned}$$

As long as *h* is small enough, the rank of this operator equals *m* and its eigenvalues are given by $\lambda _1(h), \ldots , \lambda _m(h)$. Similar arguments to the above for the spectral projections allow us to conclude that

A.44 $$\begin{aligned} \Vert (K_{{T_{\text {c}}}, {{\textbf {A}}}} - V) P(h) - (K_{T_{\text {c}}}- V) P \Vert _{\infty } \leqslant C h^2 \, \max _{i=1, \ldots ,m} \Vert \, |\cdot |^2 u_i \Vert _2 \end{aligned}$$

holds. This proves the claimed bound for the eigenvalues. It remains to prove (A.2).

Let us write $\alpha _*^h = a(h) \alpha _* + b(h) \phi _h$ with $\langle \alpha _*, \phi _h \rangle = 0$ and $|a(h)|^2 + |b(h)|^2 = 1$. Our assumptions imply $a(h) = \langle \alpha _*, \alpha _*^h \rangle \geqslant 0$. We rewrite the equation $P(h) \alpha _*^h = \alpha _*^h$ to see that $b(h) \phi _h = (P(h)-P) \alpha _*^h$. An application of (A.1) thus implies $|b(h)| \leqslant C h^2$. Using this, $|a(h)|^2 + |b(h)|^2 = 1$, and the fact that $a(h) \geqslant 0$, we see that $| a(h) - 1 | \leqslant C h^2$. This allows us to conclude that

A.45 $$\begin{aligned} \Vert (1+\pi ^2) ( \alpha _*^h - \alpha _* ) \Vert _2 \leqslant |a(h)-1| \ \Vert (1 + \pi ^2) \alpha _* \Vert _2 + | b(h) | \ \Vert (1 + \pi ^2) \phi _h \Vert _2 \leqslant C h^2. \end{aligned}$$

To obtain the result, we used Lemma A.5 to see that $\Vert (1 + \pi ^2) \alpha _* \Vert _2 < \infty$, as well as $\Vert (1 + \pi ^2) \phi _h \Vert _2 \leqslant \Vert (1 + \pi ^2) \alpha _* \Vert _2 + \Vert (1 + \pi ^2) \alpha _*^h \Vert _2$, and

A.46 $$\begin{aligned} \Vert (1+\pi ^2) \alpha _*^h \Vert _2 \leqslant C \Vert K_{{T_{\text {c}}}, {{\textbf {A}}}} \alpha _*^h \Vert _2 \leqslant C \left( | \lambda (h) | + \Vert V \alpha _*^h \Vert _2 \right) \leqslant C \left( | \lambda (h) | + \Vert V \Vert _\infty \right) . \end{aligned}$$

This proves (A.2) and also finishes the proof of Proposition A.1.
